# High-affinity binding at quadruplex–duplex junctions: rather the rule than the exception

**DOI:** 10.1093/nar/gkac1088

**Published:** 2022-11-23

**Authors:** Yoanes Maria Vianney, Klaus Weisz

**Affiliations:** Institute of Biochemistry, Universität Greifswald, Felix-Hausdorff-Str. 4, D-17489 Greifswald, Germany; Institute of Biochemistry, Universität Greifswald, Felix-Hausdorff-Str. 4, D-17489 Greifswald, Germany

## Abstract

Quadruplex-duplex (Q–D) junctions constitute unique structural motifs in genomic sequences. Through comprehensive calorimetric as well as high-resolution NMR structural studies, Q–D junctions with a hairpin-type snapback loop coaxially stacked onto an outer G-tetrad were identified to be most effective binding sites for various polycyclic quadruplex ligands. The Q–D interface is readily recognized by intercalation of the ligand aromatic core structure between G-tetrad and the neighboring base pair. Based on the thermodynamic and structural data, guidelines for the design of ligands with enhanced selectivity towards a Q–D interface emerge. Whereas intercalation at Q–D junctions mostly outcompete stacking at the quadruplex free outer tetrad or intercalation between duplex base pairs to varying degrees, ligand side chains considerably contribute to the selectivity for a Q–D target over other binding sites. In contrast to common perceptions, an appended side chain that additionally interacts within the duplex minor groove may confer only poor selectivity. Rather, the Q–D selectivity is suggested to benefit from an extension of the side chain towards the exposed part of the G-tetrad at the junction. The presented results will support the design of selective high-affinity binding ligands for targeting Q–D interfaces in medicinal but also technological applications.

## Graphical Abstract

**Graphical Abstract:**
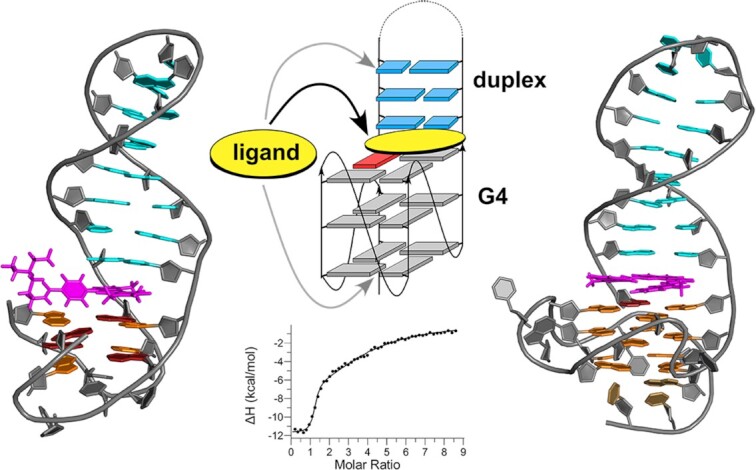
A set of typical quadruplex ligands recognize quadruplex–duplex junctions through intercalation at the interface of the two structural domains.

## INTRODUCTION

G-rich sequences can fold into non-canonical secondary structures called G-quadruplexes (G4s) (1,2). In these tetra-stranded structures, four guanine bases are positioned in a square planar arrangement and linked by a cyclic array of Hoogsteen hydrogen bonds. In general, G4s comprise a stack of two to four of such G-tetrads with monovalent cations like Na^+^ or K^+^ coordinated within the central channel of the G-core for additional stabilization. Due to the abundance of G-rich sequences at critical locations within the genome such as in telomeres and oncogenic promoters and also sparked by the observation that G4 formation interferes with cellular processes like telomere maintenance or gene transcription, these alternative nucleic acid structures have been recognized as promising targets for medicinal interventions through selective binding of small drug molecules (3,4). This prompted the design and screening of a plethora of G4 binding ligands, some of them exhibiting a remarkable affinity with dissociation constants in the micromolar and even sub-micromolar range (5,6). On the other hand, the majority of ligands reported to date comprises a polycyclic aromatic ring system and primarily bind through stacking on outer G-tetrads with only weak additional interactions with loop and/or overhang residues (4,7). Consequently, selectivities against other competing G4 topologies but also against genomic B-type duplexes are mostly poor, allowing for putative off-target effects in potential therapeutic applications.

In contrast, non-canonical G4 structures with unique structural motifs (8) but especially quadruplex–duplex (Q–D) junctions may provide for a more selective high-affinity targeting through low molecular weight compounds. Additionally, they may also be used as powerful tools to enforce a particular G4 topology or to optimize aptamer affinities (9–13). Q–D junctions are expected to frequently occur within the genome (14–16). They may involve external double-helical overhang sequences or internal duplex stem loops with or without unpaired linker nucleotides between G4 and duplex domains. The orientation of duplex and G4 helices may either be coaxial or orthogonal, depending on the attachment of the duplex to the G4 core. Seminal studies by Phan et al. on the structure and thermodynamics of several engineered Q–D hybrids have revealed continuous stacking at the interface if there is a coaxial orientation of directly linked quadruplex and duplex domains, with different stacking energies depending on the type of base pair at the junction (17,18).

Notably, systematic studies on the ligand binding at Q–D interfaces are still in their infancy. Early reports on the recognition of an RNA Q–D motif involved the positively charged RGG peptide from the human fragile X mental retardation protein (FMRP), binding to the major groove of the duplex domain (19,20). A rational approach for targeting a Q–D motif proposed the design of a two-component hybrid molecule composed of a quadruplex-specific ligand with an extended aromatic surface area and a duplex-specific minor groove binder. Although the simultaneous recognition by both ligand moieties was demonstrated, a more detailed structural characterization has not been provided (21,22). Very recently, the binding of simple aminomethyl-substituted aromatic hydrocarbons as well as indoloquinoline, naphthalene diimide, and pyridostatin derivatives to Q–D junctions resulted in first high-resolution structures of corresponding complexes (23–26). In fact, indoloquinoline-based ligands such as unsubstituted cryptolepine, aminoalkylated SYUIQ-5, and a phenyl-substituted indoloquinoline derivative PIQ-4m recognized the Q–D junction with higher affinity compared to either the free quadruplex or free duplex, suggesting the possibility of a successful ligand design dedicated for selective Q–D targeting (25,27). For SYUIQ-5, the indoloquinoline aromatic ring system intercalates at the Q–D interface of a parallel G4 with a coaxially stacked hairpin-type snapback loop while the positively charged side chain extends into the minor groove of the duplex (25). A similar binding mode was also reported for the naphthalene diimide derivative (24). Here, the naphthalene diimide plane inserts between an outer G-tetrad of a hybrid G4 and a coaxially stacked lateral duplex stem-loop with the platinum-containing side chain positioned within the duplex minor groove. Likewise, the *N*-methylated indoloquinoline cryptolepine, lacking an additional side chain, preferred to bind through intercalation at the Q–D interface of a G4 with a coaxially stacked duplex domain, yet in a more flexible binding mode with enhanced dynamics (25). This hints at the importance of π-π stacking as a major driving force of Q–D recognition.

**Figure 1.**
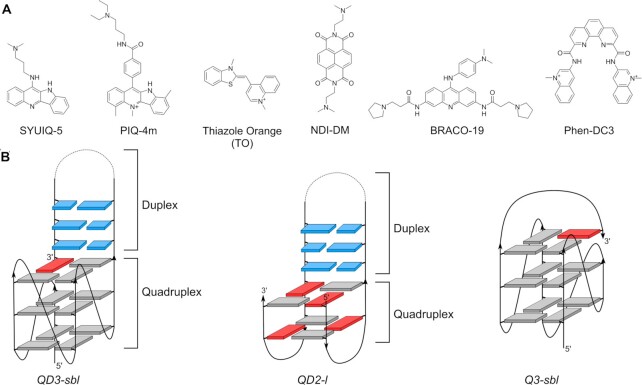
(**A**) Chemical structure of quadruplex binding ligands. (**B**) Topologies of quadruplexes with and without a duplex interface; *anti*- and *syn*-guanosines of the G-core are colored grey and red, respectively, Watson–Crick base pairs are colored blue.

To provide a more solid basis for targeting Q–D junctions, we here report on a comprehensive thermodynamic and structural study involving several typical G4 binding ligands (Figure 1A). The combination of comparative calorimetric binding experiments using tailored nucleic acid receptors with NMR structural studies on selected complexes identifies the Q–D junction as primary binding site with the largest association constant for most of the tested ligands. Consequently, the favored recognition of Q–D interfaces constitutes a general rather than a more specific phenomenon. However, discrimination of the Q–D junction against intercalation into a B-type duplex or end stacking on an exposed outer G-tetrad widely varies. Whereas the typical G4 ligand SYUIQ-5 suffers from a poor selectivity in the presence of longer duplex domains, PIQ-4m seems superior in terms of its discriminatory power in favor of the Q–D interface. Based on the collected data, a ligand design for most effective Q–D recognition is proposed, supporting future efforts in targeting G4 structures through existing Q–D motifs.

## MATERIALS AND METHODS

### Sample preparation

DNA oligonucleotides were purchased from TIBMOLBIOL (Berlin, Germany) and further purified through ethanol precipitation prior to their use. Concentrations of oligonucleotides were determined by measuring their absorbance *A*_260_ at 80°C in aqueous solution using extinction coefficients as provided by the manufacturer. PIQ derivatives were prepared as described and their concentration determined spectrophotometrically using a molar extinction coefficient *ϵ*_376_ of 22 227 M^−1^·cm^−1^ in potassium phosphate buffer (28,29). Thiazole orange (TO), SYUIQ-5, BRACO-19 and Phen-DC3 were purchased from Sigma-Aldrich Chemie GmbH (Taufkirchen, Germany). NDI-DM was obtained from ABCR (Karlsruhe, Germany). Thiazole orange concentrations were determined using a molar extinction coefficient for the TO monomer *ϵ*_500_ of 63 000 M^−1^·cm^−1^ in DMSO (30). Concentrations of all other ligands were determined from their weighed mass. All ligands were initially dissolved in a DMSO stock solution except for isothermal titration calorimetry (ITC) measurements. Here, ligands were directly dissolved in the high-salt ITC buffer solution (20 mM potassium phosphate, 100 mM KCl, pH 7.0, supplemented with 5% DMSO). A low-salt buffer with 10 mM potassium phosphate, pH 7.0, was additionally employed for circular dichroism (CD) melting and some of the NMR experiments as described.

### UV melting experiments

To confirm complete folding under the ITC experimental conditions, melting of DNA receptors (2–5 μM depending on duplex or quadruplex melting experiments) was evaluated in the ITC buffer solution with a Jasco V-650 spectrophotometer equipped with a Peltier thermostat. For melting of the duplex and quadruplex domains, temperature-dependent absorbances *A*_260_ and *A*_295_ were recorded using a heating rate of 0.2°C·min^-1^ and a bandwidth of 1 nm. Melting temperatures *T*_m_ were determined in triplicate from the first derivative of the absorbance versus temperature plot.

### ITC experiments

ITC experiments were performed at 40°C with a Microcal PEAQ-ITC microcalorimeter using a reference power of 4 μcal·s^−1^. Oligonucleotides and ligands were dissolved in ITC buffer and the ligand (400 μM) was titrated to the oligonucleotide (20 μM) with a total of 2 × 26 injections. Titration volumes, duration of injections, and spacing between injection steps were 1.5 μl, 3 s, and 240 s, respectively. The first injection (0.4 μl) was rejected for data analysis through the Microcal PEAQ-ITC analysis software. Generally, data were fitted with a model of two sets of independent binding sites. Additional excess-site titrations were performed for a model-independent determination of binding enthalpies. Here, ligand (3 μl, 200 μM) was titrated in 12 injection steps to an oligonucleotide solution (100 μM) with an injection duration of 6 s and a spacing between injections of 300 s. The first injection (0.4 μl) was discarded for the determination of an average binding enthalpy. All experiments were blank- and concentration-corrected and done in triplicate.

### CD spectroscopy

CD spectra were recorded at 30°C with a Jasco J-810 spectropolarimeter equipped with a Peltier thermostat in a high-salt buffer. Ligands in a DMSO stock solution were titrated to the Q–D hybrid (5 μM) up to a 4:1 ligand-to-DNA molar ratio with final DMSO concentrations ≤1.1%. Ellipticities were recorded from 230 nm up to 600 nm for the TO ligand using a bandwidth of 1 nm, a scanning speed of 50 nm·min^−1^, a response time of 4 s, and five accumulations. All spectra were blank-corrected. For CD melting experiments, the *QD2-l* hybrid was dissolved in 10 mM potassium phosphate buffer, pH 7.0 (2 ml, 5 μM). For melting of the complex, a concentrated ligand solution in DMSO was titrated to give a 1:1 molar ratio. Ellipticities at 295 nm were recorded from 15°C to 95°C with a heating rate of 0.2°C·min^−1^ and a bandwidth of 1 nm. Melting temperatures were determined by the first derivative of the melting curve and averaged over two independent experiments.

### NMR spectroscopy

NMR spectra were acquired on a Bruker Avance NEO 600 MHz spectrometer equipped with an inverse ^1^H/^13^C/^15^N/^19^F quadruple resonance cryoprobehead and z-field gradients. Spectra were processed in TopSpin 4.0.7 and assigned in CcpNMR V2 (31). Oligonucleotides were dissolved in either a low-salt or high-salt buffer with a 90% H_2_O/10% D_2_O solvent system. Ligands were added in a DMSO-d_6_ stock solution with a final DMSO concentration for a 1:1 mixture of ≤4%. Proton chemical shifts were referenced to sodium trimethylsilylpropionate (TSP) through the temperature-dependent water chemical shift at pH 7 while ^13^C chemical shifts were referenced to sodium trimethylsilylpropanesulfonate (DSS) through indirect referencing. For further details on NMR experimental parameters see the Supplementary Information.

### Structure calculations

Structures of complexes between *QD3-sbl* and Phen-DC3 as well as between *QD2-l* and PIQ-4m were initially generated with a simulated annealing protocol using XPLOR-NIH 3.0.3 (32). Experimental restraints employed for the calculations included distances as derived from NOESY crosspeak intensities, glycosidic torsion angles *χ*, sugar puckers from DQF-COSY crosspeak patterns, as well as hydrogen bond and planarity restraints (see the Supplementary Information for more details). Additional chirality restraints were imposed in calculations of the *QD3-sbl*–Phen-DC3 complex. In the following, 100 out of 400 calculated structures were selected for a structural refinement using AMBER18 with the parmbsc0 force field and OL15 modifications according to a protocol described recently (25). In short, having established a force field for the ligand, starting structures were subjected to simulated annealing with experimental and planarity restraint energies to yield 20 lowest-energy structures. For a refinement in explicit water, the DNA was initially neutralized and potassium ions placed within the inner core of the G-quadruplex flanked by the tetrad layers. A final simulation was done at 1 atm and 300 K for 4 ns using only NOE- and hydrogen bond-based distance restraints. The trajectory was averaged for the last 500 ps. It should be mentioned that this averaging process resulted in noticeable distortions of the highly flexible PIQ-4m aliphatic side chain but was eliminated through final minimizations, yielding ten lowest-energy structures. Pymol 2.3.2 was used for visualization and the extraction of conformational parameters.

## RESULTS AND DISCUSSION

### Ligands and DNA receptors

For getting a deeper insight into the binding affinity and binding mode of G4 ligands when associating with quadruplexes featuring a Q–D junction, a set of six ligands comprising different polycyclic aromatic core structures with up to three side chains were selected for biophysical studies (Figure 1A). With their flat aromatic heterocyclic ring systems prone to π–π stacking interactions, indoloquinolines SYUIQ-5 (33,34) and PIQ-4m (28,29), naphthalene diimide NDI-DM (35,36), trisubstituted acridine BRACO-19 (37,38), and phenanthroline derivative Phen-DC3 (39,40) are all considered typical G4 binding ligands with a preference to stack on exposed outer G-tetrads but also with varying propensities to serve as duplex intercalators. Thus, whereas Phen-DC3 exhibits good selectivity for G4s in comparison with duplexes but shows only poor selectivity against different G4 topologies, BRACO-19 was reported to also show significant binding to duplex structures (41). On the other hand, thiazole orange (TO), frequently applied as fluorescent probe in nucleic acid sensing (42), is regarded a more universal DNA intercalator with a rather poor discriminatory ability for nucleic acid secondary structures.

To probe binding of the ligands at Q–D junctions, the *QD3-sbl* hybrid was employed as DNA receptor. It comprises a three-layered parallel G4 and a 3′-duplex stem loop that is fixed to the interfacial tetrad by the terminal G, filling a vacant tetrad position along the first G-column (Figure 1B, Table 1) (25). Such an architecture showcases different putative binding sites, i.e. the Q–D junction, the exposed 5′-outer tetrad, and the duplex domain. While *QD3-sbl* allows competition among different binding sites, additional truncated constructs were designed to facilitate separation of binding processes in thermodynamic studies. Thus, *Q3-sbl* preserves the 5′-outer tetrad of the *QD3-sbl* hybrid but lacks the coaxially stacked double-helical 3′-extension. Instead, the 3′-outer tetrad is bridged by a diagonal snapback loop known to effectively block ligand binding to only feature the 5′-tetrad as a putative high-affinity binding site (40,43,44). Likewise, only employing the duplex hairpin of *QD3-sbl* termed *D3-HP* allows exclusive extraction of binding parameters for the double-helical domain.

**Table 1.** DNA sequences used in the present study; G residues in tetrads are underlined

Name	Sequence (5′-3′)
*QD3-sbl*	TTAGGTGGGTAGGGTGGG-CTAGTCATTTTGACTAG-G
*Q3-sbl*	TTAGGGTGGTAGGGTGGG-GAAG-G
*D3-HP*	CTAGTCATTTTGACTAG
*QD2-l*	GGTTGG-CGCGAAGCATTCGCG-GGTTGG
*D2-HP*	CGCGAAGCATTCGCG
*QD2-l-2bp*	GGTTGG-CGGCACG-GGTTGG
*TBA*	GGTTGG-TGT-GGTTGG
*Q3-sbl2*	TTAGGTGGGTAGGGTGGG-TGT-G

In addition to the Q–D hybrid based on a parallel G4 topology, another Q–D hybrid *QD2-l* is based on a two-layered chair-type antiparallel quadruplex originally designed by Phan (Figure 1B, Table 1) (17). Here, the second lateral loop of the thrombin binding aptamer *TBA* (45) spanning the G4 wide groove has been replaced by a duplex stem loop. Because the tetrad opposite the Q–D junction is occluded by two TT lateral loops, only binding at the junction or within the duplex domain is expected to compete with each other in this case. Finally, cutting the length of the duplex stem loop in *QD2-l-2bp* or replacing it by a non-base-paired TGT lateral loop as for parent *TBA* or *Q3-sbl2* is expected to give additional information on the structural requirements for ligand binding at Q–D interfaces.

### Calorimetry points to the quadruplex–duplex interface of *QD3-sbl* as a binding hotspot for the tested ligands

Although several studies in the past years have reported association constants for some of the ligands upon binding G4 structures, a reasonable comparison of data is often impossible due to the use of different temperatures, buffer conditions, and/or G4 sequences (46–49). Isothermal titration calorimetry (ITC) was therefore employed for extracting information on potential ligand binding sites and for a direct comparison of detailed thermodynamic profiles. To match previously reported conditions (25,27), titrations were performed at a temperature of 40°C in a buffer solution with 120 mM K^+^ ions close to a physiological environment. This high-salt and high-temperature setup was also expected to repress unspecific electrostatic interactions upon binding the cationic ligands. The buffer was supplemented with 5% DMSO to overcome solubility limitations for some of the compounds. However, even in the presence of the DMSO additive, Phen-DC3 was not amenable to calorimetric measurements due to persisting solubility problems even when trying to set up a reverse titration experiment with the ligand used in a lower-concentrated titrand solution.

Initially, ligands were titrated to the *QD3-sbl* hybrid. More complex binding equilibria can be expected given three different putative binding sites competing for the ligand. ITC curve fitting routines with three or even more sets of binding sites have been employed in the past but in many cases suffer from the large number of free-floating and often interdependent parameters (50). Here, the curve fitting routine was based on a maximum of only two sets of independent binding sites to possibly result in ambiguities of binding parameters in some cases. Also, because lower-affinity binding sites are often less well defined, only parameters for high-affinity binding are discussed in the following (for a compilation of all fit parameters see Supplementary Table S1). Being confronted with these shortcomings when attempting to separate different binding processes, additional titrations were performed on the quadruplex *Q3-sbl* and the hairpin duplex *D3-HP*, expected to closely mimic the exposed tetrad and hairpin duplex of the *QD3-sbl* hybrid, respectively. Before measurements, all receptors including the free duplex hairpin were shown by UV melting to be folded under the ITC experimental conditions (see Supplementary Table S2).

**Figure 2.**
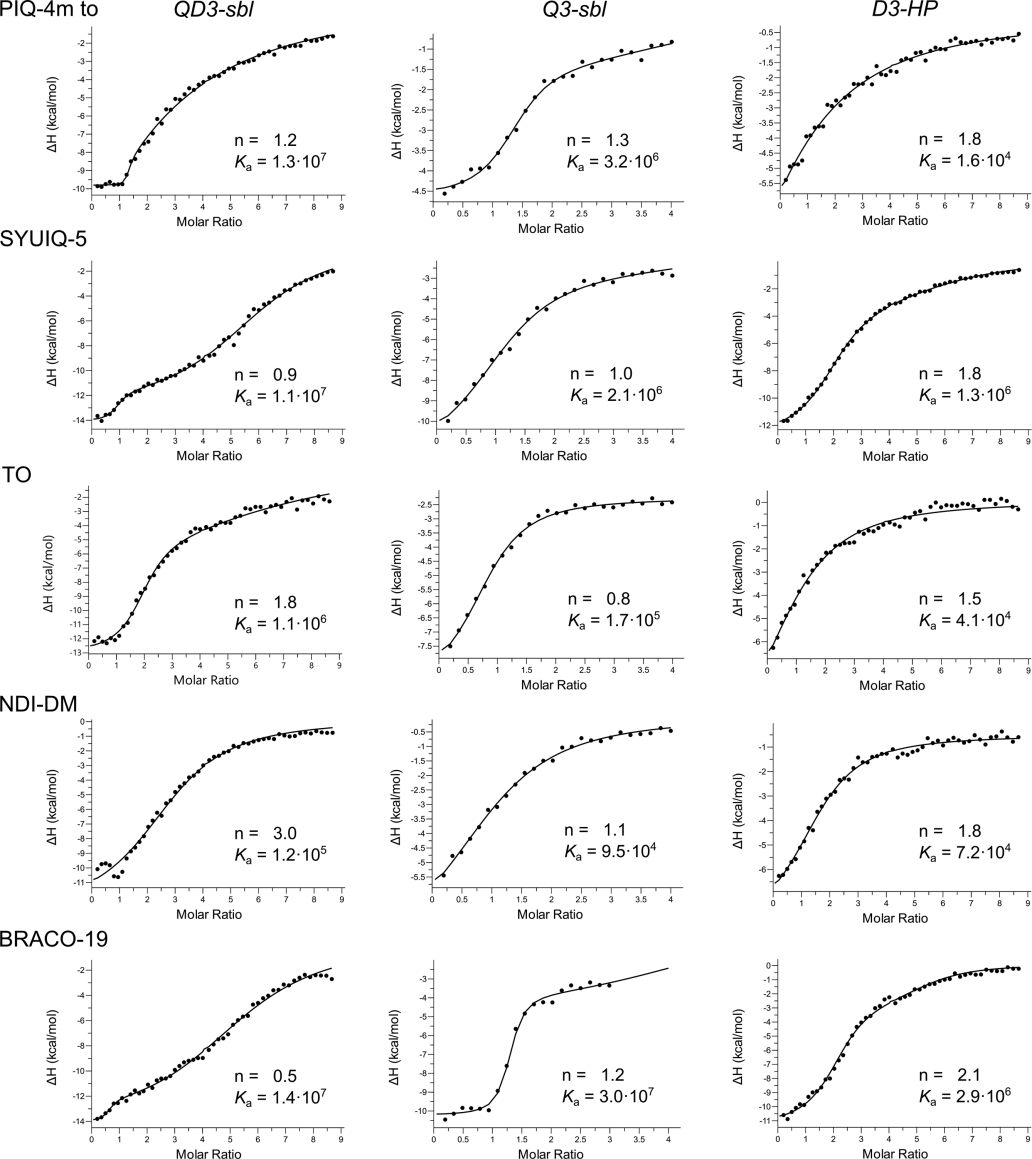
ITC thermograms for G4 ligands titrated to *QD3-sbl, Q3-sbl* and *D3-HP* at 40°C in a 120 mM K^+^ buffer; stoichiometries n and affinity constants *K*_a_ (in M^-1^) as determined by curve fitting are indicated. For a compilation of all fit parameters with root-mean-square deviations from three independent experiments see Supplementary Table S1.

There is a wealth of information gained from the ITC titrations of the ligands to each of the *QD3-sbl*, *Q3-sbl*, and *D3-HP* receptors (Figure 2). Results can be summarized as follows:

- Except for NDI-DM but in particular for BRACO-19 with their comparable or supposedly even higher affinity towards *Q3-sbl*, ligands bind with highest affinity to the *QD3-sbl* hybrid, indicating the Q–D junction to be the favored binding site. Association constants at 40°C vary in a narrow range between 1.1·10^7^ and 1.4·10^7^ M^−1^ for PIQ-4m, SYUIQ-5 and BRACO-19 but decrease by one and two orders of magnitude for TO and NDI-DM, respectively.
- Selectivities towards a particular binding site vary significantly among the ligands. Thus, the binding constant of the indoloquinoline PIQ-4m decreases by a factor of four when going from the *QD3-sbl* hybrid to the *Q3-sbl* parallel G4 and by three orders of magnitude when binding the corresponding free hairpin duplex *D3-HP*. Considering experimental uncertainties, selectivities between G4 hybrid and parallel G4, i.e. between a Q–D junction and an exposed outer tetrad, follow the order PIQ-4m ∼ SYUIQ-5 ∼ TO > NDI-DM ∼ BRACO-19. On the other hand, selectivities of the hybrid against the short hairpin duplex are given by PIQ-4m >> TO > SYUIQ-5 ∼ BRACO-19 > NDI-DM. Consequently, PIQ-4m seems superior in differentiating the junction against additional binding sites at both G4 and duplex domains whereas NDI-DM with its two side chains fails to be a selective G4 binder in the absence but also the presence of a Q–D junction. Interestingly, TO, regarded as universal DNA ligand, exhibits significant discriminating potential with clear preference of the Q–D junction, albeit with only moderate affinity.
- Given a much better quadruplex–duplex selectivity of the phenyl-substituted PIQ-4m when compared to SYUIQ-5 with its aliphatic side chain, it is conspicuous that G4 binding is considerably more exothermic for SYUIQ-5 irrespective of the DNA receptor (Supplementary Table S1). This is compensated by a much smaller entropic penalty for G4 binding of PIQ-4m. These contrasting profiles point to different interactions of the indoloquinoline side chains in PIQ-4m and SYUIQ-5 with more specific contacts of the flexible aliphatic side chain in SYUIQ-5 within a duplex or quadruplex groove.
- Whereas high-affinity binding to *QD3-sbl* is associated with a 1:1 stoichiometry for the two indoloquinolines, binding of TO as a dimer is indicated by the determined stoichiometries. However, stoichiometries of 3 and 0.5 for high-affinity binding to *QD3-sbl* in case of NDI-DM and BRACO-19 likely reflect the inability of the binding model to accurately fit and separate competing binding processes of similar affinities at initial titration steps.

### High-affinity binding depends on an interfacial base pair

**Figure 3.**
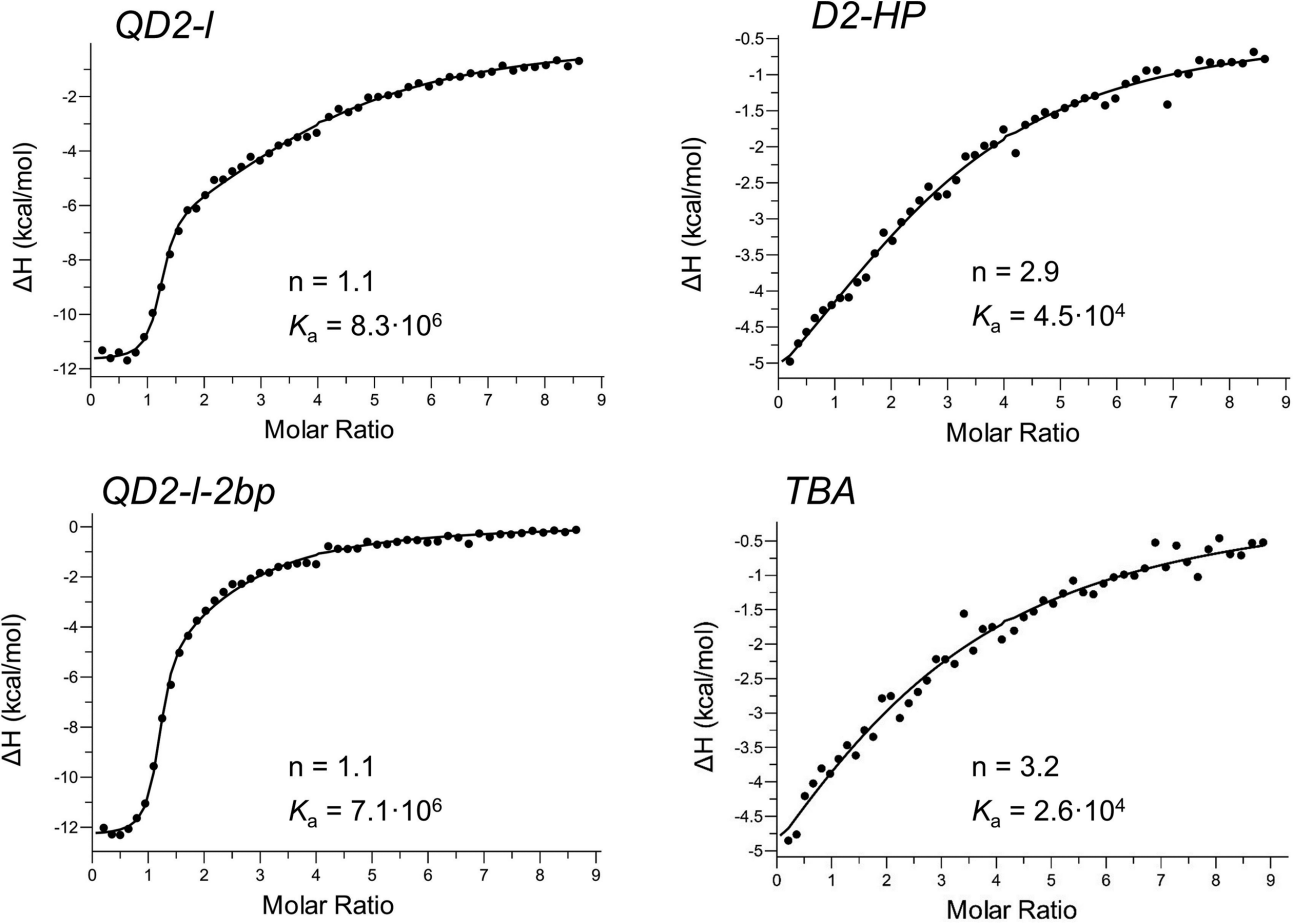
ITC thermograms for the PIQ-4m ligand titrated to *QD2-l, QD2-l-2bp, D2-HP* and *TBA* at 40°C in a 120 mM K^+^ buffer; stoichiometries *n* and affinity constants *K*_a_ (in M^-1^) as determined by curve fitting are indicated. For a compilation of all fit parameters with root-mean-square deviations from three independent experiments see Supplementary Table S3.

Additional binding studies on a hybrid *QD2-l* featuring a lateral duplex stem loop in a two-layered antiparallel G4 were performed with PIQ-4m as being the most selective Q–D binder (Figure 3). Here, a high affinity with *K*_a_ close to 10^7^ M^−1^ slightly lower compared to binding to the *QD3-sbl* hybrid was determined. However, there is a steeper rise in the thermogram after the addition of one equivalent of ligand and the saturation of the first binding site. Because access to the opposite outer tetrad should be effectively blocked through the two TT lateral loops in *QD2-l*, such a behavior is expected if only the duplex domain is left as competing binding site. Again, the latter shows rather weak binding as demonstrated by the ITC titration of the free hairpin duplex *D2-HP* with association constants lower by more than two orders of magnitude when compared to the high-affinity binding for the hybrid receptor.

Next, the duplex domain was truncated to a minimal duplex stem loop comprising only two potential base pairs. With the formation of a stable interfacial base pair as demonstrated by NMR (Supplementary Figure S1), the strength of high-affinity PIQ-4m binding was largely conserved (Figure 3). Clearly, a faster return to baseline at later titration steps results from the reduced number of low-affinity binding sites within the duplex domain. Finally, the parent TBA quadruplex with a non-duplex TGT lateral loop replacing the duplex stem loop was tested as receptor for PIQ-4m binding. Here, binding was very weak and did not even exceed affinities for a free duplex, demonstrating that only an intact base pair at the interface seems to allow strong interactions of the ligand at the Q–D junction.

As mentioned above, no corresponding ITC data could be extracted for Phen-DC3 owing to its poor solubility in the used buffer. To nevertheless obtain an estimate of its binding affinity towards a G4 hybrid, CD melting studies on 1:1 mixtures of the hybrid receptor with the ligand were performed and compared to indoloquinolines SYUIQ-5 and PIQ-4m. Because of limitations set by the high melting in case of *QD3-sbl*, the *QD2-l* receptor with a melting temperature *T*_m_ of 50.3°C was employed under low-salt conditions, providing for a convenient temperature window for all melting experiments (Supplementary Figure S2). Thus, 1:1 complexes with SYUIQ-5 and PIQ-4m yielded a *T*_m_ increase by ∼5°C and ∼13°C, respectively. The noticeably smaller thermal stability of the SYUIQ-5 compared with the PIQ-4m complex may mostly be derived from the more enthalpy-driven SYUIQ-5 association given the general temperature-dependence of association. Remarkably, with a *T*_m_ of 74°C binding of 1 equivalent of Phen-DC3 resulted in a considerable increase of the *QD2-l* melting temperature by almost 24°C. Consequently, Phen-DC3 seems to surpass all other tested ligands in terms of binding affinity towards the hybrid structure, assuming the melting temperature to be a reasonable measure of the binding free energy.

### Tracking high-affinity binding sites through NMR experiments

Whereas indoloquinoline ligands and a naphthalene diimide derivative were recently shown to bind at Q–D junctions (24,25,27), there is no structural information on favored binding sites of the other G4 ligands when targeting the Q–D hybrid. Clearly, different affinities but also exothermicities obtained for initial binding events on the *QD3-sbl* hybrid when compared to the *Q3-sbl* G4 or *D3-HP* duplex fragment suggest that the Q–D junction mostly outcompetes outer G-tetrad stacking or duplex binding. To further validate this conclusion and to exclude putative interfering phenomena such as significant cooperativity effects between binding sites and/or ligand-induced G4 un/refolding, additional structural studies were performed.

Initially, ligand titration to *QD3-sbl* was followed by CD spectroscopy. In addition to induced CD (ICD) effects at the ligand absorption wavelength, only small to moderate changes in the CD amplitudes of minima and maxima at about 240 and 265 nm were observed. This suggests no major G4 conformational rearrangements upon ligand binding (Supplementary Figure S3). ICD effects show ligand-specific shapes and intensities. Although the sign of the ICD is affected by the specific orientation of the bound ligand through its transition dipole moment, ICDs are notoriously difficult to interpret in terms of binding mode or even of a defined binding geometry. A weak negative ICD as observed throughout the titration for NDI-DM or Phen-DC3 has often been associated with duplex intercalation but also tetrad stacking (51,52). On the other hand, bisignate ICDs as seen for most other ligands when added in excess may either be attributed to exciton couplings of two or more ligands bound in close proximity or to different binding sites with CD signals of opposite sign and shift. Notably, TO already starts to develop a bisignate ICD before exceeding a 1:1 molar ratio.

**Figure 4.**
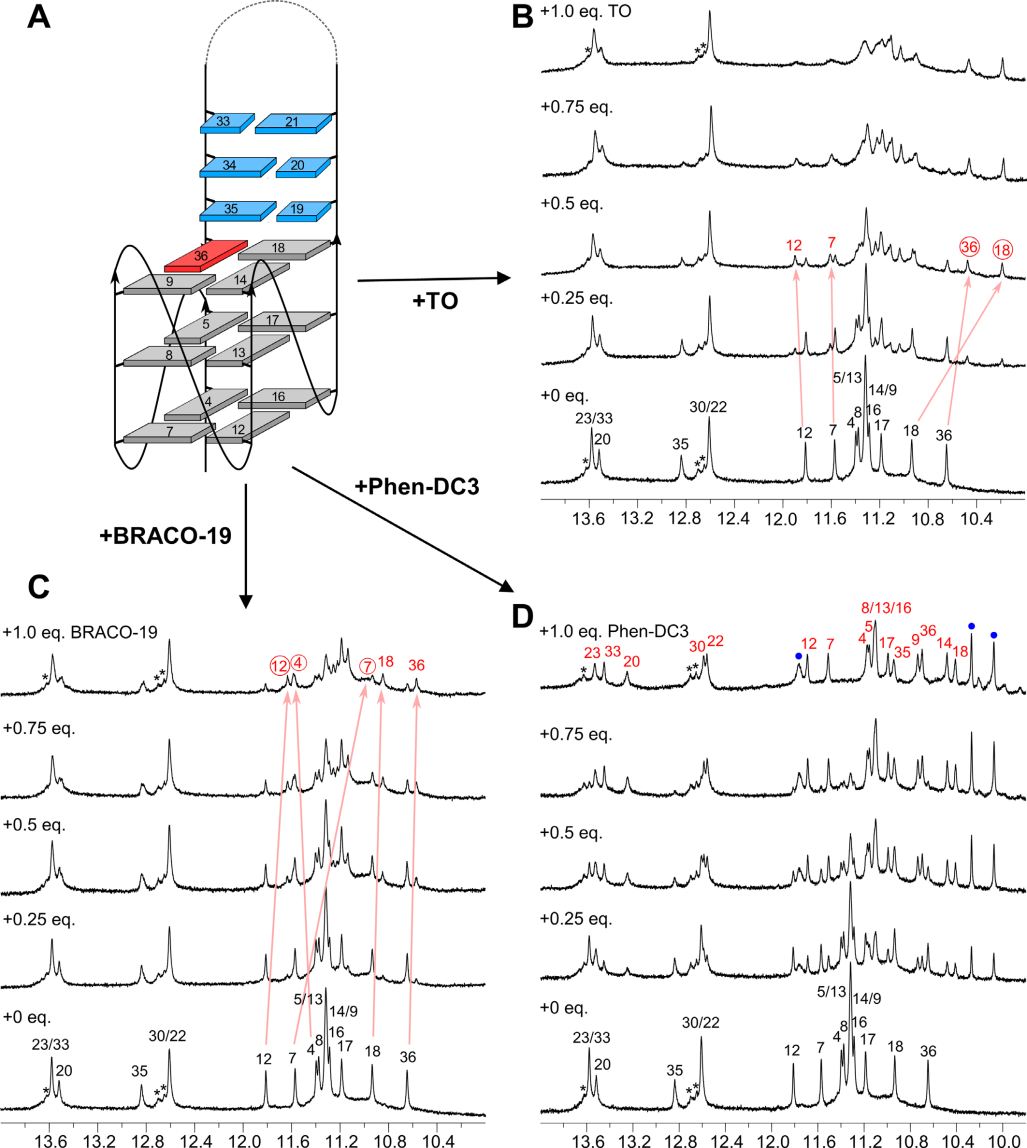
Topology with residue numbers of the *QD3-sbl* hybrid (**A**) and its titration with TO (**B**), BRACO-19 (**C**), and Phen-DC3 (**D**). Spectra show Watson–Crick and G Hoogsteen imino resonances with full and partial assignments of the free *QD3-sbl* (black) and the *QD3-sbl*–ligand complex (red). Phen-DC3 protons in (D) are marked by blue dots. Representative chemical shift changes of assigned resonances upon TO and BRACO-19 addition are traced by arrows with resonances experiencing more significant shifts circled. Asterisks indicate resonances in the duplex region of a minor species. Spectra were acquired in 20 mM potassium phosphate buffer, 100 mM KCl, pH 7.0, at 30°C with a *QD3-sbl* concentration of 0.5 mM.

In the following, preferred binding sites were probed by NMR titrations of the ligands to *QD3-sbl*. Analysis of 1D and 2D NOESY experiments on the latter at 120 mM K^+^ confirmed its folding into a hybrid structure with the duplex domain coaxially stacked onto the quadruplex 3′-outer tetrad in analogy to the conformation recently reported for *QD3-sbl* under low-salt conditions (PDB ID: 7PNE) (25). For full resonance assignments and a compilation of chemical shift data see Supplementary Figure S4 and Supplementary Table S4. It should be noted that *QD3-sbl* exhibits additional non-identified signals of very low intensity within the Watson–Crick imino proton spectral region (Figure 4). A putative coexisting minor species may result from different arrangements of the TTT loop of the duplex hairpin closed by a neighboring AT base pair. In fact, with the hairpin loop remote from the G-core there is no observable impact on the G4 domain nor any shift or change of these weak resonances when binding the ligands (see below).

Adding thiazole orange to *QD3-sbl*, a new set of G imino signals emerges, reaching equal intensity to corresponding resonances of the free hybrid at an 0.5:1 molar ratio (Figure 4B). Clearly, such a behavior suggests binding of the ligand at a distinct binding site. However, imino resonances start to broaden after addition of > 0.5 equivalents of TO in line with the occupation of additional binding sites to form different complexes. Although linebroadening effects associated with the presence of more than a single complex prevented a more detailed structural evaluation of a 1:1 ligand-DNA solution, ROESY experiments on the 0.5:1 mixture revealed various imino proton exchange crosspeaks between free and complexed *QD3-sbl* (Supplementary Figure S5). Based on prior resonance assignments for the free Q–D hybrid, these exchange crosspeaks allowed for the unambiguous assignment of some imino protons in the formed complex. Conspicuously, most significant chemical shift changes were detected for G iminos within the 3′-tetrad such as G18 and G36 located at the Q–D interface. On the other hand, only minor shifts were observed for imino resonances at the 5′-tetrad. These results identify the Q–D junction as hotspot for initial TO binding.

Likewise, addition of BRACO-19 to *QD3-sbl* resulted in the appearance of new G imino resonances, increasing in intensity up to a 1:1 molar ratio (Figure 4C). ROESY experiments on the latter mixture showed various imino exchange crosspeaks, enabling partial imino resonance assignments for a major complex in equilibrium with the free Q–D hybrid (Supplementary Figure S6). Chemical shift perturbations differ significantly from those found upon TO binding with most shifted imino resonances located within the fully exposed 5′-tetrad and hardly affected G iminos at the Q–D interface. However, BRACO-19 seems to display a more complex binding behavior towards *QD3-sbl*, evading a simple description based on a 1:1 high-affinity binding at a single site. Thus, assuming strong binding it is puzzling to observe remaining signals attributable to the free hybrid even after the addition of one equivalent of ligand. Possible reasons such as cooperativity effects in binding or additional groove binding with minimal impact on G-tetrad or duplex imino protons could not be explored in more detail due to limitations in the NMR spectral analysis set by the heterogenous mixture with severe signal overlap. Importantly, however, in the absence of characteristic chemical shift perturbations a preferred binding and intercalation of BRACO-19 at the Q–D junction must be excluded as already suggested by the ITC data.

Titration of Phen-DC3 to *QD3-sbl* resulted in the appearance and gradual increase of a new set of signals for a ligand-DNA complex in slow exchange with the free G4 hybrid (Figure 4D). Also, after the addition of one equivalent of ligand only a single species with well resolved imino signals emerged. Spectra of the 1:1 complex attest to the strong binding of the ligand at a specific binding site on the G4 hybrid, amenable to a more detailed structural analysis.

### NMR high-resolution structure of the Phen–DC3 complex

When changing the high-salt buffer (120 mM K^+^) to a low-salt buffer (10 mM K^+^), proton resonances of the *QD3-sbl*–Phen-DC3 1:1 complex experienced some minor shifts whereas crosspeak patterns in the 2D NMR spectra remained virtually unaffected. Therefore, a following spectral analysis was performed on 2D NMR spectra acquired in a low-salt buffer to benefit from a better spectral quality. Established strategies making use of ^1^H–^13^C HSQC, DQF-COSY and NOESY spectra with different mixing times allowed for complete resonance assignments of the *QD3-sbl*–Phen-DC3 complex (for details see the Supplementary Information, Figures S7–S9, and Tables S5 and S6).

In general, only small chemical shift changes were observed for the Watson–Crick imino protons upon ligand addition. The interfacial G35 imino represents a striking exception, being significantly upfield-shifted in the complex by nearly 2 ppm to resonate within the Hoogsteen imino proton spectral region (Supplementary Figure S10). For the G-core, most noticeable upfield shifts were experienced by G iminos within the 3′-tetrad at the Q–D interface except for G36 filling the vacant position and showing only small chemical shift perturbations. Overall, a total of 63 intermolecular NOE contacts were identified between ligand protons and various exchangeable, aromatic, and anomeric DNA protons (Supplementary Figures S7 and S9). Contacts were observed between protons in the phenanthroline moiety and protons of G36 and G18 residues of the 3′-outer G-tetrad as well as C19 and G35 of the interfacial base pair. This clearly points to intercalative binding of the phenanthroline at the junction. Additional contacts position the quinoline side arms above the exposed part of the 3′-outer G-quartet to cover the whole tetrad face. Apparently, such a binding mode at the junction deviates from a previously proposed complex structure with Phen-DC3 stacked onto the freely accessible tetrad opposite to the junction of a corresponding Q–D hybrid (21). However, some non-assigned intermolecular contacts of resonances to protons in the 5′-tetrad observed here under high-salt conditions may point to some competing ligand binding, albeit with much lower affinity (not shown).

**Figure 5.**
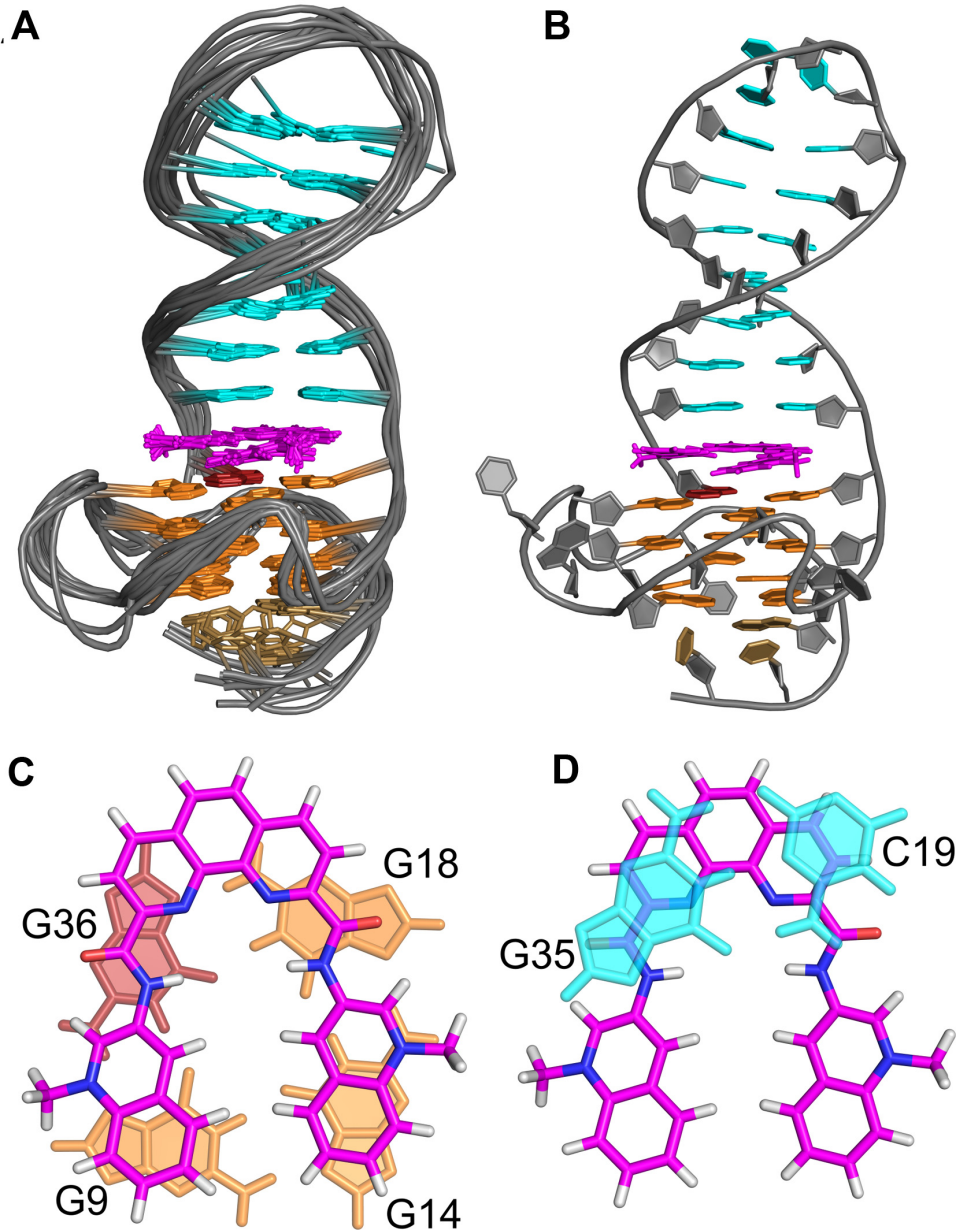
(**A**) Superposition of ten lowest-energy structures and (**B**) representative structure of *QD3-sbl* complexed with Phen-DC3. (**C**) Stacking of Phen-DC3 onto the 3′-tetrad and (**D**) below the C19·G35 interfacial base pair. In (A), residues of the propeller and duplex hairpin loop have been omitted for clarity.

Structure calculations employing NMR-derived distance and torsion angle restraints yielded a well-defined structural ensemble (for structural statistics see Table 2). The ligand intercalates at the Q–D interface with the phenanthroline located below the C19·G35 interfacial base pair and the two quinoline side arms covering the two exposed G bases of the 3′-tetrad at the junction (Figure 5). There are significant stacking interactions of the ligand with the GC base pair of the duplex domain and guanines of the 3′-tetrad. Based on the ligand orientation, the negligible shift of the *syn*-G36 imino resonance when compared to the other significantly upfield-shifted G iminos within the 3′-tetrad can be attributed to pronounced stacking interactions with G35 in the free hybrid (PDB: 7PNE) combined with its location below the Phen-DC3 amide linkage, associated with less shielding ring current effects in the complex (Figure 5C). On the other hand, there are no obvious electrostatic interactions between the positively charged *N*-methylated quinolines and the DNA hybrid, indicating stacking interactions to be a major driving force for the observed binding mode.

**Table 2.** NMR restraints and structural statistics for calculated structures

Restraints	*QD3-sbl*–Phen-DC3	*QD2-l*–PIQ-4m
NOE distance restraints		
intraresidual	150	114
interresidual	231	175
exchangeable	75	41
intramolecular ligand	0	6
intermolecular ligand–DNA	63	41
other restraints		
hydrogen bonds	82	64
dihedral angles	68	53
planarity	3	2
structural statistics after refinement		
pairwise heavy atom RMSD value (Å)		
all residues	2.21 ± 0.39	1.37 ± 0.47
G-tetrad core	0.98 ± 0.2	0.44 ± 0.1
Q–D interface with ligand	0.66 ± 0.15	0.76 ± 0.23
NOE violations		
number of NOE violation (>0.2 Å)	0.4 ± 0.5	0
maximum violation	0.263 Å	0.09 Å
mean NOE violation	0.002 ± 0.0005	0.001 ± 0.0003
deviations from idealized geometry		
bond lengths (Å)	0.01 ± 0.0001	0.01 ± 0.0001
bond angles (degree)	2.25 ± 0.03	2.34 ± 0.02

### Phen-DC3 changes binding sites upon removal of the duplex stem loop

The *Q3-sbl* sequence employed for the ITC experiments was designed for effectively blocking ligand access to the 3′-tetrad. However, keeping the lateral snapback loop of the original *QD3-sbl* design to only replace the duplex stem loop by a 3-nucleotide non-base-paired TGT lateral loop as for the *TBA* G4 will give additional insight into the importance of regular Q–D junctions for selective Phen-DC3 binding. Folding of the corresponding sequence *Q3*-*sbl2* was again confirmed by conventional strategies for the assignment of quadruplex structures (for details see the Supporting Information, Supplementary Figure S11 and Supplementary Table S7). Thus, NOESY experiments supported by ^1^H–^13^C HSQC spectra demonstrated folding of *Q3-sbl2* into a parallel three-layered G4 with exclusive homopolar tetrad stackings and a first broken G-column. In analogy to the *QD3-sbl* hybrid structure, the empty tetrad position as a consequence of the truncated first G-tract is filled by the 3′-G of the snapback loop in a *syn* conformation. The TGT lateral snapback loop seems to effectively cap the 3′-tetrad as suggested by the underlying G22 amino proton shown to be protected from fast solvent exchange.

**Figure 6.**
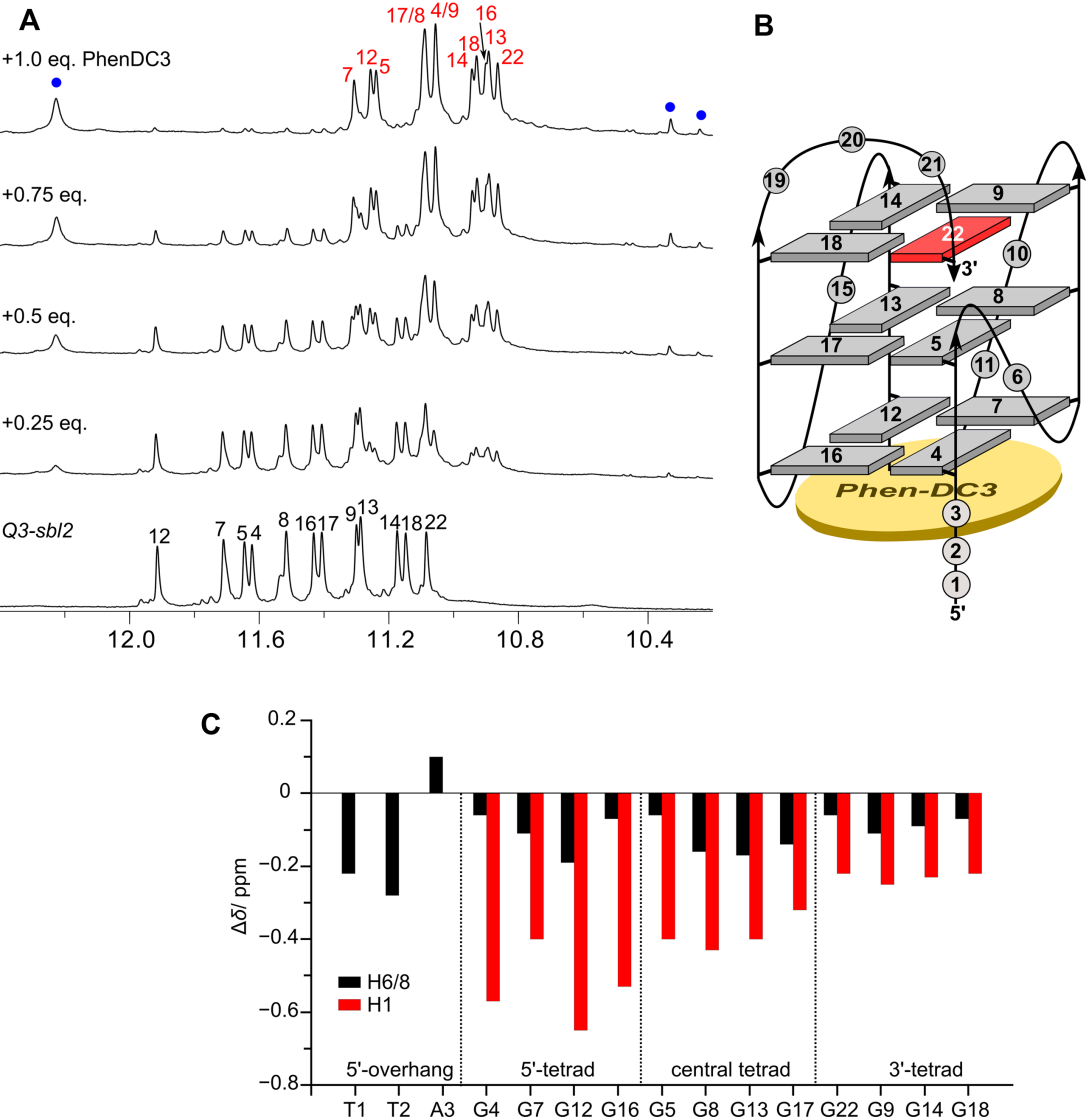
(**A**) Imino proton spectral region of *Q3*-*sbl2* (0.75 mM) upon titrating Phen-DC3 up to a 1:1 molar ratio in 10 mM potassium phosphate buffer, pH 7.0, at 30°C; assignments of the free *Q3-sbl2* and the *Q3-sbl2*–ligand complex are shown in black and red; Phen-DC3 resonances are marked by blue dots. (**B**) Schematic representation of *Q3*-*sbl2* with residue numbers showing the preferred binding site of Phen-DC3 at the 5′-outer tetrad. (**C**) Imino and H6/H8 chemical shift perturbations after binding Phen-DC3 to *Q3-sbl2*. Complete resonance assignments of the free and complexed DNA and a compilation of chemical shifts are given in Supplementary Figures S11–S13 and in Tables S7 and S8, respectively.

Initially, titration of Phen-DC3 to *Q3-sbl2* was followed by CD spectroscopy (Supplementary Figure S12A). In analogy to its addition to the *QD3-sbl* hybrid, the parallel CD signature of the G4 is preserved even with ligand in excess and a negative ICD at the ligand absorption develops, albeit only with > 1 equivalent of added ligand. In the following, NMR spectra acquired upon the addition of Phen-DC3 to *Q3-sbl2* showed the emergence of a new set of Hoogsteen imino resonances, fully replacing imino signals of the free G4 at a 1:1 molar ratio (Figure 6A). Full assignment of the 1:1 *Q3-sbl2*–Phen-DC3 complex reveals that most significant chemical shift changes are clearly experienced by imino protons of the 5′-tetrad with iminos of the 3′-tetrad least affected (Figure 6C). Also, NOE contacts involving snapback loop residues are conserved upon ligand binding (Supplementary Figure S12). These data demonstrate that the short non-base-paired lateral snapback loop effectively prevents stacking of Phen-DC3 onto the 3′-tetrad but rather redirects the ligand to the exposed 5′-tetrad at the opposite G4 face. Unlike Phen-DC3 binding to the *QD3-sbl* hybrid but in analogy to a complex with Phen-DC3 stacked on an outer tetrad of a parallel G4 (40), exchange crosspeaks were observed for pairs of protons in the symmetry-related quinoline units of bound Phen-DC3 (Supplementary Figure S13). Such an exchange of the chemical environment for the two quinoline side arms requires the stacked ligand to flip and a corresponding flipping motion is expected to be significantly restricted upon Phen-DC3 intercalation at a Q–D junction in line with present observations.

Taken together, Phen-DC3 known to be a universal G4 binder is shown for the first time to favor a Q–D junction with a coaxially stacked base pair at the interface as most affine binding site over stacking on an exposed outer tetrad. An intercalative binding mode at the junction requires the formation of an intercalation cavity with unwinding and tetrad unstacking of the interfacial base pair. Obviously, these energetically unfavorable processes are overcompensated by the considerable stacking interactions of the bound Phen-DC3 ligand. A lateral-type coaxially stacked duplex domain seems a prerequisite for strong binding at the junction and a non-duplex lateral loop will rather prevent binding due to unfavorable steric and/or compromised stacking interactions.

### The phenyl substituent of PIQ-4m extends towards the center of the G-tetrad

ITC experiments have demonstrated an entropically more favored and also a more selective binding at Q–D junctions for the phenyl-substituted indoloquinoline PIQ-4m when compared to its close derivative SYUIQ-5. The latter bears a simple aliphatic aminoalkyl side chain that has recently been shown to be oriented towards the duplex minor groove upon its intercalation at the junction (25). To get a better understanding of observed selectivities, binding of PIQ-4m was additionally studied on the *QD2*-*l* hybrid by an NMR structural analysis. The two-layered antiparallel *QD2*-*l* was employed because of a similar affinity when compared to the parallel *QD3-sbl* hybrid but with the additional benefit of eliminating putative competition with binding at the face opposite the Q–D junction as shown by ITC (see above).

Initially, the topology of *QD2-l* under the present solution conditions was verified to match the structure reported previously for the same sequence (PDB 2M8Z) (17). Again, standard strategies were employed for full resonance assignments of the hybrid (for a detailed description of assignment strategies, spectra, and a chemical shift table see the Supporting Information, Supplementary Figure S14 and Supplementary Table S9). Of note, this antiparallel two-layered G4 hybrid exhibits the same G-tetrad polarity as *QD3-sbl* with respect to the interfacial CG base pair at the junction, making the Q–D interface of both hybrids highly similar.

**Figure 7.**
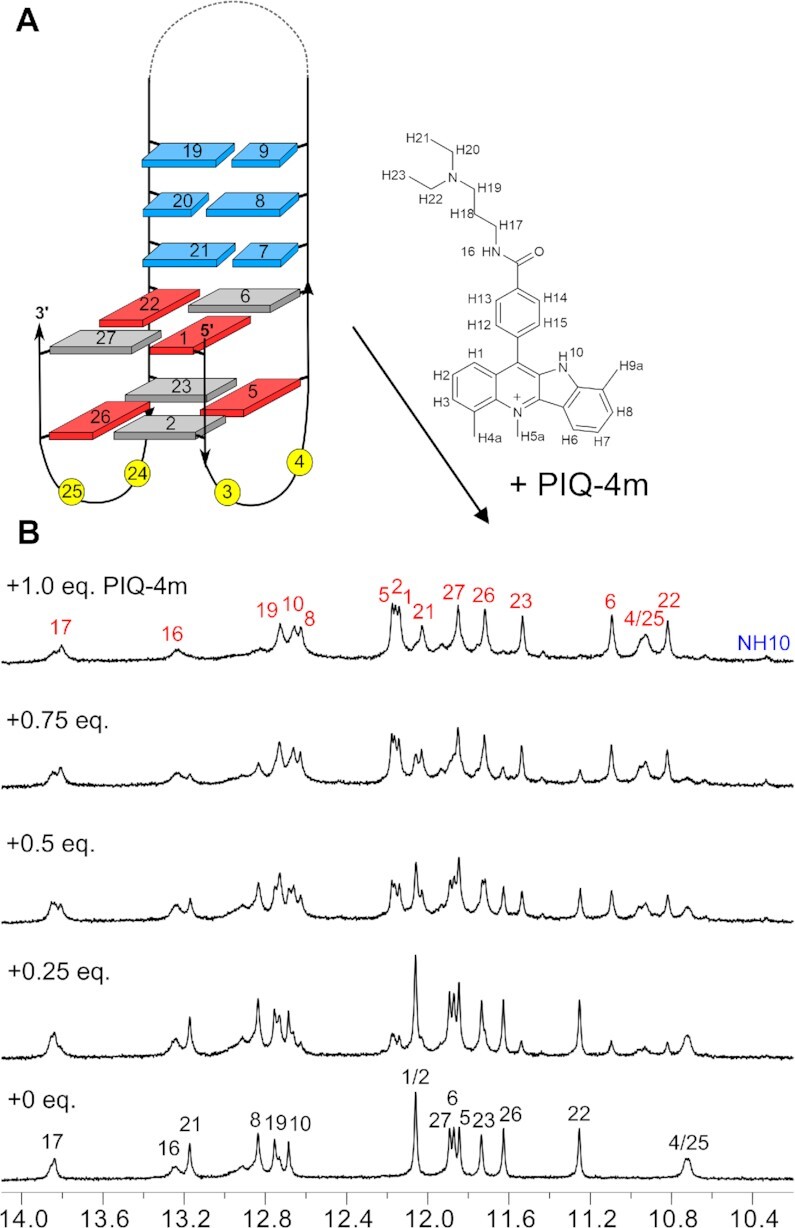
(**A**) Topology and chemical structure of the *QD2-l* hybrid and PIQ-4m indoloquinoline ligand with residue and atom labeling. (**B**) Imino proton spectral region of *QD2-l* (0.64 mM) upon titration with PIQ-4m up to a 1:1 molar ratio at 30°C in 20 mM potassium phosphate buffer, 100 mM KCl, pH 7.0. Imino proton resonances in free and ligand-bound *QD2-l* are labeled in black and red, respectively; NH10 represents a resonance from the ligand.

Gradual titration of PIQ-4m to *QD2-l* resulted in the appearance of new imino resonances and the complete disappearance of imino signals from the free hybrid after the addition of 1 equivalent of ligand (Figure 7). ROESY spectra of a 0.5:1 PIQ-4m–*QD2-l* mixture showed exchange crosspeaks between the free and complexed G4 hybrid. Most noticeable upfield shifts upon complex formation were observed for the imino proton of the interfacial C7·G21 base pair as well as for imino protons of G6 and G22 of the tetrad facing the duplex domain (Figure 7, Supplementary Figure S15). With a chemical shift changing by >1 ppm, the C7·G21 Watson–Crick imino signal shifts towards the typical chemical shift range of the G4 Hoogsteen imino protons in analogy to a corresponding shift when binding Phen-DC3 to the *QD3-sbl* hybrid. Full resonance assignments of the 1:1 complex by the analysis of NOESY, DQF-COSY, TOCSY and ^1^H–^13^C HSQC experiments confirmed these chemical shift perturbations and suggest an intercalative binding mode of the PIQ-4m ligand at the Q–D junction. For more detailed information on the full spectral assignment of the PIQ-4m–*QD2-l* complex with a compilation of proton chemical shifts see the Supporting Information (Supplementary Figures S15-S19, Tables S10 and S11).

A total of 41 intermolecular contacts between ligand and the G4 hybrid could be observed in NOESY spectra. Various contacts position the ligand indole ring system between G6 and C7 and the quinoline moiety between G21 and G22 bases (Supplementary Figure S18). Notably, H14/H15 protons of the PIQ-4m phenyl substituent at about 8 ppm exhibit strong NOE contacts to all four imino protons within the tetrad at the Q–D interface with some corresponding but weaker contacts also observed for H12/H13 protons located on the opposite side of the phenyl ring (Supplementary Figure S18G). Apparently, the para-substituted phenyl side chain points towards the center of the interfacial G4 tetrad rather than towards the exterior and duplex minor groove.

**Figure 8.**
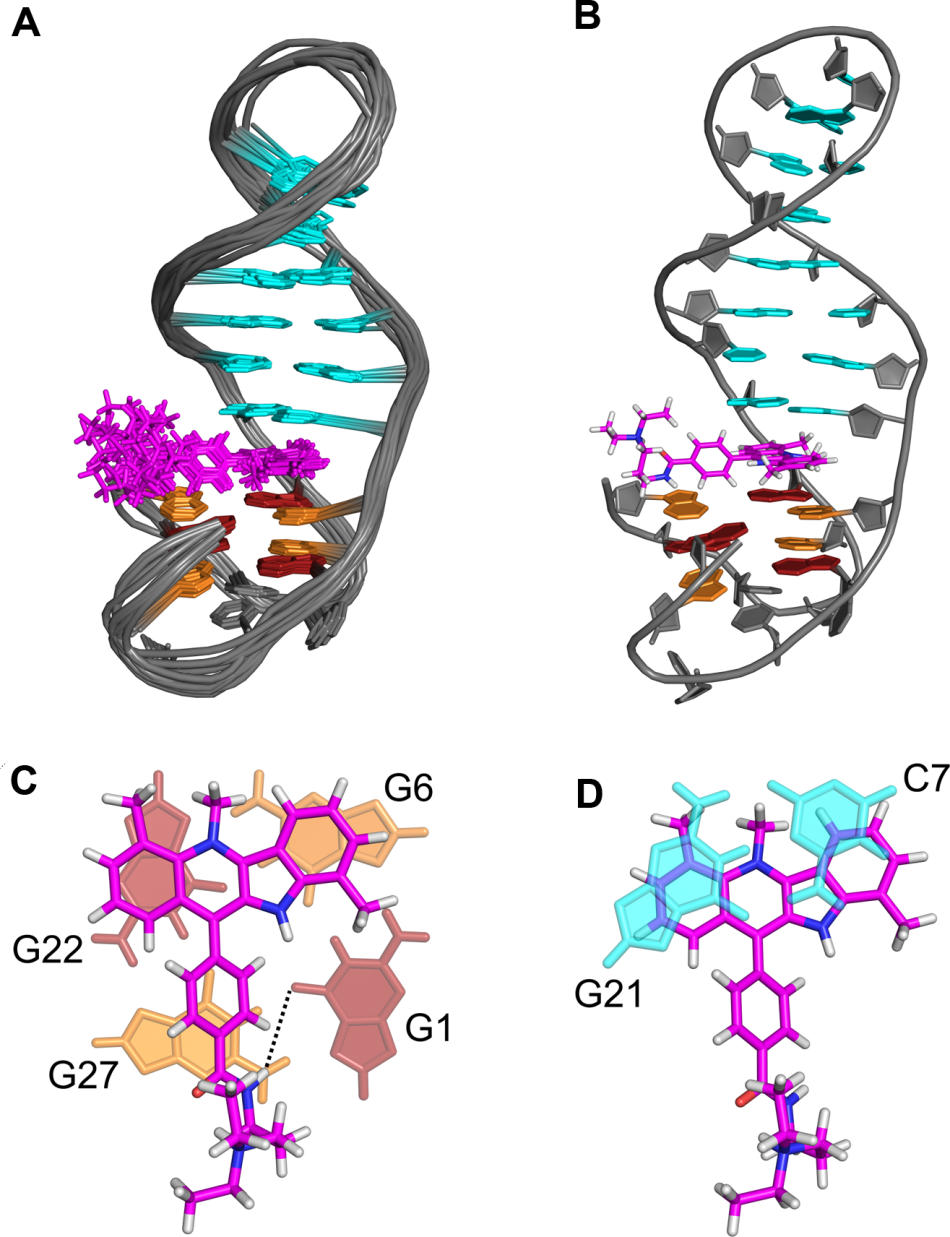
(**A**) Superposition of ten lowest-energy structures and (**B**) representative structure of *QD2-l* with bound PIQ-4m. (**C**) Detailed view of PIQ-4m stacked onto the G-tetrad and (**D**) below the C7·G21 base pair. A putative NHO hydrogen bond interaction between the PIQ amide and a guanine carbonyl is indicated by the dotted line in (C). In (A), residues of the duplex hairpin loop have been omitted for clarity.

Three-dimensional structures of the complex were calculated using NMR-derived experimental restraints. With average pairwise root mean square deviations of 1.4 Å for all atoms, 0.8 Å for the Q–D junction with intercalated ligand, and 0.4 Å for the G-core, there is a good convergence of final structures (Table 2). As suggested by intermolecular NOE contacts, the indoloquinoline intercalates between G-tetrad and base pair at the Q–D junction with the indole and quinoline subunits sandwiched between G6 and C7 and G21 and G22 residues, respectively (Figure 8). The PIQ-4m phenyl ring located above the center of the G-tetrad is tilted out of plane with respect to the indoloquinoline due to hydrogen-hydrogen steric repulsions in the 11-phenyl-indoloquinoline. Its orientation is well defined whereas the aminoalkyl side chain, amide-linked to the phenyl para-position and extending towards a G4 groove opposite the Q–D interface, seems rather flexible with no apparent long-lived electrostatic or hydrogen bond interactions with the G4. Conspicuously, NH16 of the amide functionality directly points towards the carbonyl oxygen of G1 in the structural ensemble with NHO angles of 157° ± 5°. Although this geometry suggests formation of a putative NHO hydrogen bond, H–O distances >3 Å do not support such an interaction to be of any major significance.

### Structural determinants of affinities and selectivities for Q–D junctions

Stacking interactions seem to be the main driving force for ligand intercalation at the Q–D interface. Thus, as shown for TO and also recently demonstrated for cryptolepine (25), planar intercalators even without any additional side arms recognize a Q–D junction as binding hotspot to outcompete other available DNA binding sites. A pathway for intercalation suggested initial outside binding of the ligand followed by gradual insertion into a wedge formed at the intercalation site (53). Energy barriers as a result of forming a binding cavity through helical unwinding and separation of stacked bases must be offset by the binding and stacking energies of the intercalator. The energetic penalty of base unstacking is expected to increase with more extended surface areas and the previously noted absence of any ligand intercalation at a putative Q–D junction of a particular Q–D hybrid design may derive from a formed base triad rather than a base pair stacked onto the G-tetrad platform (54). On the other hand, a recent study reported on simple aromatic hydrocarbon-based ligands to specifically recognize a Q–D junction by exclusively stacking onto the exposed area of the interfacial G-tetrad with a non-invaded Q–D junction (23). Such a non-intercalative binding mode may again be attributed to the inability of the aromatic hydrocarbons to overcome the energetic cost of forming an intercalation pocket at the junction.

**Figure 9.**
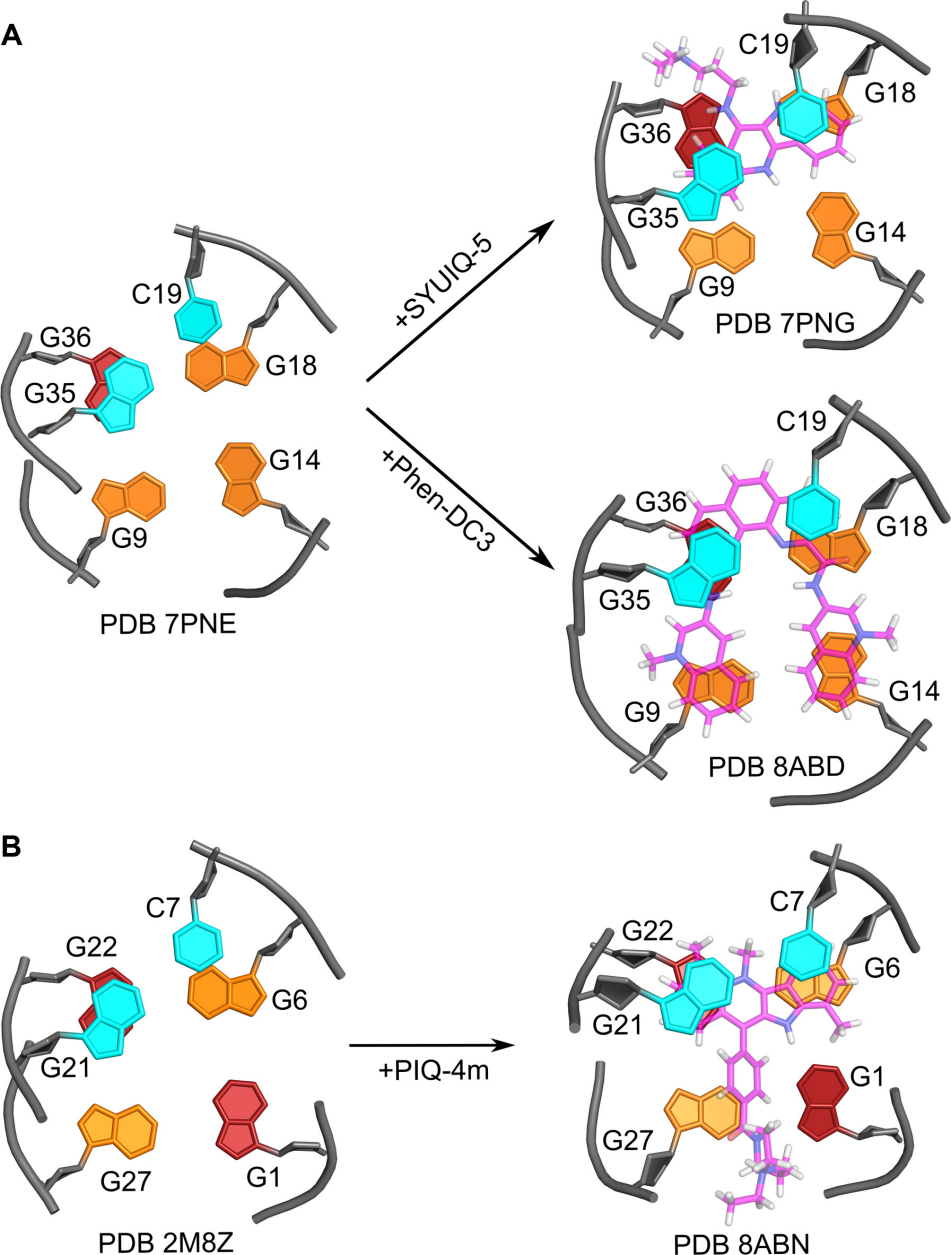
Q–D interface from high-resolution structures of free and ligand-bound Q–D hybrids. (**A**) Conformational changes of free *QD3-sbl* (PDB 7PNE) upon binding SYUIQ-5 (PDB 7PNG) (25) and Phen-DC3 (this study, PDB 8ABD). (**B**) Conformational change of free *QD2-l* (PDB 2M8Z) upon binding PIQ-4m (this study, PDB 8ABN).

A closer look at the Q–D interface upon ligand intercalation reveals conformational adjustments to optimize stacking interactions. Thus, inspection of available pairs of free and ligand-intercalated high-resolution structures of Q–D hybrids indicates a noticeable shift of the interfacial 5′-base of the duplex stem loop towards the tetrad for maximizing stacking interactions with the sandwiched ligand (Figure 9). Earlier studies on intercalation complexes with a B-type duplex or a dinucleotide base pair model have reported higher glycosidic torsion angles χ for the 3′-residues than for the 5′-residues of the intercalation site (55). In fact, the cytosine residue located at the 5′-end of the duplex stem loop, i.e. at the 3′-side of the junction experiences a significant increase of χ towards the *high-anti* range for complexes with both *QD3-sbl* and *QD2-l* (Supplementary Table S12). As a result, the pyrimidine nucleotide of the base pair at the Q–D junction adopts higher glycosidic torsion angles than the complementary purine nucleotide. This contrasts with the non-complexed hybrids and the general expectation of a higher propensity for purine residues to adopt glycosidic torsion angles in the *high-anti* range. However, the terminal *syn*-G located at the other 3′-end of the intercalation site shows no significant change in its glycosidic torsion angle as expected from its participation and fixation within the outer G-tetrad.

Attaching appropriate side arms to the planar aromatic core structure of G4 ligands is considered highly beneficial for binding affinity but also binding selectivity, allowing for additional stabilizing interactions in grooves or with loop regions. It is instructive to compare binding geometries at the Q–D junction for indoloquinolines PIQ-4m and SYUIQ-5, the latter determined only recently (25). Conspicuously, for intercalated SYUIQ-5 the convex side of the slightly crescent-shaped indoloquinoline is positioned towards the outer surface at the Q–D interface, allowing its flexible side chain to interact within the duplex minor groove. In striking contrast, it is the concave side of intercalated PIQ-4m that is directed towards the exterior, positioning the out-of-plane phenyl substituent in opposite direction at the duplex major groove side and above the exposed part of the tetrad. A corresponding alignment of phenyl-indoloquinoline derivatives has also been reported upon their intercalation at duplex-triplex junctions with the phenyl ring again oriented towards the duplex major groove (56,57). On the other hand, the natural alkaloid cryptolepine lacking any side chain for controlling the ligand alignment was previously shown to intercalate between CG base pairs with its convex side towards the duplex minor groove (58). Apparently, the twisted phenyl substituent disfavors such a ‘convex-out’ binding mode, expected to be preferred based on a slightly better geometric match of the indoloquinoline sandwiched between a Watson–Crick and a G-tetrad.

More specific interactions can be expected from the SYUIQ-5 side chain if inserted into the duplex minor groove. On the other hand, favorable hydrophobic effects may accompany the positioning of the phenyl substituent of PIQ-4m above the exposed surface area of the tetrad. Indeed, less exothermic but much more favorable entropic contributions are associated with PIQ-4m binding at the junction when compared to SYUIQ-5 (see Supplementary Table S1). Combining enthalpic and entropic contributions, both indoloquinolines bind with similarly high affinity to the Q–D junction, yet selectivities against duplex DNA significantly differ in favor of the PIQ-4m derivative (see Figure 2). Given its less favorable binding enthalpy generally associated with less specific interactions, such an increase in selective binding seems counterintuitive at first. However, side chain interactions in the duplex minor groove of a Q–D hybrid structure are also expected to promote binding to a free duplex if the geometry of the ligand polycyclic ring system also permits intercalation between Watson–Crick base pairs. Thus, the duplex association constant of SYUIQ-5 is higher by nearly two orders of magnitude when compared to PIQ-4m (Figure 2), raising doubts about the use of SYUIQ-5 as a G4 ligand in the presence of duplex DNA. Although not evident at first, PIQ-4m in fact shares similarities with Phen-DC3 in binding a Q–D junction. With the latter positioning its quinoline arms above the open side of the tetrad, both ligands lack additional interactions with the duplex minor groove but add stabilizing side chain contributions mainly through stacking, van der Waals, and/or hydrophobic interactions.

Side chains are often designed to carry additional positive charges through amine functionalities protonated at neutral pH. Although anticipated to form electrostatic or hydrogen bond interactions with the DNA backbone, the high flexibility of the PIQ-4m aminoalkyl arm did not allow observation of any specific contacts. To test the influence of charges on ligand binding to the Q–D hybrid, two additional PIQ derivatives PIQ-5m and PIQ-7m with a doubly charged *N*-aminopropyl-*N*-methyl-propyl and an uncharged ethoxypropyl arm were likewise employed for ITC studies in targeting the *QD2-l* receptor. As expected for ligands of the same family, thermograms strongly resemble corresponding heat profiles observed for PIQ-4m (Supplementary Figure S20). Excellent fits were obtained with a model of two binding sites of different affinity. In the absence of another freely accessible tetrad, these are assumed to be the Q–D junction and the duplex domain. Free energies *ΔG*° determined by curve fitting for both binding sites indicate a loss in initial high-affinity binding for PIQ-7m with its uncharged arm but no noticeable change when adding a second positively charged amino group in PIQ-5m (Table 3; for complete binding profiles see Supplementary Table S13). Consequently, the simply charged side chain of PIQ-4m seems to provide for additional, albeit short-lived and weak electrostatic or hydrogen bond interactions when binding at the junction but the addition of another positive charge hardly exerts any more stabilizing effect. On the other hand, binding to the duplex depends on the charge density of the side chains following the order PIQ-5m > PIQ-4m > PIQ-7m. These results suggest smaller free energy differences and thus less selectivity for PIQ-5m with its doubly charged substituent. Similar selectivities can be expected for PIQ-4m and PIQ-7m, albeit with a higher affinity for the singly charged PIQ-4m ligand. Although uncertainties for extracted parameters have to be taken into account, there is a clear trend in such charge-dependent affinity-selectivity relationships, largely matching previous findings from a comprehensive thermodynamic profiling on the binding of PIQ ligands to the exposed outer tetrad of a parallel *c-myc* quadruplex (29). Based on the above, caution has to be exercised when deciding on charged side arms for G4 recognition in the presence of additional competing nucleic acid structures.

**Table 3.** Standard free energies Δ*G*º for the binding of PIQ derivatives to *QD2-l* at 40°C^a^

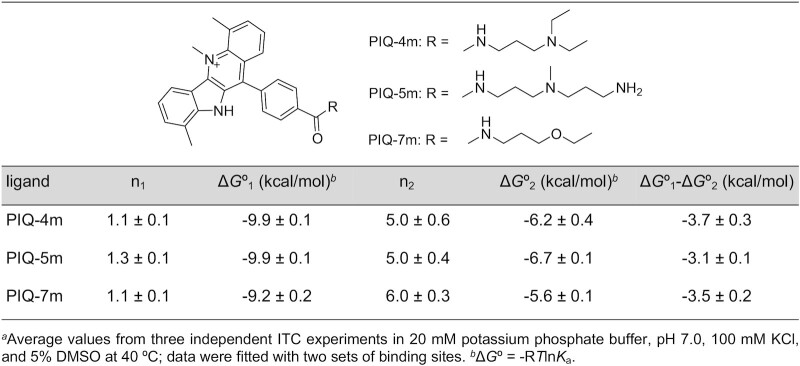

## CONCLUSIONS

Q–D junctions are unique structural motifs meeting increasing interest not only for their use in technological applications but also as targets for therapeutics due to their proposed frequent occurrence in genomic sequences. With only few reports on the specific recognition of such junctions through low molecular weight ligands to date, an extended set of detailed thermodynamic and structural data on various G4 binding compounds have been reported here. These demonstrate that Q–D junctions either featuring a hairpin-type snapback loop or a lateral duplex stem-loop with coaxial orientation of duplex and G4 helices represent no structural peculiarities calling for a novel type of Q–D ligands but are in fact superior binding sites for most G4 ligands usually found to stack on outer G-tetrads. Consequently, the targeting of G4 structures will benefit from higher affinities in the presence of such Q–D junctions due to enhanced stacking interactions upon ligand intercalation.

On the other hand, care must be exercised when following the obvious approach of designing Q–D ligands by combining G4 and duplex binding subunits for optimizing affinities and also selectivities. As suggested by the present studies, moieties designed for duplex minor groove binding, e.g. side chains attached to a planar aromatic core structure, may be detrimental to selectivities against duplex DNA due to an associated strong promotion of duplex binding. To confer more selectivity in the presence of excess double-helical nucleic acids, a strategy is proposed that directs additional interactions towards the G4 core away from the duplex minor groove in contrast to more common perceptions. This will require an extension of the intercalating ligand through side arms to favorably align and interact with the exposed area of the interfacial tetrad. Clearly, such design principles will strictly apply to polycyclic ligands with aromatic surface areas that reasonably match the base pair geometry to also allow for favorable duplex intercalation, as in fact seen for many typical G4 binding compounds. In contrast, macrocyclic or any other G4 ligands with extended surface areas as also represented by U-shaped Phen-DC3 are unable to efficiently intercalate between base pairs. Thus, whereas Phen-DC3 already favors a Q–D junction over any other G4 binding site, Q–D intercalation is expected here to additionally benefit from appropriate minor groove binding side chains, enhancing affinity without compromising selectivity. With studies encompassing different binding partners, the present results provide for valuable general guidelines to support the future design of potent ligands not only for selective Q–D recognition but also for G4 targeting in the presence of predominant duplex structures.

## DATA AVAILABILITY

The atomic coordinates and chemical shifts for *QD3-sbl* with bound Phen-DC3 (PDB ID: 8ABD, BMRB ID: 34740) and for *QD2-l* with bound PIQ-4m (PDB ID: 8ABN, BMRB ID: 34741) have been deposited in the Protein Data Bank.

## Supplementary Material

gkac1088_Supplemental_File
