# Supplementary material for: High-affinity binding at quadruplex–duplex junctions: rather the rule than the exception
Source: Nucleic Acids Res. 2022 Nov 23;50(20):11948–64. doi: 10.1093/nar/gkac1088 (PMC9723630; doi:10.1093/nar/gkac1088)
Supplement: gkac1088_Supplemental_File [file gkac1088_supplemental_file.pdf]

Supplementary Information
for

High-Affinity Binding at Quadruplex-Duplex Junctions: Rather the Rule than the Exception

Yoanes Maria Vianney and Klaus Weisz*

Institute of Biochemistry, Universität Greifswald, 17489 Greifswald, Germany

Contents

Experimental details S3

Table S1: Binding parameters of ligands from ITC titrations S4

Table S2. UV melting temperature of DNA constructs S5

Table S3: Binding parameters for PIQ-4m from ITC titrations S6

Figure S1: NMR imino proton spectral region of *QD2-l-2bp* S7

Figure S2: CD melting curves of *QD2-l* with and without ligands S7

Figure S3: CD spectra of *QD3-sbl* upon the addition of ligands S8

Figure S4: NOESY spectral regions of a *QD3-sbl* S9

Table S4: Compilation of ^{1}H and ^{13}C chemical shifts for free *QD3-sbl* S10

Figure S5: ROESY imino exchange crosspeaks for a *QD3-sbl* – TO complex S11

Figure S6: ROESY imino exchange crosspeaks for a *QD3-sbl* – BRACO-19 complex S12

Resonance assignments of the *QD3-sbl* – Phen-DC3 complex S13

Figure S7: ^{1}H-^{13}C HSQC and NOESY spectral regions of the *QD3-sbl* – Phen-DC3 complex S15

Figure S8: H1’-H2’ NOE and DQF-COSY crosspeaks of the *QD3-sbl* – Phen-DC3 complex S16

Figure S9: NOESY spectral regions of the *QD3-sbl* – Phen-DC3 complex S17

Table S5: Compilation of ^{1}H and ^{13}C chemical shifts in a *QD3-sbl* – Phen-DC3 complex S18

Table S6: Compilation of ^{1}H chemical shifts of Phen-DC3 bound to *QD3-sbl* S19

Figure S10: Chemical shift perturbations of *QD3-sbl* upon Phen-DC3 binding S19

Resonance assignments of *Q3-sbl2* without and with bound Phen-DC3 S20

Figure S11: ^{1}H-^{13}C HSQC and NOESY spectral regions of *Q3-sbl2* S21

Table S7: Compilation of ^{1}H and ^{13}C chemical shifts for free *Q3-sbl2* S22

Figure S12: ^{1}H-^{13}C HSQC and NOESY spectral regions of the *Q3-sbl2* – Phen-DC3 complex S23

Figure S13: NOESY and ROESY spectral regions of the *Q3-sbl2* – Phen-DC3 complex S24

Table S8: Compilation of ^{1}H and ^{13}C chemical shifts in a *Q3-sbl2* – Phen-DC3 complex S25

Resonance assignments of *QD2-l* without and with bound PIQ-4m S26

Figure S14: NOESY spectral regions of free *QD2-l* S28

Table S9: Compilation of ^{1}H chemical shifts for free *QD2-l* S29

Figure S15: 1D, ROESY and chemical shift perturbations of *QD2-l* upon PIQ-4m binding S30

Figure S16: NOESY and ^{1}H-^{13}C HSQC spectral regions of the *QD2-l* – PIQ-4m complex S31

Figure S17: H1’-H2’ NOE and DQF-COSY crosspeaks of the *QD2-l* – PIQ-4m complex S32

Figure S18: NOESY spectral regions of the *QD2-l* – PIQ-4m complex S33

Figure S19: DQF-COSY, TOCSY, and ROESY spectra of the *QD2-l* – PIQ-4m complex S34

Table S10: Compilation of ^{1}H and ^{13}C chemical shifts in a *QD2-l* – PIQ-4m complex S35

Table S11: Compilation of ^{1}H chemical shifts of PIQ-4m bound to *QD2-l* S36

Table S12: Glycosidic torsion angles for nucleotides at Q-D junctions S36

Figure S20: ITC thermograms upon the titration of PIQ derivatives to *QD2-l* S37

Table S13: Binding parameters for PIQ derivatives from ITC titrations S37

EXPERIMENTAL SECTION

NMR experiments

A WATERGATE W5 pulse scheme was employed for water suppression in one-dimensional (1D) and two-dimensional (2D) NOESY experiments on a 90% H_2O/10% D_2O phosphate buffer solution. For DQF-COSY, TOCSY, and ^1H-^{13}C HSQC experiments, a 3-9-19 W3 binomial-type sequence was used for solvent suppression. ^1H-^{13}C HSQC spectra were recorded with 4K x 500 data points, a 1 s recycle delay, and 7500 Hz in the F1 dimension to include aromatic C8/C6/C2 resonances of the nucleobases. TOCSY spectra with a DIPSI-2 isotropic mixing scheme and an 80 ms mixing time as well as DQF-COSY spectra were recorded with 4K x 500 data points. 2D NOESY (80, 150, and 300 ms mixing time) and EASY-ROESY spectra (80 ms mixing time, 50° spinlock angle) were acquired with 2K x 1K data points. For all 2D homonuclear experiments a 2 s recycle delay was used. Data were zero-filled to 4K x 1K data points and processed with squared sine-bell window functions except for time domain data of 1D experiments that were multiplied with an exponential function.

Restraints for structure calculations

For the structure calculations, distance restraints were assigned based on the crosspeak intensities in NOESY spectra. For non-exchangeable protons and intramolecular ligand contacts, these were set according to 2.9 ± 1.1 Å for strong crosspeaks, 4.0 ± 1.2 Å for crosspeaks of medium intensity, 5.5 ± 1.5 Å for weak crosspeaks, 6.0 ± 1.5 Å for very weak crosspeaks, and 5.0 ± 2.0 Å for ambiguous crosspeaks due to signal overlap. For labile protons, distances were restrained according to 2.9 ± 1.1 Å for strong crosspeaks, 4.0 ± 1.2 Å for crosspeaks of medium intensity, 5.0 ± 1.2 Å for weak crosspeaks, 6.0 ± 1.2 Å for very weak crosspeaks, and 5.0 ± 2.0 Å for ambiguous crosspeaks due to signal overlap. Intermolecular ligand-DNA contacts were set to 4.0 +1.5/-2.0 Å for strong crosspeaks, 5.5 +1.5/-2.0 Å for weak crosspeaks, 6.0 +1.5/-2.0 Å for very weak crosspeaks, and 5.0 ± 2.0 Å for ambiguous crosspeaks due to signal overlap. Torsion angles χ were either set to *anti* (170° – 310°) or *syn* (25° – 95°). Sugar pucker information was derived from DQF-COSY crossspeaks, whose in-phase/antiphase pattern is determined by corresponding coupling constants and enables differentiation between a *north* (pseudorotation angle of 0° – 36°) and *south* sugar pucker (pseudorotation angle of 144° – 180°) through the Karplus-relationship. Distance restraints for hydrogen bonds were added for the G-tetrads and all Watson-Crick base pairs and additional planarity restraints were added to the quadruplex core. Force constants for *in vacuo* simulations were set to 40 and 50 $kcal \cdot mol^{-1} \cdot Å^{-2}$ for NOE-based distance and hydrogen bond restraints, 200 $kcal \cdot mol^{-1} \cdot rad^{-2}$ for glycosidic torsion angle and sugar pucker restraints, and 30 $kcal \cdot mol^{-1} \cdot Å^{-2}$ for G-tetrad planarity restraints. Additional chirality restraints with a restraint energy of 10 $kcal \cdot mol^{-1} \cdot rad^{-2}$ were added for calculations of the *QD3-sbl* - Phen-DC3 complex. For subsequent simulations in explicit water, only NOE-based distance and hydrogen bond restraints with restraint energies of 15 and 25 $kcal \cdot mol^{-1} \cdot Å^{-2}$ were employed.

Table S1. ITC-derived binding parameters of quadruplex ligands[a]

PIQ-4m titrated to	n	K_a (M^{-1})	ΔH^o (kcal/mol)	$-T\Delta S^o$ (kcal/mol)[b]
QD3-sbl	1.2 ± 0.1	$1.3\cdot10^7 \pm 2.9\cdot10^6$	-9.8 ± 0.1	-0.4 ± 0.2
	7.3 ± 0.2	$7.3\cdot10^3 \pm 2.5\cdot10^2$	-8.8 ± 0.2	3.2 ± 0.2
Q3-sbl	1.3 ± 0.1	$3.2\cdot10^6 \pm 1.4\cdot10^6$	-4.9 ± 0.3	-4.4 ± 0.5
	4.7 ± 0.9	$8.5\cdot10^4 \pm 1.4\cdot10^4$	-1.1 ± 0.4	-6.0 ± 0.3
D3-HP	1.8 ± 0.4	$1.6\cdot10^4 \pm 2.2\cdot10^3$	-6.1 ± 0.1[c]	0.1 ± 0.1[c]
SYUIQ-5 titrated to				
QD3-sbl	0.9 ± 0.1	$1.1\cdot10^7 \pm 3.3\cdot10^6$	-14.1 ± 0.2	4.0 ± 0.3
	5.7 ± 0.1	$9.2\cdot10^4 \pm 1.3\cdot10^4$	-11.8 ± 0.1	4.7 ± 0.2
Q3-sbl	1.0 ± 0.1	$2.1\cdot10^6 \pm 6.1\cdot10^5$	-12.2 ± 0.5	3.2 ± 0.4
	7.2 ± 1.2	$8.4\cdot10^4 \pm 4.2\cdot10^4$	-2.8 ± 0.4	-4.2 ± 0.7
D3-HP	1.8 ± 0.3	$1.3\cdot10^6 \pm 1.2\cdot10^5$	-11.5 ± 0.1[c]	2.7 ± 0.1[c]
	4.6 ± 0.7	$6.5\cdot10^4 \pm 5.0\cdot10^3$	-3.9 ± 1.1	-3.0 ± 1.1
TO titrated to				
QD3-sbl	1.8 ± 0.1	$1.1\cdot10^6 \pm 2.7\cdot10^4$	-13 ± 0.6	4.4 ± 0.6
	8.5 ± 0.5	$1.4\cdot10^4 \pm 2.9\cdot10^3$	-5.2 ± 0.3	-0.7 ± 0.3
Q3-sbl	0.8 ± 0.1	$1.7\cdot10^5 \pm 6.7\cdot10^4$	-7.9 ± 1.2	0.4 ± 1.5
D3-HP	1.5 ± 0.2	$4.1\cdot10^4 \pm 2.7\cdot10^3$	-11.7 ± 0.4	5.1 ± 0.3
NDI-DM titrated to				
QD3-sbl	3.0 ± 0.2	$1.2\cdot10^5 \pm 2.5\cdot10^4$	-12.5 ± 0.3	5.2 ± 0.4
Q3-sbl	1.1 ± 0.1	$9.5\cdot10^4 \pm 1.1\cdot10^4$	-8.7 ± 0.4	1.5 ± 0.4
D3-HP	1.8 ± 0.1	$7.2\cdot10^4 \pm 2.3\cdot10^4$	-8.7 ± 1.5	1.8 ± 1.7
BRACO-19 titrated to				
QD3-sbl	0.5 ± 0.1	$1.4\cdot10^7 \pm 6.1\cdot10^6$	-14.6 ± 0.9	4.4 ± 0.6
	5.6 ± 0.6	$8.2\cdot10^4 \pm 1.3\cdot10^4$	-12.9 ± 1.0	5.8 ± 0.9
Q3-sbl	1.2 ± 0.1	$3.0\cdot10^7 \pm 2.3\cdot10^6$	-10.3 ± 0.1	-0.3 ± 0.1
	4.2 ± 0.1	$1.0\cdot10^5 \pm 2.5\cdot10^3$	-4.0 ± 0.1	-3.2 ± 0.1
D3-HP	2.1 ± 0.1	$2.9\cdot10^6 \pm 6.9\cdot10^5$	-11.3 ± 0.2	2.1 ± 0.1
	3.9 ± 0.4	$1.4\cdot10^5 \pm 4.8\cdot10^4$	-2.2 ± 0.2	-5.1 ± 0.1

[a]Average values with root-mean-square deviations obtained from three independent measurements in 20 mM potassium phosphate buffer, pH 7.0, 100 mM KCl, and 5% DMSO at 40 ºC; data were fitted with one or two sets of binding sites with fit parameters of a second lower affinity binding site shown on a grey background. [b]$-T\Delta S^o = \Delta G^o - \Delta H^o$ with $\Delta G^o = -RT\ln K_a$. [c]Determined by an excess-site titration.

Table S2. UV melting temperature T_m of DNA constructs

name	T_m (°C)[a]	
	G4	duplex
QD3-sbl	63.3 ± 1.0	63.8 ± 0.9
Q3-sbl	>70[b]	---
D3-HP	---	55.9 ± 0.1
QD2-l	58.7 ± 0.2	62.7 ± 0.9
D2-HP	---	62.9 ± 0.4
QD2-l-2bp	52.0 ± 1.2	---[c]
TBA	51.0 ± 0.2	---
Q3-sbl2	n.d.[d]	---

[a]Determined by three independent UV melting experiments in ITC buffer. [b]No high-temperature baseline reached within temperature range. [c]No resolved duplex melting. [d]Not determined.

Table S3. ITC-derived binding parameters of PIQ-4m[a]

PIQ-4m titrated to	n	K_a (M^{-1})	ΔH^o (kcal/mol)	$-T\Delta S^o$ (kcal/mol)[b]
QD2-I	1.1 ± 0.1	$8.3 \cdot 10^6 \pm 1.2 \cdot 10^5$	-11.5 ± 0.3	1.6 ± 0.3
	5.0 ± 0.6	$2.5 \cdot 10^4 \pm 1.2 \cdot 10^4$	-6.5 ± 0.5	0.2 ± 0.8
D2-HP	2.9 ± 0.2	$4.5 \cdot 10^4 \pm 1.4 \cdot 10^4$	-6.6 ± 0.6	0.0 ± 0.8
QD2-I-2bp	1.1 ± 0.1	$7.1 \cdot 10^6 \pm 7.7 \cdot 10^4$	-12.6 ± 0.2	2.8 ± 0.2
	2.2 ± 0.5	$5.2 \cdot 10^4 \pm 1.0 \cdot 10^4$	-4.7 ± 0.8	-2.0 ± 0.9
TBA	3.2 ± 0.4	$2.6 \cdot 10^4 \pm 8.6 \cdot 10^3$	-8.1 ± 1.7	1.8 ± 1.9

[a]Average values with root-mean-square deviations obtained from three independent measurements in 20 mM potassium phosphate buffer, pH 7.0, 100 mM KCl, and 5% DMSO at 40 ºC; data were fitted with one or two sets of binding sites with fit parameters of a second lower affinity binding site shown on a grey background. [b]$-T\Delta S^o = \Delta G^o - \Delta H^o$ with $\Delta G^o = -RT\ln K_a$.

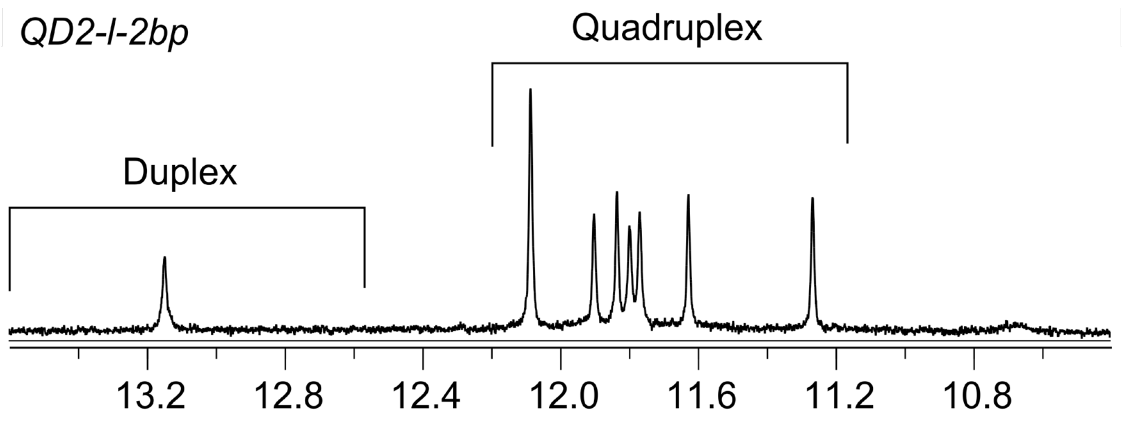

Figure S1. NMR imino proton spectral region of *QD2-I-2bp*. In addition to imino resonances of the quadruplex G-core, a GC Watson-Crick imino signal is also observed. Another potential Watson-Crick imino proton resonance seems to be broadened beyond detection through increased flexibility and associated fast solvent exchange under the present conditions. The spectrum was acquired with a strand concentration of 0.3 mM in 120 mM K^+ buffer, pH 7.0, at 40 °C.

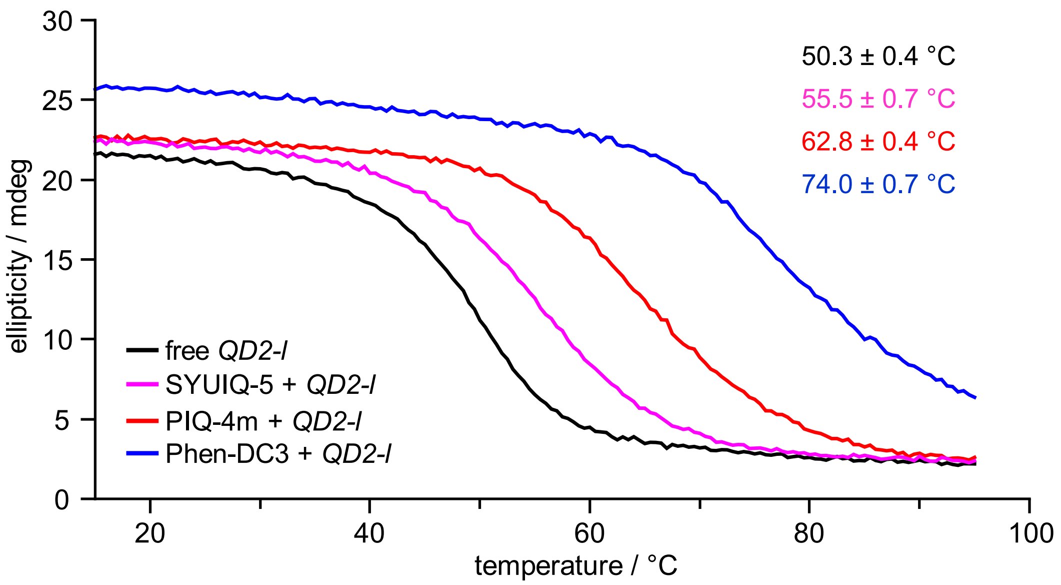

Figure S2. CD melting curves of *QD2-I* without (black) and with addition of 1 equivalent SYUIQ-5 (magenta), PIQ-4m (red), or Phen-DC3 (blue). Corresponding melting temperatures were determined from two independent experiments each in 10 mM potassium phosphate buffer, pH 7.0.

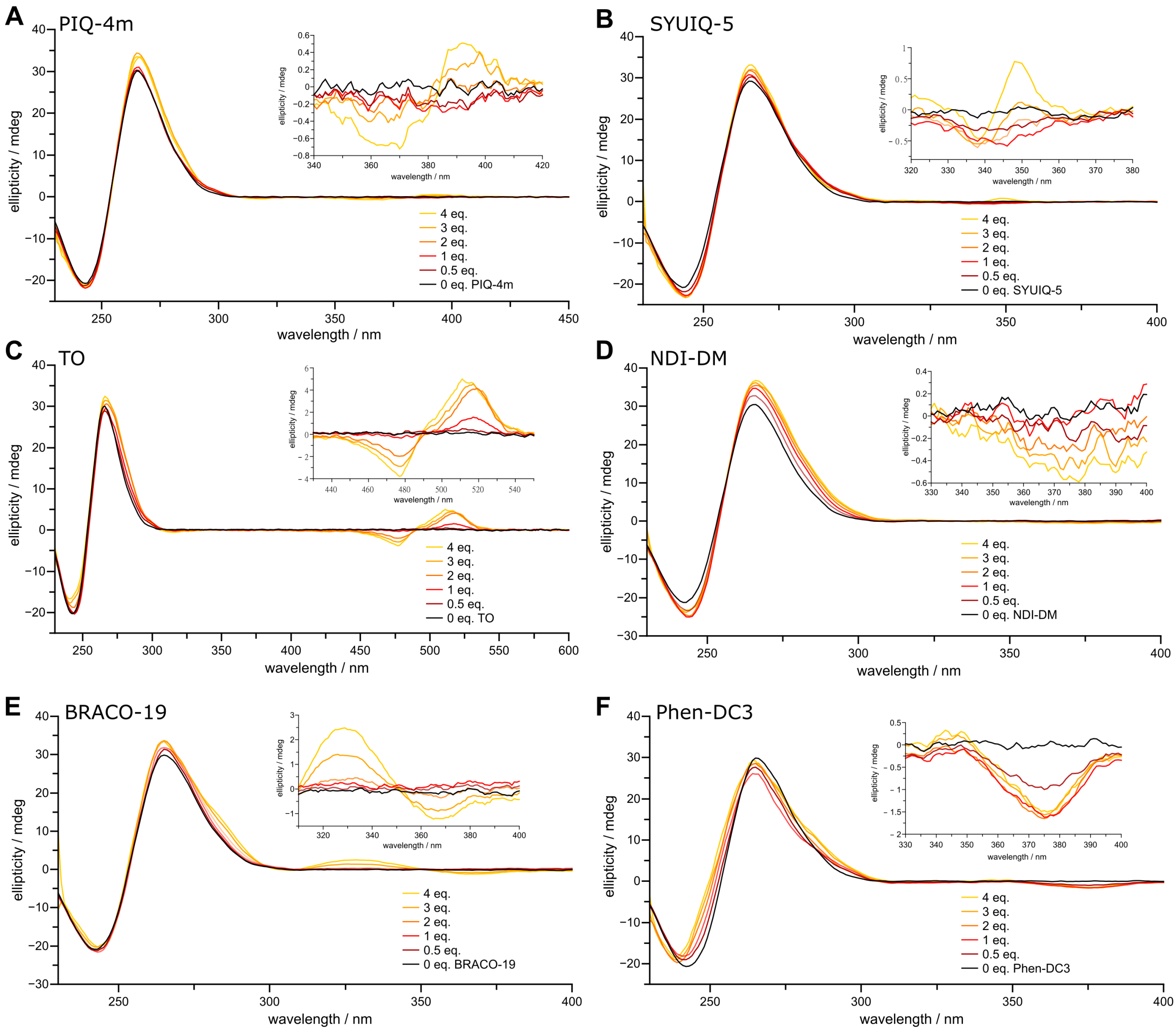

Figure S3. CD spectra of *QD3-sbl* upon addition of (A) PIQ-4m, (B) SYUIQ-5, (C) TO, (D) NDI-DM, (E) BRACO-19, and (F) Phen-DC3. Spectra were recorded at 30 ºC in 20 mM potassium phosphate buffer, 100 mM KCl, pH 7, and are blank-corrected.

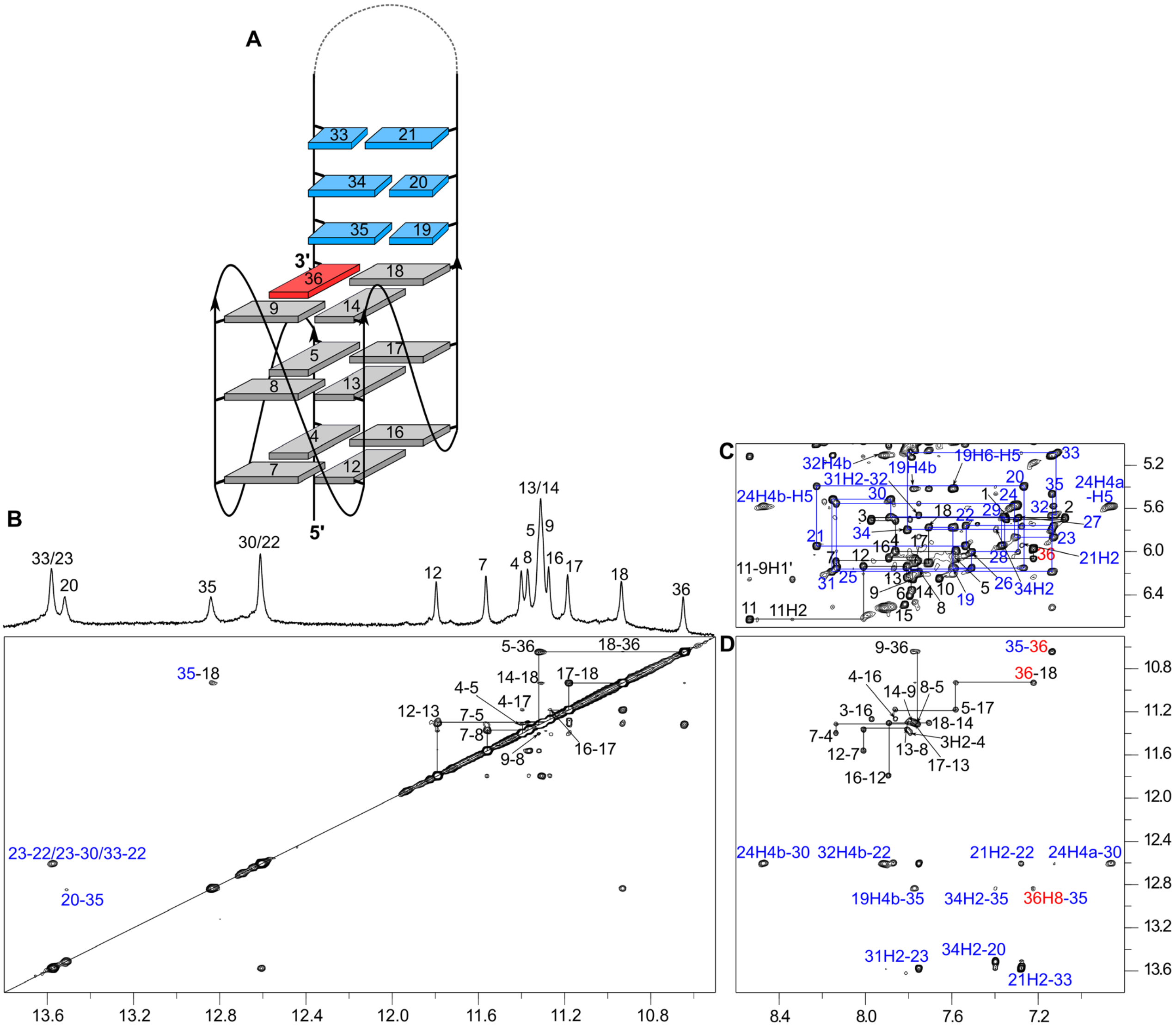

Figure S4. (A) Topology of *QD3-sbl* with residue numbers. (B) Imino(ω_2)-imino(ω_1) NOESY spectral region with corresponding 1D spectrum on top. (C) H6/H8(ω_2)-H1'(ω_1) NOESY spectral region; NOE connectivities and corresponding residue numbers within the quadruplex and duplex domains are given in black and blue color, respectively; *syn*-G36 is labeled in red. (D) H6/H8(ω_2)-imino(ω_1) NOESY spectral region. NOESY spectra (300 ms mixing time) of *QD3-sbl* (0.5 mM) were acquired in 20 mM potassium phosphate buffer, 100 mM KCl, pH 7.0, at 30 °C.

Note: Resonance assignments and the determination of a three-dimensional NMR structure of the same sequence has been published recently under different conditions (20 °C, 10 mM potassium phosphate buffer, pH 7.0; PDB 7PNE; BMRB 34664).[1] The present NMR analysis demonstrates that different buffer conditions as used here did result in some minor chemical shift changes but did not impact the formed topology.

1. Vianney, Y. M.; Weisz, K. Indoloquinoline Ligands Favor Intercalation at Quadruplex-Duplex Interfaces. *Chem. Eur. J.* **2022**, *28*. e202103718.

Table S4. ^{1}H and ^{13}C NMR chemical shifts δ for free *QD3-sbl*[a]

δ / ppm	H8/H6	H1/H3	H1'	H2'/H2"[b]	H3'	H5/H2/Me	C8/C6	C2
T1	7.35	n.d.	5.69	1.99/2.18	4.41	1.60	139.52	-
T2	7.08	n.d.	5.69	1.55/2.05	4.48	1.48	139.00	-
A3	7.97	-	5.71	2.55/2.62	4.82	7.80	141.82	154.95
G4	7.86	11.39	6.00	2.50/2.85	4.89	-	138.15	-
G5	7.58	11.31	6.06	2.36/2.61	4.98	-	137.97	-
T6	7.80	n.d.	6.41	2.38/2.61	4.96	1.93	140.04	-
G7	8.14	11.56	6.09	2.67/2.89	4.92	-	138.38	-
G8	7.76	11.37	6.20	2.57/2.93	4.99	-	138.09	-
G9	7.78	11.30	6.26	2.48/2.57	5.02	-	n.d.	-
T10	7.66	n.d.	6.25	2.24/2.46	4.75	1.93	140.11	-
A11	8.54	n.d.	6.63	2.92/3.01	5.12	8.34	143.07	155.73
G12	8.01	11.79	6.14	2.60/2.95	4.98	-	138.40	-
G13	7.81	11.31	6.24	2.63/3.04	5.02	-	138.29	-
G14	7.79	11.30	6.35	2.50/2.63	5.12	-	n.d.	-
T15	7.82	n.d.	6.49	2.46/2.66	5.06	1.96	139.90	-
G16	7.89	11.27	6.06	2.44/2.84	5.01	-	138.01	-
G17	7.77	11.18	6.10	2.62/2.91	5.06	-	138.07	-
G18	7.71	10.93	5.77	2.42/2.64	4.98	-	137.79	-
C19	7.59	-	6.15	2.03/2.42	4.88	5.42	n.d.	-
T20	7.27	13.51	5.39	2.05/2.26	4.82	1.60	139.58	-
A21	8.23	-	5.95	2.79/2.84	5.00	7.28	141.86	154.07
G22	7.54	12.61	5.76	2.39/2.62	4.75	-	137.19	-
T23	7.13	13.58	5.86	1.93/2.34	4.73	1.21	138.45	-
C24	7.31	-	5.56	1.74/2.18	4.73	5.58	143.54	-
A25	8.14	-	6.16	2.51/2.68	4.95	7.87	142.06	155.23
T26	7.51	n.d.	6.00	2.05/2.31	4.65	1.76	139.38	-
T27	7.29	n.d.	5.69	1.91/2.16	4.57	1.56	139.39	-
T28	7.37	n.d.	5.94	2.15/2.35	4.68	1.60	139.54	-
T29	7.36	n.d.	5.68	2.09/2.39	4.67	1.79	139.52	-
G30	7.89	12.61	5.52	2.64/2.73	4.95	-	138.35	-
A31	8.15	-	6.19	2.63/2.82	4.97	7.76	141.14	155.10
C32	7.14	-	5.67	1.71/2.22	4.58	5.11	n.d.	-
T33	7.12	13.58	5.09	1.79/1.92	4.57	1.40	139.61	-
A34	7.81	-	5.80	2.33/2.54	4.75	7.40	141.81	154.02
G35	7.14	12.84	5.46	2.20/2.65	4.83	-	136.65	-
G36	7.22	10.64	5.97	2.28/2.96	4.73	-	139.89	-

[a]At 30 ºC in 20 mM potassium phosphate buffer, 100 mM KCl, pH 7.0. [b]No stereospecific assignment.

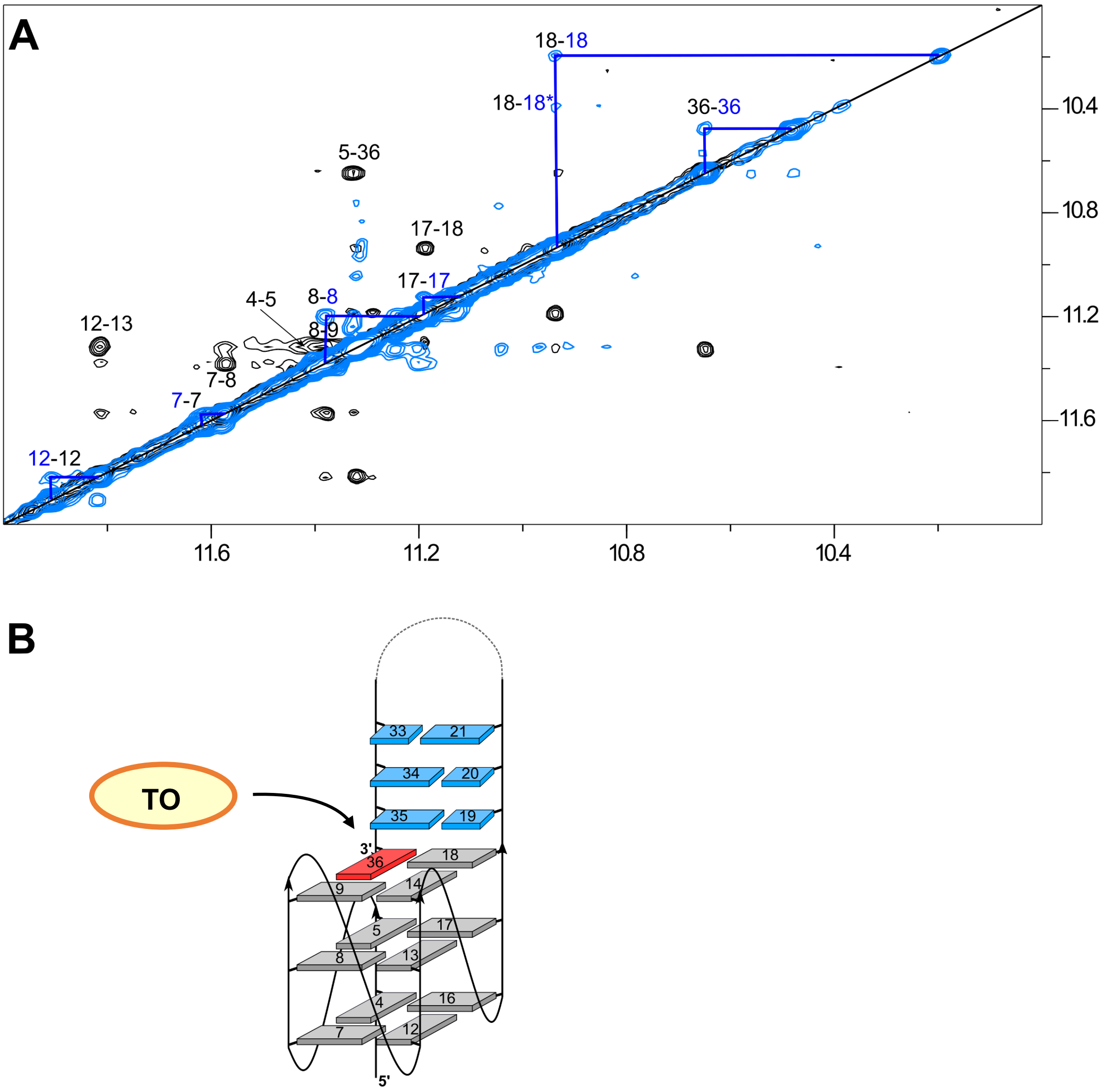

Figure S5. (A) Superposition of the imino(ω_2)-imino(ω_1) region in a NOESY spectrum (300 ms mixing time, black) on the free *QD3-sbl* hybrid and in a ROESY spectrum (blue) on a mixture of *QD3-sbl* and 0.5 equivalent of TO ligand. For simplicity, only positive exchange crosspeaks are shown in the ROESY spectrum with negative peaks suppressed. Blue lines connect imino protons exchanging between the free (labeled in black) and the complexed DNA (labeled in blue). Spectra were acquired in 20 mM potassium phosphate buffer, 100 mM KCl, pH 7.0, at 30 °C. (B) Schematic representation of high-affinity TO binding to *QD3-sbl*.

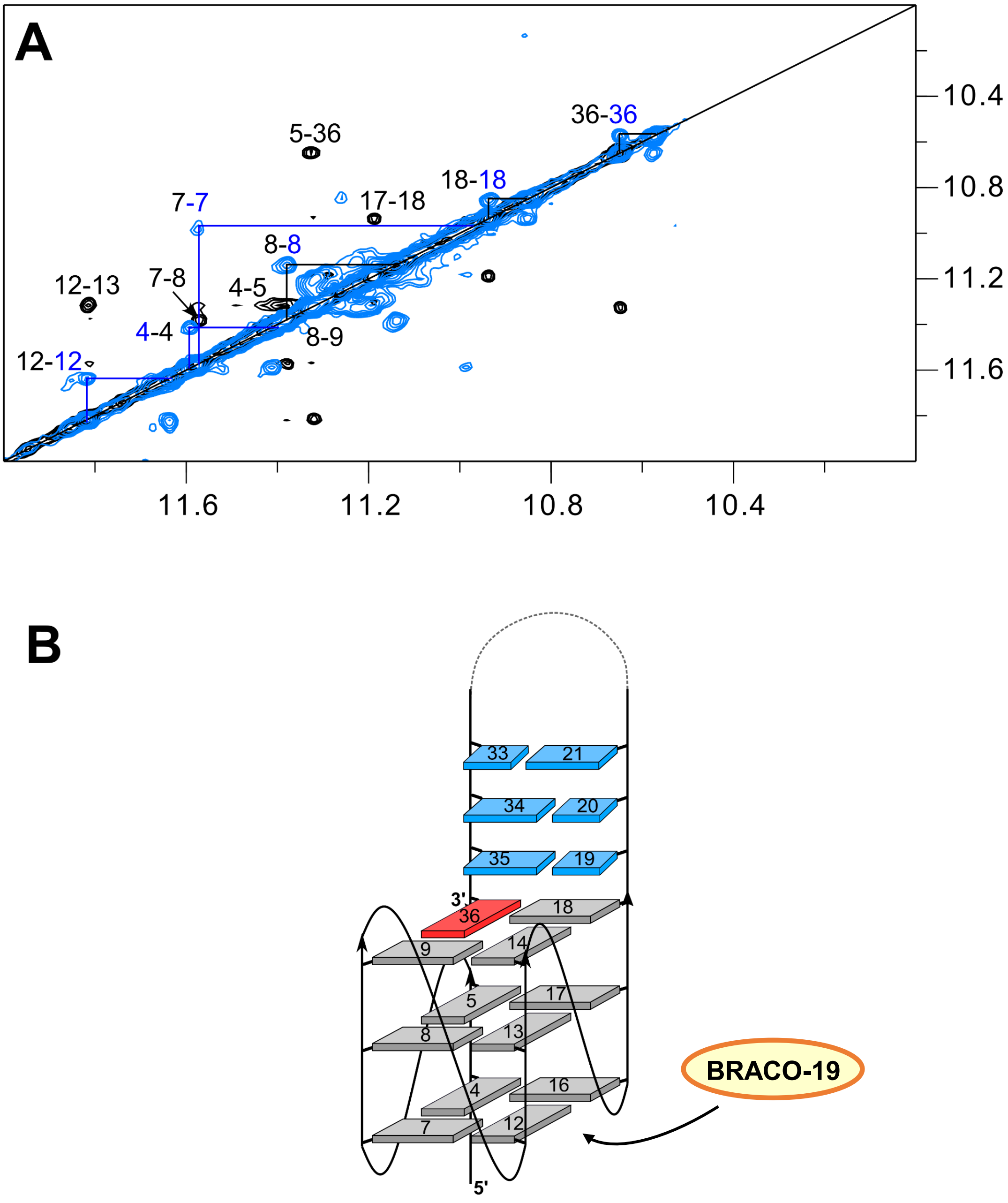

Figure S6. (A) Superposition of the imino(ω_2)-imino(ω_1) region in a NOESY spectrum (300 ms mixing time, black) on the free *QD3-sbl* hybrid and in a ROESY spectrum (blue) on a mixture of *QD3-sbl* and 1 equivalent of the BRACO-19 ligand. For simplicity, only positive exchange crosspeaks are shown in the ROESY spectrum with negative peaks suppressed. Blue lines connect imino protons exchanging between the free (labeled in black) and the complexed DNA (labeled in blue). Spectra were acquired in 20 mM potassium phosphate buffer, 100 mM KCl, pH 7.0, at 30 °C. (B) Schematic representation of high-affinity BRACO-19 binding to *QD3-sbl*.

Resonance assignments of a *QD3-sbl* complex with Phen-DC3

Continuous sugar-base NOE contacts can be followed from T1 through G5 and up to *syn*-G36 as well as along the other three G-columns with interruptions by the propeller-type loops (Figure S7B,C). The second and the third G-column can be differentiated by NOE contacts of A11 in the second propeller loop, showing sequential contacts to G12 but also additional contacts in particular through its H2 proton to G9 H1'. Sequential NOEs also connect all residues from C19 to G35 of the duplex stem. However, in contrast to the free *QD3-sbl*, NOE connectivities from the 3'-tetrad to the duplex are interrupted. The presence of G36 adopting a *syn* conformation is demonstrated by its strong H8-H1' intra-nucleotide crosspeak and its typical downfield-shifted G ^{13}C8 resonance observed in a ^{1}H-^{13}C HSQC experiment (Figure S7A). Stereospecific assignments of the H2'/H2" sugar protons are based on H1'-H2' crosspeak intensities in NOESY experiments acquired at short mixing times (Figure S8A). Sugar puckers were grouped into *south*-type and *north*-type conformations depending on the pattern and intensity of DQF-COSY H1'-H2' and H1'-H2" crosspeaks (Figure S8B). Most of the residues were found to be in a *south* conformation with some sugars remaining ambiguous and left unassigned. In contrast, T1 and G36 could be restrained to a *north* sugar conformation. Guanine imino (H1) protons of the G-core were assigned without specific isotope labeling by following the characteristic pattern of intra- and inter-tetrad H8-H1 contacts. Their assignment was confirmed by imino-imino connectivities typical for an all-homopolar stack of G-tetrads with polarities of the three tetrads when going from hydrogen bond donor to acceptor along G4→G7→G12→G16, G5→G8→G13→G17, and G36→G9→G14→G18 (Figure S9D,E). Contacts from H1' sugar protons of 5'-overhang residues to four imino resonances in the 5'-tetrad further confirmed their assignment (Figure S9F). Overall, the topology of *QD3-sbl* was retained after the addition of 1 equivalent Phen-DC3. Unlike free *QD3-sbl*, a prominent crosspeak correlating G35 H8 with G36 H1 is missing in the complex (Figure S9E). Imino protons of the Watson-Crick base pairs were identified through their crosspeaks with cytosine amino protons (H4a/H4b for non-hydrogen and hydrogen-bonded) and adenine H2 (Figure S9G,H). Notably, a contact between the hydrogen-bonded C19 amino with the considerably upfield-shifted G35 imino resonance in a NOESY experiment with 80 ms mixing time confirms an intact Watson-Crick CG base pair at the junction (Figure S9H).

Two sets of different resonances were observed for the symmetry-related protons of Phen-DC3 upon binding. These exhibit no exchange crosspeaks in a ROESY experiment on a mixture with a 1:1 *QD3-sbl* – Phen-DC3 molar ratio, indicating only slow ring flips of the ligand when bound to the hybrid (not shown). Proton resonances of the ligand were fully assigned by standard strategies using COSY correlations to identify groups of scalar coupled protons assisted by NOESY experiments to link the different spin systems (Figure S7, S9). The two most downfield-shifted isochronous H4 amide protons at about 11.8 ppm serve as a convenient starting point by exhibiting NOE contacts to the quinoline moiety including *N*-methyl protons (Figure S9A).

Various contacts of phenanthroline protons H1, H2, and H3 but also of the H4 amide and quinoline protons closest to the phenanthroline moiety with residues G18, C19, G35, and G36 point to their location at the Q-D junction (Figure S7 and S9). On the other hand, quinoline protons distant from the phenanthroline ring system exhibit NOE contacts to the exposed interfacial G-tetrad residues G9 and G14. These connectivities suggest

the phenanthroline to be sandwiched between base pair and G-tetrad at the Q-D junction whereas both quinoline side arms project towards the open part of the G-tetrad.

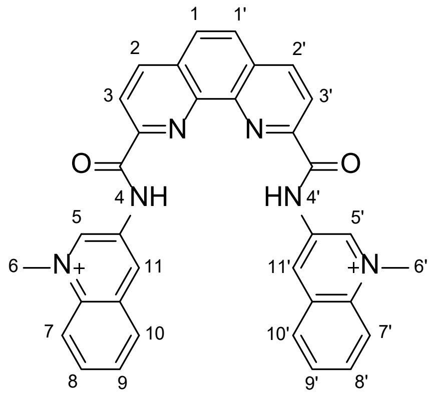

Chemical structure of Phen-DC3 with atom numbering as used in this study.

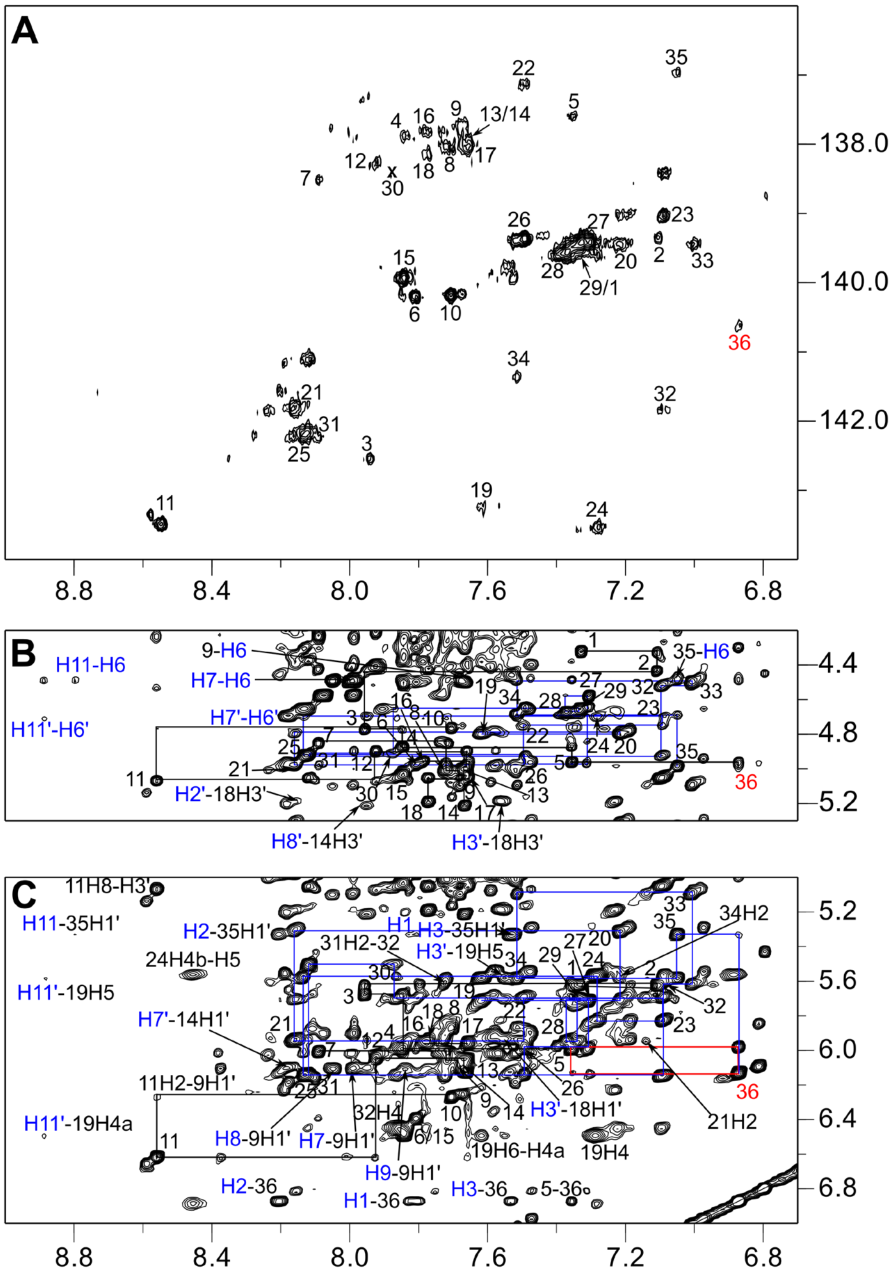

Figure S7. NMR spectra with assignments for a 1:1 complex of *QD3-sbl* (0.5 mM) with Phen-DC3 acquired at 30 °C in 10 mM potassium phosphate buffer, pH 7. (A) ^{1}H-^{13}C HSQC spectral region with H6/H8(ω_2)-C6/C8(ω_1) correlations; the crosspeak with downfield-shifted ^{13}C8 of *syn*-G36 is labeled in red; a G30 H8-C8 correlation only observed at lower threshold levels is marked by a cross. (B) H6/H8(ω_2)-H3'(ω_1) and (C) H6/H8(ω_2)-H1'(ω_1) spectral region of a NOESY spectrum (300 ms mixing time). NOE sequential connectivities for the quadruplex and the duplex domain are traced by black and blue lines, respectively. *Syn*-G36 with its strong intra-nucleotide H8-H1' crosspeak shows a weak NOE contact of its H1' proton to G5 H8 across the *anti-syn* step with antiparallel strand orientation (highlighted by the red rectangular pattern). Intra- and intermolecular ligand contacts are labeled with the ligand proton written in blue.

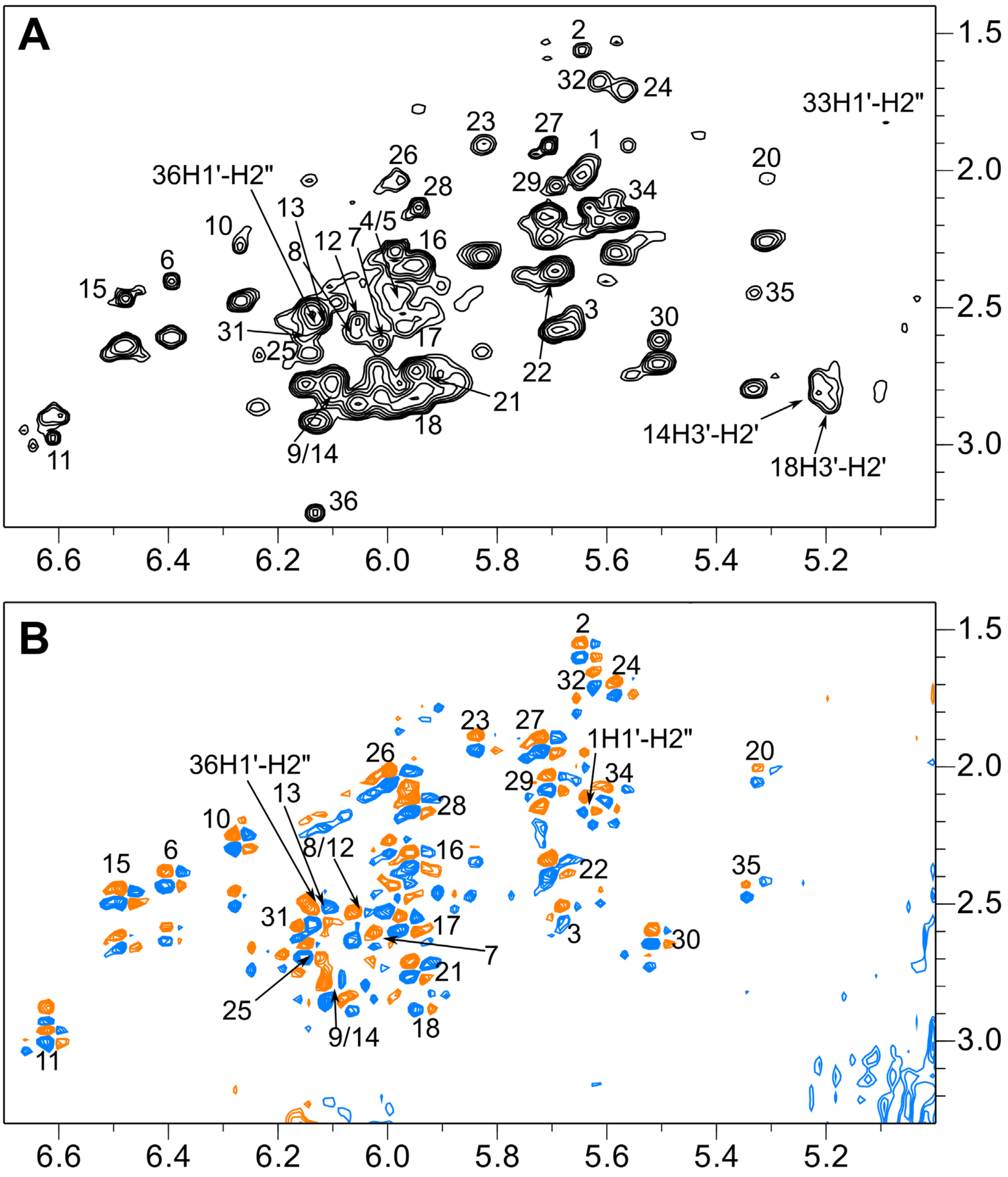

Figure S8. (A) NOESY spectrum (80 ms mixing time) and (B) DQF-COSY spectrum for a 1:1 complex of *QD3-sbl* with Phen-DC3, showing the H1'(ω_2)-H2'/H2"(ω_1) spectral region (30 °C, 10 mM potassium phosphate buffer, pH 7). Following a stereospecific H2'/H2" assignment based on NOE crosspeak intensities, sugar conformations can be assessed through inspection of DQF-COSY crosspeak patterns, demonstrating a *north*-type sugar pucker for residues T1 and G36.

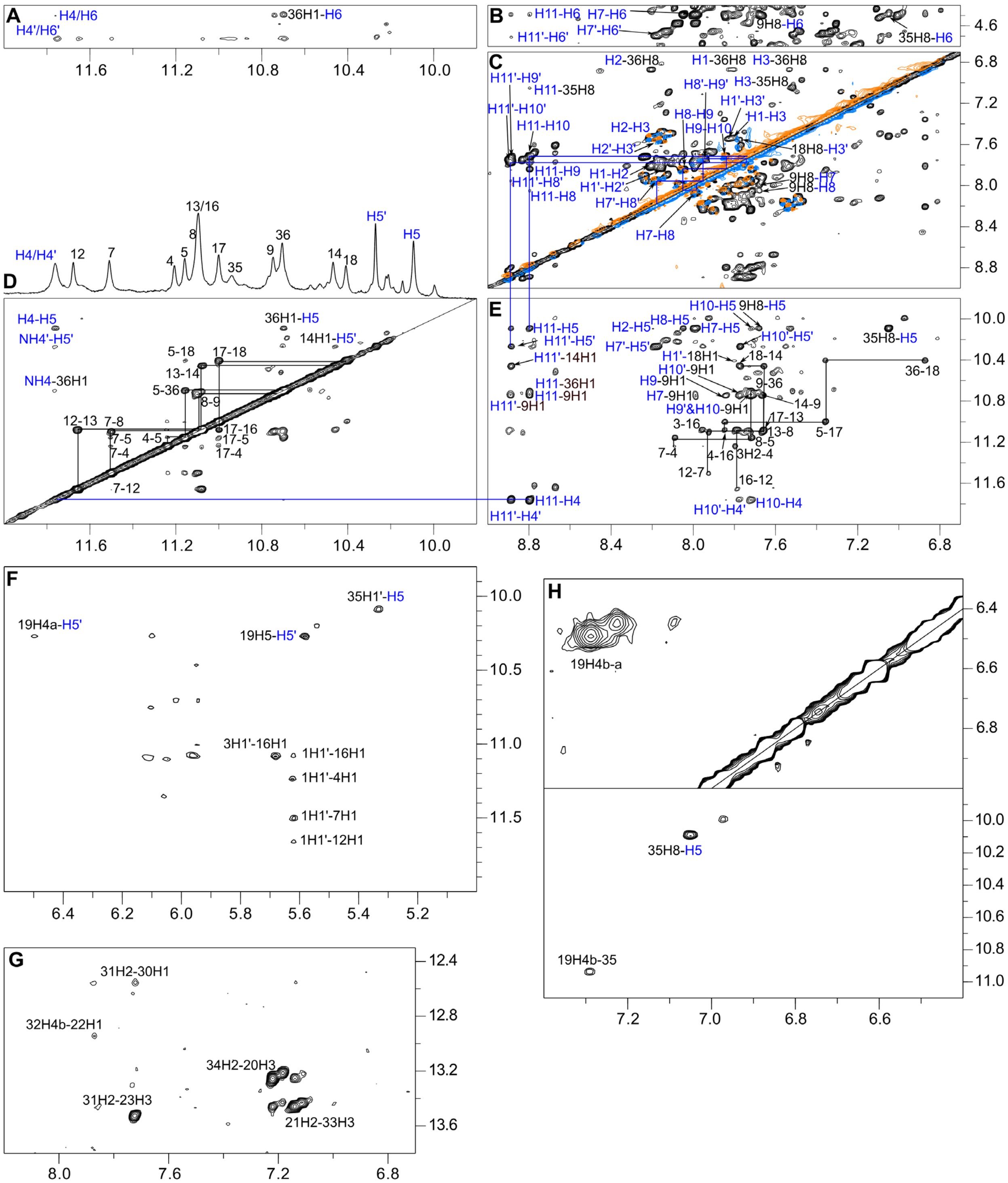

Figure S9. Resonance assignments of a 1:1 Phen-DC3 - *QD3-sbl* complex and intermolecular DNA-ligand contacts. (A,B) NOESY spectral regions showing inter- and intramolecular NOE contacts to the *N*-methyl H6/H6'(ω_1) protons of Phen-DC3. (C) Superposition of a NOESY (black) and DQF-COSY spectrum with assignments of ligand resonances through both scalar couplings and NOE connectivities. (D) NOESY spectral region with imino(ω_2)-imino(ω_1) crosspeaks and corresponding 1D spectrum with Hoogsteen G imino resonances (top). (E) H6/8(ω_2)-imino(ω_1) NOESY spectral region also comprising intra- and intermolecular crosspeaks of Phen-DC3 protons. (F) H1'(ω_2)-imino(ω_1) NOE crosspeaks with additional ligand H5/H5' contacts. (G) C amino-G imino and A H2-T H3 NOE contacts for the duplex domain with Watson-Crick hydrogen bonds. (H) Intra-base C19 amino(ω_2)-amino(ω_1) contact (top) and intra-base pair C19 amino(ω_2)-C35 imino(ω_1) contact (bottom). DNA and ligand protons are labeled with black and blue color, respectively. NOESY spectra were acquired at 30 °C in 10 mM potassium phosphate buffer, pH 7, with a 300 ms mixing time except for the spectral region in (H) that derives from a NOESY experiment with an 80 ms mixing time.

Table S5. ^{1}H and ^{13}C NMR chemical shifts δ of a *QD3-sbl* - Phen-DC3 1:1 complex[a]

δ / ppm	H8/H6	H1/H3	H1'	H2'/H2"	H3'	H5/H2/Me	C8/C6	C2
T1	7.33	n.d.	5.62	1.95/2.14	4.33	1.55	139.43	-
T2	7.11	n.d.	5.63	1.58/2.01	4.44	1.44	139.35	-
A3	7.96	-	5.68	2.54/2.58	4.77	7.79	142.54	n.d.
G4	7.85	11.24	5.99	2.49/2.87	4.87	-	137.87	-
G5	7.36	11.16	5.98	2.45/2.37	4.97	-	137.60	-
T6	7.81	n.d.	6.39	2.40/2.61	4.98	1.94	140.21	-
G7	8.09	11.50	6.01	2.62/2.83	4.86	-	138.52	-
G8	7.72	11.10	6.05	2.59/2.84	5.01	-	138.04	-
G9	7.68	10.74	6.10	2.82/2.79	5.10	-	137.77	-
T10	7.70	n.d.	6.27	2.27/2.48	4.77	1.98	140.17	-
A11	8.56	-	6.62	2.99/2.90	5.07	8.37	143.50	154.37
G12	7.92	11.66	6.05	2.55/2.85	4.90	-	138.27	-
G13	7.66	11.08	6.12	2.55/2.91	5.00	-	138.01	-
G14	7.67	10.46	6.10	2.81/2.76	5.21	-	138.01	-
T15	7.84	n.d.	6.48	2.47/2.64	5.03	2.00	139.94	-
G16	7.79	11.08	5.95	2.32/2.73	4.96	-	137.80	-
G17	7.66	11.00	5.96	2.57/2.85	5.05	-	138.02	-
G18	7.77	10.41	5.94	2.85/2.80	5.19	-	138.13	-
C19	7.62	-	5.71	2.20/2.25	4.80	5.58	143.24	-
T20	7.21	13.25	5.31	2.03/2.25	4.80	1.52	139.46	-
A21	8.16	-	5.94	2.74/2.82	4.98	7.14	141.81	153.84
G22	7.50	12.94	5.69	2.36/2.58	4.75	-	137.11	-
T23	7.09	13.52	5.83	1.91/2.31	4.69	1.19	139.04	-
C24	7.28	-	5.58	1.71/2.18	4.70	5.57	143.55	-
A25	8.13	-	6.14	2.66/2.51	4.93	7.86	142.21	154.89
T26	7.49	n.d.	5.99	2.03/2.29	4.97	1.74	139.36	-
T27	7.31	n.d.	5.71	1.91/2.17	4.58	1.57	139.42	-
T28	7.37	n.d.	5.95	2.14/2.35	4.67	1.59	139.57	-
T29	7.33	n.d.	5.70	2.05/2.37	4.66	1.77	139.48	-
G30	7.87	12.55	5.50	2.61/2.70	4.91	-	138.37	-
A31	8.12	-	6.14	2.59/2.77	4.92	7.72	142.16	155.03
C32	7.09	-	5.62	1.67/2.18	4.52	5.05	141.83	-
T33	7.01	13.46	5.09	1.66/1.83	4.49	1.28	139.45	-
A34	7.51	-	5.58	2.10/2.30	4.69	7.22	141.37	153.93
G35	7.05	10.94	5.33	2.44/2.80	4.99	-	136.98	-
G36	6.87	10.70	6.13	3.25/2.55	5.00	-	140.61	-

[a]At 30 ºC in 10 mM potassium phosphate buffer, pH 7.0.

Table S6. ^{1}H NMR chemical shifts δ (ppm) of Phen-DC3 bound to *QD3-sbl*[a]

H1	H2	H3	H4	H5	6-CH3	H7	H8	H9	H10	H11
7.81	8.20	7.53	11.76	10.09	4.49	7.99	8.05	7.84	7.71	8.80
H1’	H2’	H3’	H4’	H5’	6-CH3’	H7’	H8’	H9’	H10’	H11’
7.82	8.16	7.55	11.76	10.27	4.69	8.18	7.95	7.73	7.77	8.89

[a]At 30 ºC in 10 mM potassium phosphate buffer, pH 7.0.

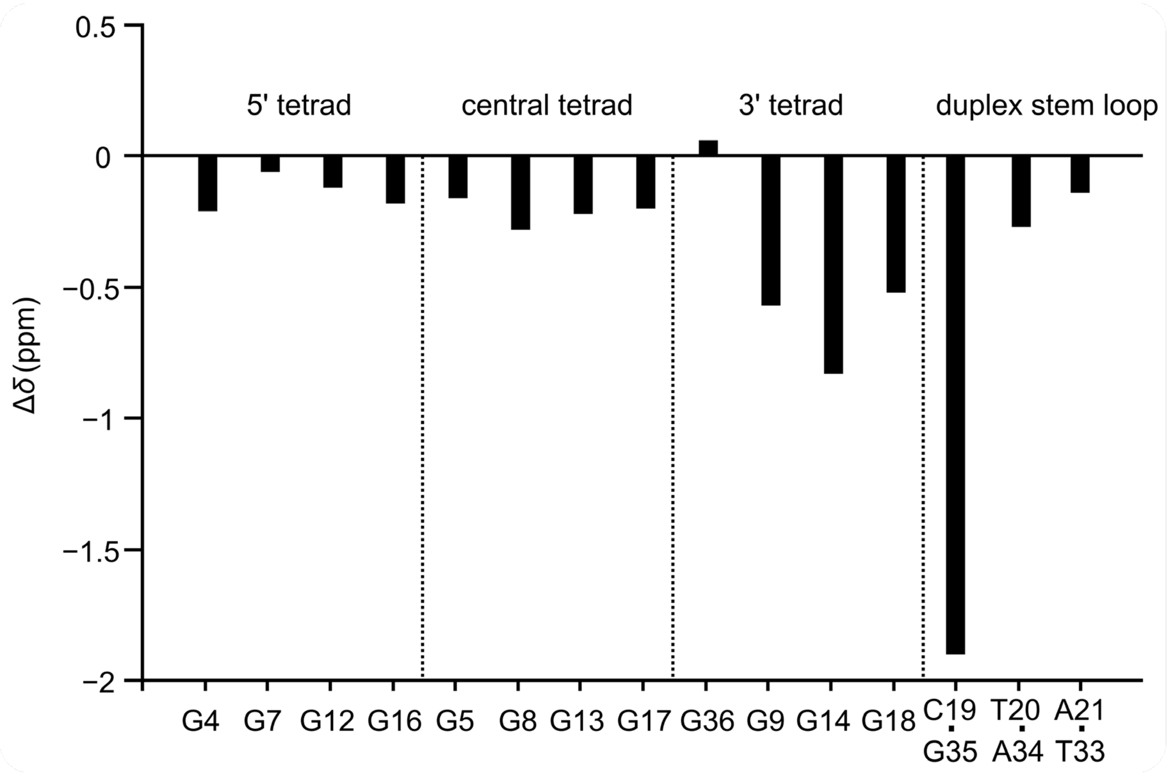

Figure S10. Chemical shift perturbations $\Delta\delta$ of imino protons in *QD3-sbl* upon binding the Phen-DC3 ligand (120 mM K^+).

Resonance assignments of free *Q3-sbl2* and of its complex with Phen-DC3

Non-interrupted base-sugar NOEs can be traced from T1 to G5 and *syn*-G22, demonstrating a first truncated G-column with a broken G-tract and the open position filled by the 3’-terminal G22 (S11C). In analogy to *QD3-sbl*, second and third G-columns can be distinguished by following contacts of A11 in the second propeller loop. While a continuous NOE walk can be traced from G16 to T19, a long-range contact between G20 and G18 identifies the fourth G-column and the TGT lateral snapback loop. The presence of a single *syn*-guanosine at position 22 is additionally corroborated by its typical ^{13}C8 chemical shift in ^{1}H-^{13}C HSQC spectra (Figure S11B). Based on a parallel quadruplex, imino protons were unambiguously assigned without specific isotope labeling by intra- and inter-tetrad H8-imino connectivities and further confirmed by their sequential imino-imino contacts (Figure S11D,E). Also, a NOE crosspeak between the G22 imino and the T21 H6 proton demonstrates positioning of the snapback loop above the 3’-tetrad, effectively protecting an observable G22 amino proton from solvent exchange.

Assignments for the quadruplex in the 1:1 complex with Phen-DC3 closely follows the assignments of the free *Q3-sbl2*, demonstrating a conserved parallel topology, a lateral snapback loop, homopolar tetrad stacking, and a G22 amino proton protected from solvent exchange (Figure S12). Various contacts from Phen-DC3 quinoline protons to the quadruplex can be observed, including contacts to all G imino protons in the 5’-tetrad but also to T1 H1’ in the 5’-overhang. These unambiguously show that the Phen-DC3 ligand stacks onto the 5’-face of the quadruplex (Figure S12C-F). However, some unidentified crosspeaks to protons of the 3’-tetrad also suggest small amounts of a minor complex with a putative Phen-DC3 binding at the 3’-tetrad (Figure S12D). Interestingly, in contrast to Phen-DC3 intercalated at the Q-D junction of *QD3-sbl*, symmetry-related ligand protons are subject to chemical exchange through a flip of the ligand as shown by exchange crosspeak in a ROESY experiment (Figure S13).

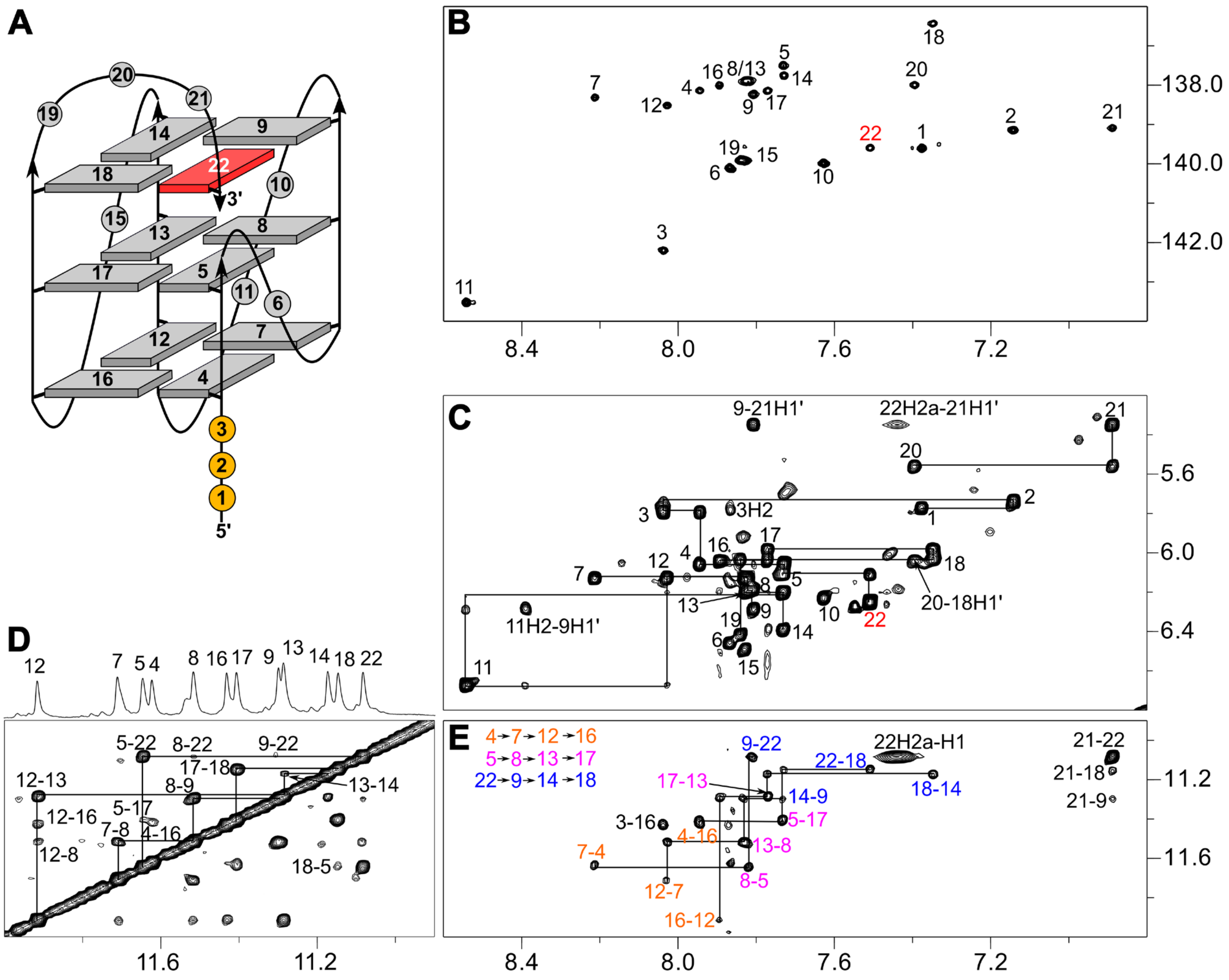

Figure S11. (A) Topology of *Q3-sbl2.* (B) ^{1}H-^{13}C HSQC spectrum of *Q3-sbl2* with H6/H8(ω2)-C6/C8(ω1) correlations; a single *syn*-G22 is labeled in red. (C) H6/H8(ω2)-H1'(ω1) NOESY spectral region of *Q3-sbl2* with the strong intra-nucleotide crosspeak of *syn-G22* labeled in red. (D) Imino(ω2)-imino(ω1) NOESY spectral region and corresponding 1D spectrum with assignments (top). (E) H6/H8(ω2)-imino(ω1) NOESY spectral region with intra-tetrad contacts labeled in different color for each tetrad; the latter determine tetrad polarities and are indicative of exclusive homopolar stacking for *Q3-sbl2*; lines trace sequential connectivities between tetrads. NOESY (300 ms mixing time) and HSQC spectra were acquired at 30 °C in 10 mM potassium phosphate buffer, pH 7.0, with a strand concentration of 0.75 mM.

Table S7. ^{1}H and ^{13}C NMR chemical shifts δ for free *Q3-sbl2*[a]

δ / ppm	H8/H6	H1/H3	H1'	H2'/H2"	H3'	H5/H2/Me	C8/C6	C2
T1	7.38	n.d.	5.77	2.04/2.24	4.45	1.64	139.61	-
T2	7.14	n.d.	5.74	1.66/2.12	4.54	1.54	139.15	-
A3	8.04	-	5.79	2.57/2.66	4.85	7.87	142.22	153.93
G4	7.94	11.63	6.06	2.56/2.89	5.02	-	138.14	-
G5	7.73	11.64	6.11	2.76/2.45	5.07	-	137.50	-
T6	7.87	n.d.	6.46	2.43/2.67	5.07	1.97	140.10	-
G7	8.21	11.71	6.13	2.87/2.87	4.89	-	138.34	-
G8	7.82	11.52	6.19	2.62/2.84	5.00	-	137.91	-
G9	7.81	11.30	6.29	2.66/2.50	4.98	-	138.23	-
T10	7.63	n.d.	6.23	2.20/2.44	4.68	1.90	139.99	-
A11	8.54	-	6.67	3.07/2.92	5.15	8.39	143.53	155.12
G12	8.03	11.91	6.13	2.57/2.98	4.99	-	138.52	-
G13	7.83	11.29	6.20	2.66/3.00	5.00	-	137.91	-
G14	7.73	11.17	6.39	2.68/2.53	5.12	-	137.75	-
T15	7.83	n.d.	6.49	2.45/2.66	5.06	1.96	139.92	-
G16	7.89	11.43	6.04	2.39/2.80	5.04	-	138.00	-
G17	7.77	11.41	5.98	2.56/2.75	5.04	-	138.15	-
G18	7.35	11.15	6.04	2.68/2.67	5.10	-	136.44	-
T19	7.84	n.d.	6.42	2.37/2.63	4.75	1.96	139.91	-
G20	7.40	n.d.	5.56	1.89/2.16	4.59	-	138.00	-
T21	6.89	n.d.	5.35	1.76/2.31	4.48	1.63	139.10	-
G22	7.51	11.08	6.25	3.12/2.59	4.95	-	139.60	-

[a]At 30 ºC in 10 mM potassium phosphate buffer, pH 7.0.

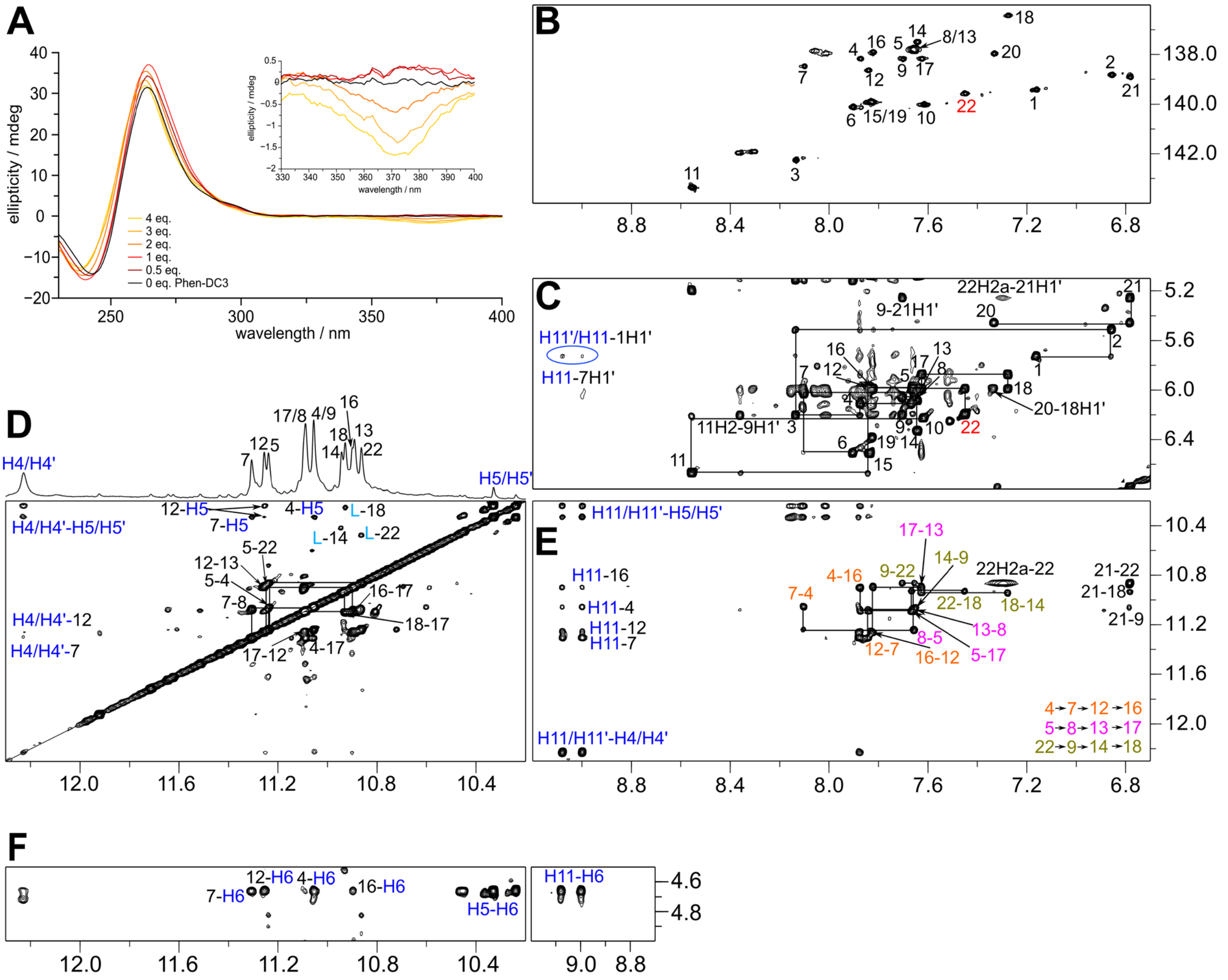

Figure S12. (A) CD spectra of *Q3-sbl2* upon its titration with Phen-DC3; the inset shows the y-expanded ligand absorption region. (B) ^{1}H-^{13}C HSQC spectrum of the 1:1 *Q3-sbl2* - Phen-DC3 complex with H6/H8(ω_2)-C6/C8(ω_1) correlations; *syn*-G22 is labeled in red. (C-F) NOESY spectrum (300 ms mixing time) of a 1:1 *Q3-sbl2* - Phen-DC3 complex with ligand resonances labeled in blue. (C) H6/H8(ω_2)-H1'(ω_1) spectral region with the intra-nucleotide crosspeak of *syn-G22* labeled in red; two representative crosspeaks between Phen-DC3 protons and 5'-overhang residues are circled. (D) Imino(ω_2)-imino(ω_1) spectral region; unassigned ligand resonances L with NOE contacts to the 3'-tetrad, suggesting the presence of a minor complex, are labeled in light blue; a corresponding 1D spectrum with assignments is shown on top. (E) H6/H8(ω_2)-imino(ω_1) spectral region with intra-tetrad contacts labeled in different color for each tetrad; lines trace sequential connectivities between tetrads. (F) Crosspeaks of Phen-DC3 *N*-methyl protons H6(ω_1) with G4 imino and Phen-DC3 protons. Spectra were acquired at 30 °C in 10 mM potassium phosphate buffer, pH 7.0, and a strand concentration of 0.75 mM.

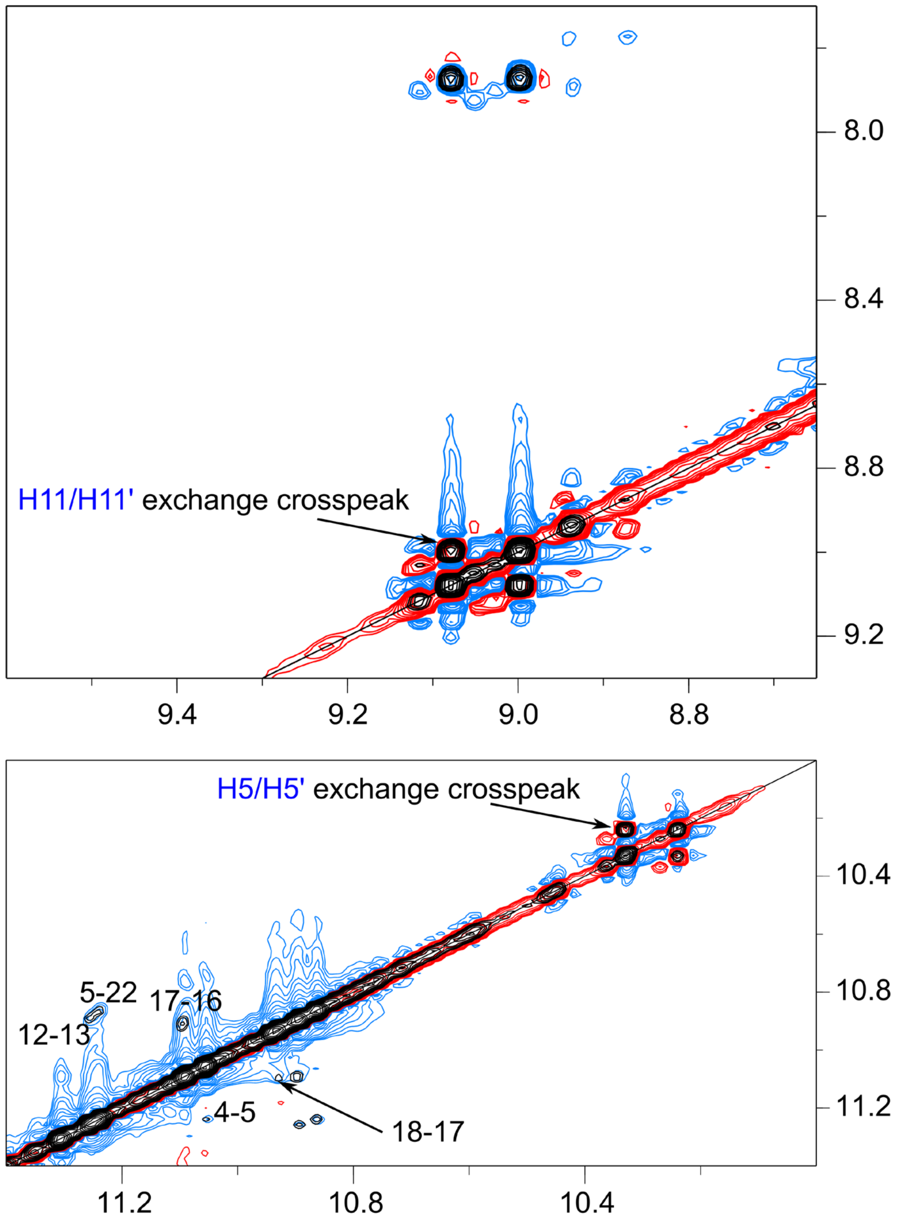

Figure S13. Superposition of NOESY (300 ms mixing time) and ROESY spectral regions for the 1:1 complex of *Q3-sbl2* (0.75 mM) with Phen-DC3. ROESY exchange crosspeaks of the ligand with their positive sign are indicated. Spectra were acquired at 30 °C in 10 mM potassium phosphate buffer, pH 7.0. Signals in the NOESY spectrum are colored black whereas positive and negative signals in the ROESY spectrum are given in red and blue color, respectively.

Table S8. ^1H and ^{13}C NMR chemical shifts δ for a *Q3-sbl2* - Phen-DC3 1:1 complex[a]

δ / ppm	H8/H6	H1/H3	H1'	H2'/H2"	H3'	H5/H2/Me	C8/C6	C2
T1	7.16	n.d.	5.73	1.84/2.11	4.41	1.52	139.43	-
T2	6.86	n.d.	5.52	1.47/1.92	4.50	1.36	138.83	-
A3	8.14	-	6.20	2.88/2.96	5.11	7.75	142.25	155.03
G4	7.87	11.06	6.11	2.51/2.89	5.02	-	138.19	-
G5	7.67	11.24	5.99	2.76/2.43	5.07	-	137.81	-
T6	7.90	n.d.	6.51	2.47/2.70	5.12	2.00	140.13	-
G7	11.31	8.10	6.03	2.60/2.87	4.91	-	138.49	-
G8	11.09	7.66	6.06	2.51/2.78	4.95	-	137.79	-
G9	11.06	7.70	6.21	2.60/2.45	4.95	-	138.19	-
T10	7.62	n.d.	6.23	2.18/2.43	4.69	1.89	140.01	-
A11	8.56	-	6.67	3.07/2.95	5.20	8.36	143.39	155.32
G12	7.84	11.26	5.99	2.48/2.91	5.03	-	138.67	-
G13	7.66	10.89	6.08	2.57/2.93	4.98	-	137.81	-
G14	7.64	10.94	6.33	2.63/2.50	5.08	-	137.50	-
T15	7.84	n.d.	6.51	2.46/2.68	5.07	1.96	139.92	-
G16	7.82	10.90	5.99	2.25/2.77	5.08	-	137.93	-
G17	7.63	11.09	5.87	2.47/2.66	5.00	-	138.18	-
G18	7.28	10.93	5.99	2.62/2.62	5.06	-	136.44	-
T19	7.83	n.d.	6.39	2.34/2.59	4.34	1.95	139.91	-
G20	7.33	n.d.	5.46	1.82/2.09	4.52	-	137.98	-
T21	6.78	n.d.	5.26	1.69/2.24	4.43	1.51	138.92	-
G22	7.45	10.86	6.20	3.04/2.51	4.83	-	139.57	-

[a]At 30 ºC in 10 mM potassium phosphate buffer, pH 7.0.

Resonance assignments of free *QD2-l* and of its complex with PIQ-4m

The imino proton spectral region of the *QD2-l* hybrid shows six and eight imino resonances with chemical shifts typical of Watson-Crick base pairs and of more upfield shifted G iminos involved in Hoogsteen hydrogen bonds, respectively. Identifying four *syn-anti* steps along the G-columns, a two-layered antiparallel G-quadruplex can be established. The first and third G-columns were assigned based on continuous sugar-base NOE contacts to the following TT lateral loops. The second G-column is identified due to non-interrupted NOE connectivities from *syn*-G5 along the duplex stem loop up to G21 (Figure S14C,D). Various contacts at the Q-D interface including G21 H8 to G22 H1 and C7 H4 to G6 H1 positions the duplex stem loop coaxially with the G-core. A typical heteropolar stacking pattern can be observed through strong non-sequential imino-imino contacts such as between G26 H1 and G22 H1. G-tetrad polarity following hydrogen bond donor to acceptor runs along G1→G6→G22→G27 and G2→G26→G23→G5. Intra-tetrad NOE contacts between G imino and G H8 protons of either *syn-* or *anti*-Gs determine the quadruplex groove width. Thus, the two TT lateral loops bridge a narrow groove while the duplex stem loop bridge the wide groove of the quadruplex.

For the 1:1 complex of *QD2-l* with the PIQ-4m ligand, similar sugar-base NOE connectivities as found for the free hybrid identify the first and third G-column followed by the two TT lateral loops and the duplex hairpin loop with uninterrupted NOE connectivities from C7 to G21. However, interruption of sequential contacts at the junction from G6 to C7 indicates ligand intercalation. *Syn*-guanines were assigned by their downfield-shifted ^{13}C8 resonance while three adenine H2 resonances were identified by their H2-C2 correlations in a ^{1}H-^{13}C HSQC spectrum (Figure S16C). Stereospecific assignments of H2'/H2" protons were based on a NOESY experiment with short mixing times (80 ms) and the following determination of sugar conformations made use of the pattern and intensity of H1'-H2' and H1'-H2" crosspeaks in a DQF-COSY spectrum (Figure S17). Except for T3 and T24, located in lateral loops bridging the narrow groove, all assigned sugar puckers are in the *south* domain of the pseudorotational cycle. Imino protons were assigned by following exchange crosspeaks between the free and complexed *QD2-l* hybrid (Figure S15) and heteropolar tetrad stacking was confirmed by characteristic intra- and inter-tetrad H8-imino NOE contacts. Taken together, the two-layered antiparallel topology of free *QD2-l* with exclusive *syn-anti* steps along the G-columns, two TT lateral loops bridging a narrow groove, and one duplex stem loop bridging a wide groove was retained after ligand addition. However, sequential contacts bridging the quadruplex-duplex interface were lost.

Ligand proton resonances were assigned by a combination of COSY and NOESY experiments. Spin systems with corresponding COSY correlations derive from protons in the phenyl and the fused indole and quinoline ring systems (Figure S18F). Discrimination of quinoline and indole resonances was enabled by a strong NOE crosspeak from a phenyl proton to quinoline H1 and H2 protons. Additional NOE crosspeaks of methyl substituents of the quinoline and indole moiety were observed to ring protons in their proximity, with the quinoline *N*-methyl proton resonating close to the water signal (Figure S18D,E). A strong NOE contact connects the indole NH with a resonance at about 8 ppm (Figure S18G). TOCSY and ROESY experiments identified the latter as being two isochronous ortho-positioned phenyl protons, explaining the observation of only a single scalar coupled proton pair of the phenyl ring in a DQF-COSY spectrum. The NH16 amide is assigned by following NOE contacts from adjacent phenyl protons with connectivities continuing to the aliphatic

side chain (Figures S18F, S19A). Methylene protons H17 adjacent to the amide were found to be non-equivalent, indicating a restricted C-C bond rotation upon binding. Other resonances of the aliphatic side chain were identified through their mutual scalar couplings observed in COSY and TOCSY experiments (Figure S19B). It should be noted that NOE crosspeaks for aliphatic side chain protons are rather weak and broadened due to a high flexibility towards the terminus with changing signs for NOE crosspeaks of terminal ethyl protons (not shown).

Various intermolecular ligand-DNA contacts were observed in representative NOESY spectral regions, yielding a total of 41 NOESY-derived distance restraints (Figures S16 and S18). In fact, indole H1/H2/H3 protons primarily show NOE contacts to G6 and C7 while quinoline H6/H7/H8 protons show contacts to G21 and G22 residues. Quinoline methyl substituents H4a and H5a are connected to G6 and G21 iminos (Figure S18A,B). Interestingly, indole methyl protons H9a located on the opposite side of the indoloquinoline together with its extended side chain feature a contact to the exposed G1 imino not covered by the duplex stem loop (Figure S18A). Additionally, phenyl protons, in particular H14 and H15, show NOE contacts to all imino protons within the G-tetrad at the Q-D interface. Such a NOE pattern suggests the indoloquinoline to be sandwiched at the Q-D junction with its side chain projected towards the exposed part of the interfacial G-tetrad.

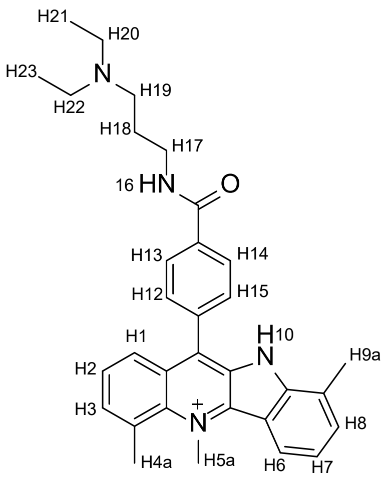

Chemical structure of PIQ-4m with atom numbering as used in this study.

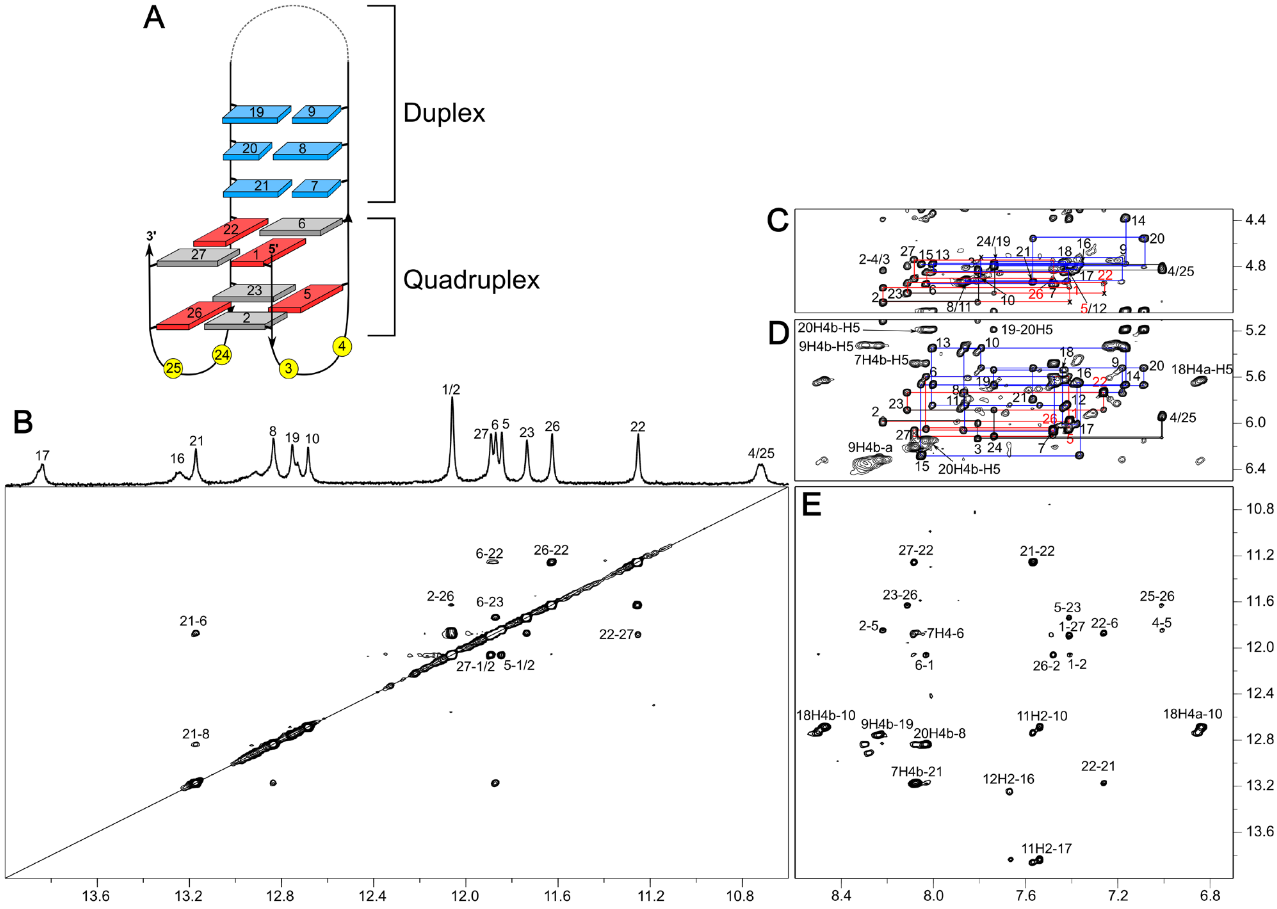

Figure S14. (A) Topology and (B-E) NOESY spectral regions of the *QD2-l* hybrid. (B) Imino(ω2)-imino(ω1) crosspeaks and corresponding 1D spectrum with assignments on top. (C) H6/H8(ω2)-H3'(ω1) and (D) H6/H8(ω2)-H1'(ω1) spectral region; continuous NOE connectivities in the quadruplex and duplex domain are traced by black and blue lines, respectively. Characteristic rectangular patterns for *syn-anti* steps and labels of *syn*-residues G1, G5, G22, and G26 with their strong intra-nucleotide H8-H1' crosspeak are shown in red. Two NOE contacts in (C) only observed at lower threshold levels are marked by a cross. (E) H6/H8(ω2)-imino(ω1) crosspeaks of the G-core and adenine H2/cytosine amino(ω2)-imino(ω1) crosspeaks of the duplex domain. NOESY spectra (300 ms mixing time) were acquired with a *QD2-l* concentration of 0.64 mM at 30 °C in 20 mM potassium phosphate buffer, 100 mM KCl, pH 7.0.

Note: A three-dimensional NMR structure of the same sequence under different temperature and buffer conditions has been published previously (PDB 2M8Z).[2] It matches the Q-D hybrid topology as derived here from analysis of the NMR spectral data collected under the present conditions. Assignments of T4/T25 imino protons lacking NOE crosspeaks in the present study is based on a comparison with the previously published structural analysis.

2. Lim, K. W.; Phan, A. T. Structural Basis of DNA Quadruplex-Duplex Junction Formation. Angew. Chem. Int. Ed. 2013, 52, 8566–8569.

Table S9. ^{1}H NMR chemical shifts δ for free *QD2-I*[a]

δ / ppm	H8/H6	H1/H3	H1'	H2'/H2''[b]	H3'	H5/H2/Me
G1	7.41	12.06	5.98	2.90/2.90	4.98	-
G2	8.22	12.06	6.00	2.35/3.00	5.11	-
T3	7.81	n.d.	6.13	2.15/2.51	4.83	1.94
T4	7.01	n.d.	5.94	1.98/2.58	4.83	0.89
G5	7.41	11.85	6.05	2.98/3.39	4.85	-
G6	8.03	11.87	5.60	2.40/2.64	4.95	-
C7	7.48	-	6.06	1.88/2.40	4.95	5.48
G8	7.87	12.84	5.74	2.58/2.64	4.93	-
C9	7.18	-	5.52	1.76/2.22	4.72	5.33
G10	7.79	12.69	5.34	2.56/2.65	4.91	-
A11	7.86	-	5.84	2.25/2.57	4.93	7.54
A12	7.42	-	5.85	2.02/2.38	4.84	7.67
G13	8.00	n.d.	5.34	2.31/2.60	4.78	-
C14	7.17	-	5.66	1.56/2.02	4.38	5.19
A15	8.05	-	6.28	2.91/2.6	4.78	n.d.
T16	7.36	13.25	5.65	2.08/2.47	4.79	1.80
T17	7.38	13.84	6.01	2.15/2.44	4.83	1.59
C18	7.43	-	5.54	2.00/2.29	4.76	5.63
G19	7.74	12.75	5.67	2.43/2.48	4.80	-
C20	7.09	-	5.52	1.66/2.12	4.56	5.19
G21	7.57	13.17	5.79	2.55/2.94	4.93	-
G22	7.26	11.26	5.73	2.60/3.03	4.94	-
G23	8.11	11.74	5.89	2.26/2.87	5.03	-
T24	7.74	n.d.	6.11	2.08/2.46	4.80	1.88
T25	7.01	n.d.	5.94	1.97/2.54	4.80	0.83
G26	7.48	11.63	6.06	2.99/3.51	4.91	-
G27	8.08	11.89	6.11	2.38/2.62	4.74	-

[a]At 30 ºC in 20 mM potassium phosphate buffer, 100 mM KCl, pH 7.0. [b]No stereospecific assignment.

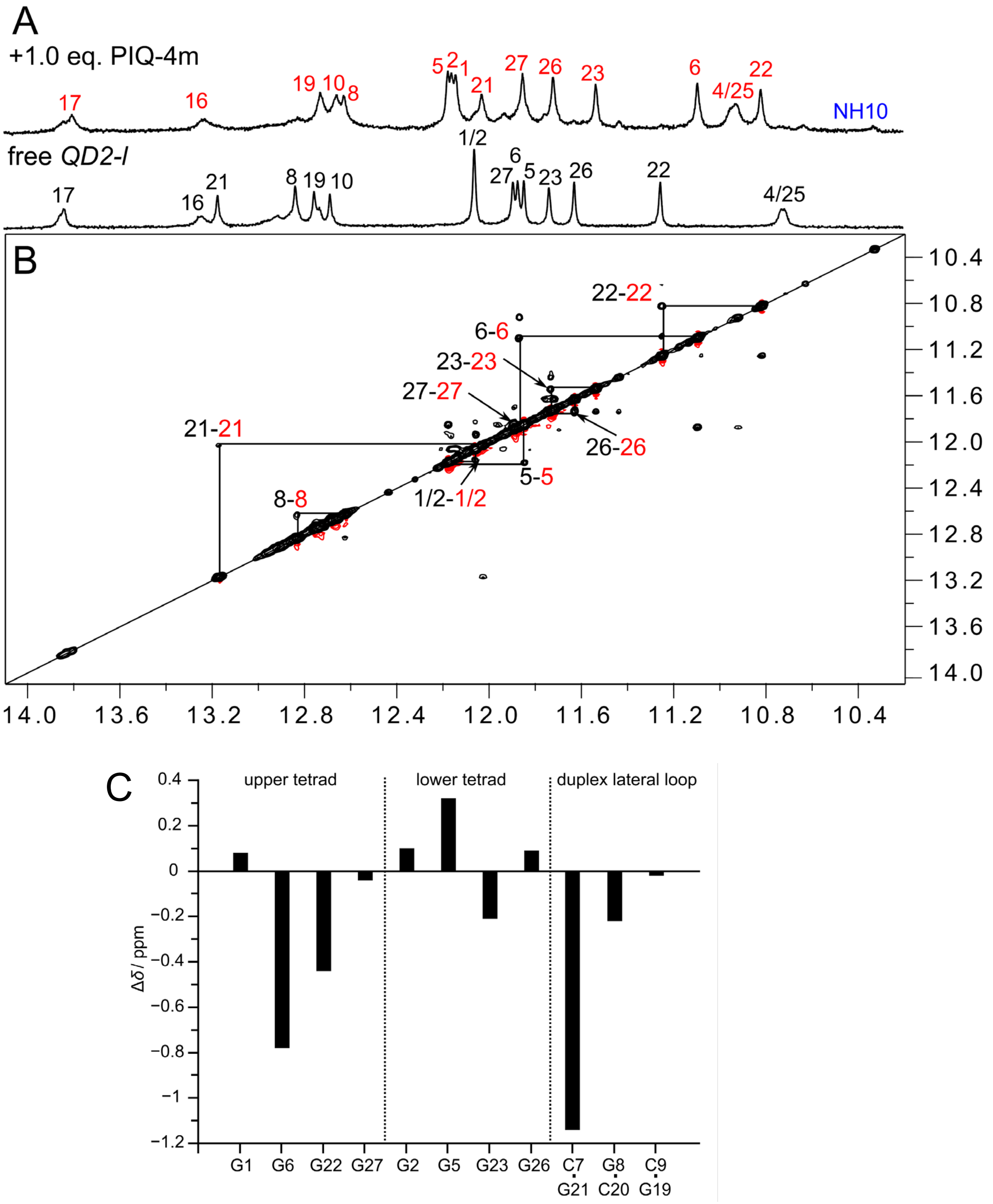

Figure S15. (A) 1D imino proton spectral region with resonance assignments of *QD2-l* before (bottom) and after addition of 1 equivalent of PIQ-4m (top). (B) Imino(ω_2)-imino(ω_1) spectral region of a ROESY spectrum for *QD2-l* with 0.5 equivalent of PIQ-4m; exchange crosspeaks of positive sign are colored black and signals of opposite sign colored red; imino proton resonances in free and ligand-bound *QD2-l* are labeled in black and red, respectively; NH10 represents a ligand resonance. Spectra were acquired at 30 °C in 20 mM potassium phosphate buffer, 100 mM KCl, pH 7.0, with a *QD2-l* concentration of 0.64 mM. (C) Imino proton chemical shift perturbations upon PIQ-4m binding to *QD2-l*. For a compilation of chemical shifts for the free Q-D hybrid and its complex with ligand see Tables S8-S10.

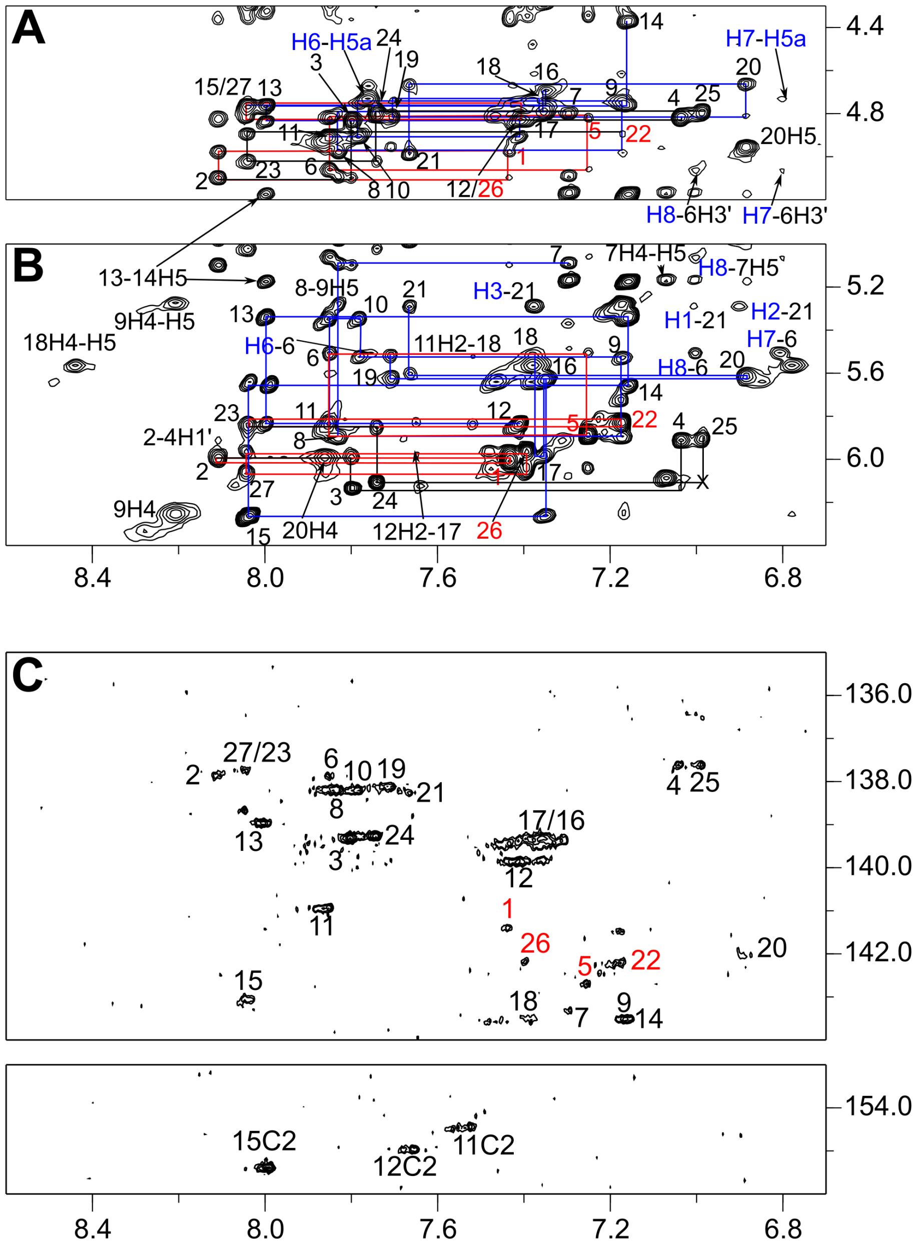

Figure S16. Resonance assignments for the 1:1 complex between *QD2-l* (0.64 mM) and PIQ-4m. (A) NOESY spectral region with H6/H8(ω_2)-H3'(ω_1) and (B) H6/H8(ω_2)-H1'(ω_1) crosspeaks. Sequential NOE connectivities for the quadruplex and duplex domain are traced by black and blue lines, respectively. Also shown are some ligand-DNA intermolecular contacts with ligand resonances labeled in blue. A T25 H6-T24 H1' crosspeak only observed at lower threshold levels is marked by a cross. (C) ^{1}H-^{13}C HSQC spectrum showing H6/H8(ω_2)-C6/C8(ω_1) (top) and adenine H2(ω_2)-C2(ω_1) correlations (bottom). *Syn*-residues G1, G5, G22, and G26 exhibiting strong intra-nucleotide H8-H1' NOE crosspeaks and characteristic rectangular patterns for *syn-anti* steps (A,B) as well as more downfield shifted guanine ^{13}C8 resonances (C) are labeled in red. Experiments were performed at 30 °C in 20 mM potassium phosphate buffer, 100 mM KCl, pH 7.0. NOESY spectra were acquired with a 300 ms mixing time.

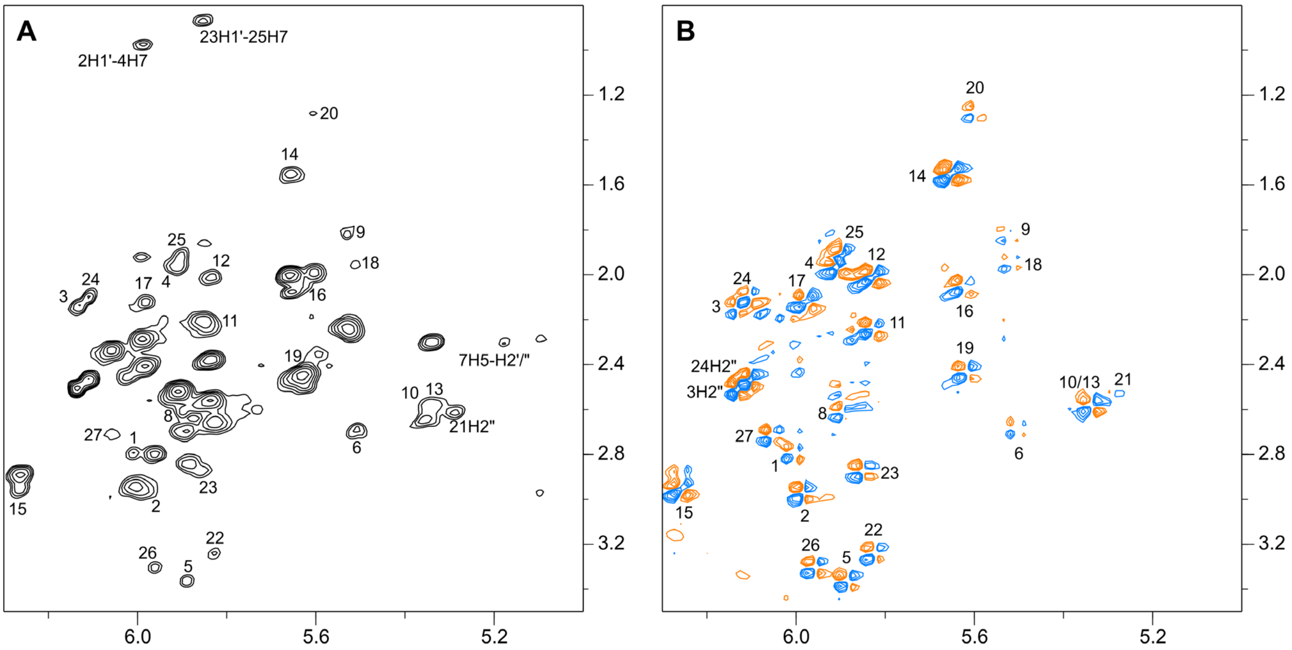

Figure S17. (A) NOESY spectrum (80 ms mixing time) and (B) DQF-COSY spectrum for a 1:1 complex of *QD2-l* (0.64 mM) with PIQ-4m, showing the H1'(ω_2)-H2'/H2"(ω_1) spectral region (30 °C, 20 mM potassium phosphate buffer, 100 mM KCl, pH 7). Following stereospecific H2'/H2" assignments based on NOE crosspeak intensities, sugar conformations were evaluated through inspection of DQF-COSY crosspeak patterns, demonstrating a *north*-type sugar conformation for T3 and T24 with all other residues adopting a *south* sugar pucker.

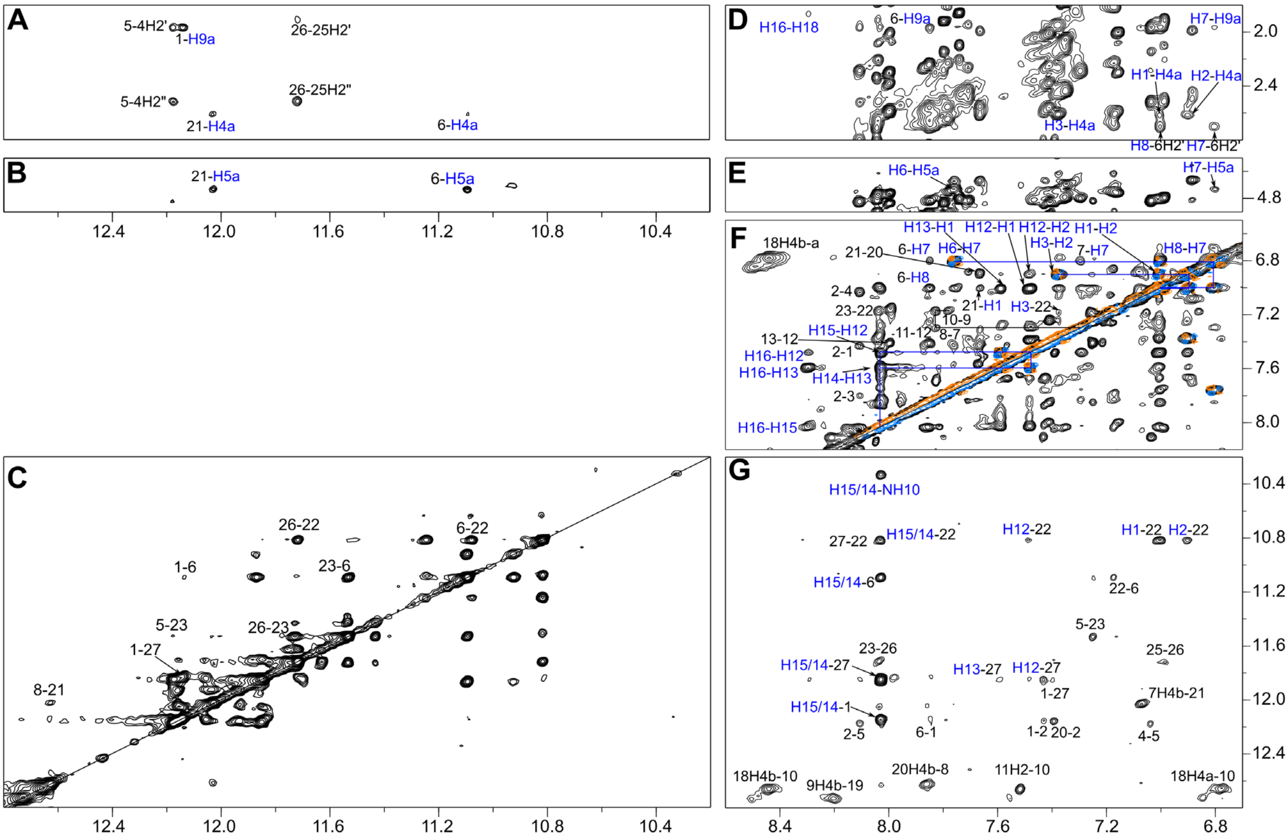

Figure S18. NOESY spectral regions for the 1:1 complex between *QD2-I* (0.64 mM) and PIQ-4m with assignments of ligand protons and intermolecular DNA-ligand contacts. (A,B) NOE contacts from imino(ω_2) to H2'/H2" and to ligand methyl protons, also including more deshielded PIQ-4m *N*-methyl (H5a) protons. (C) Imino(ω_2)-imino(ω_1) spectral region. (D,E) NOE contacts between aromatic protons(ω_2) and H2'/H2"/methyl protons(ω_1). (F) 2D NOE spectral region with intra- and intermolecular crosspeaks between aromatic protons. For the unambiguous assignment of the PIQ-4m aromatic ring protons, a corresponding DQF-COSY spectrum (orange-blue for positive-negative signals) is superimposed on the NOESY spectral region (black). (G) NOE contacts between aromatic protons(ω_2) and imino protons(ω_1). DNA and ligand protons are labeled in black and blue, respectively. NOESY spectra (300 ms mixing time) were acquired at 30 °C in 20 mM potassium phosphate buffer, 100 mM KCl, pH 7.0.

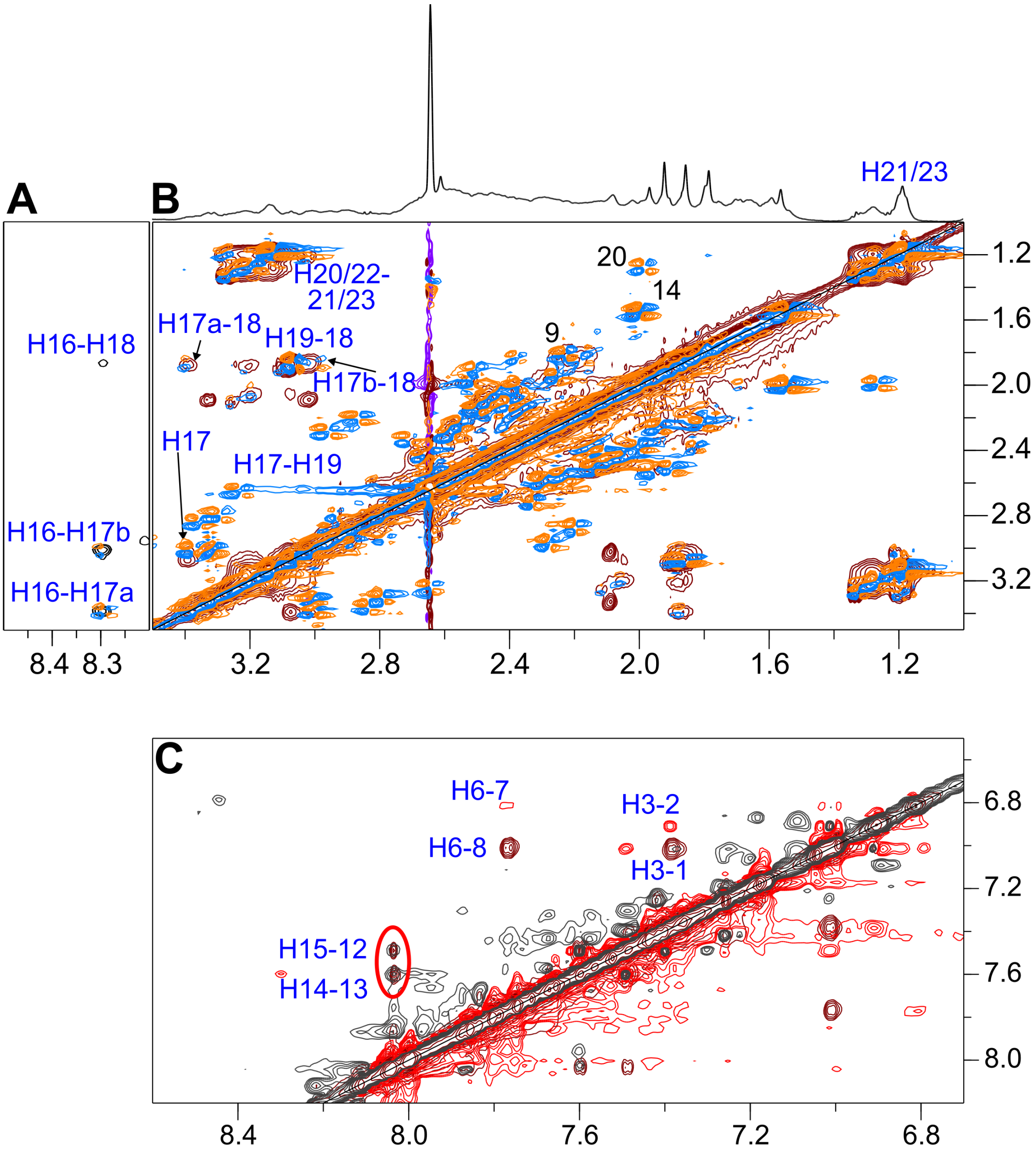

Figure S19. Proton assignments of the PIQ-4m side chain in a 1:1 complex with *QD2-I*. (A) Superposition of a NOESY (300 ms mixing time, black) and DQF-COSY spectrum (orange-blue for positive-negative signals) with correlations between amide NH16 and adjacent methylene protons. (B) Superposition of a DQF-COSY (orange-blue for positive-negative signals) and a TOCSY spectrum (brown), showing DNA intra-nucleotide H2'-H2" crosspeaks and correlations between resonances of the ligand aliphatic side chain including terminal H21/H23 with their triplet fine structure (see 1D spectrum on top). (C) Superposition of TOCSY (brown) and ROESY spectrum (black-red for positive-negative signals) with the assignment of aromatic protons of PIQ-4m. Two exchange crosspeaks of isochronous phenyl protons H14/H15 with their symmetry-related H13/H12 protons are circled in red.

Table S10. ^{1}H and ^{13}C NMR chemical shifts δ for a *QD2*-I - PIQ-4m 1:1 complex[a]

δ / ppm	H8/H6	H1/H3	H1'	H2'/H2"	H3'	H5/H2/Me	C8/C6	C2
G1	7.43	12.14	6.01	2.80/2.94	4.98	-	141.40	-
G2	8.11	12.16	5.99	2.97/2.29	5.10	-	137.84	-
T3	7.80	n.d.	6.13	2.14/2.50	4.83	1.92	139.32	-
T4	7.04	n.d.	5.91	1.97/2.52	4.82	0.98	137.62	-
G5	7.25	12.17	5.89	3.36/2.84	4.82	-	142.70	-
G6	7.85	11.09	5.51	2.69/2.25	5.06	-	137.86	-
C7	7.30	-	5.09	2.29/2.29	4.80	5.17	143.31	-
G8	7.83	12.62	5.90	2.60/2.69	4.98	-	138.19	-
C9	7.17	-	5.53	1.82/2.23	4.75	5.28	143.51	-
G10	7.78	12.66	5.35	2.56/2.64	4.91	-	138.20	-
A11	7.85	-	5.84	2.24/2.56	4.91	7.52	140.93	154.46
A12	7.41	-	5.84	2.01/2.38	4.83	7.65	139.88	154.93
G13	8.00	n.d.	5.34	2.58/2.30	4.76	-	138.97	-
C14	7.16	-	5.66	1.55/2.00	4.37	5.17	143.50	-
A15	8.04	-	6.26	2.95/2.89	4.77	8.00	143.05	155.39
T16	7.35	13.23	5.63	2.06/2.44	4.69	1.78	139.35	-
T17	7.35	13.81	5.98	2.12/2.41	4.80	1.56	139.35	-
C18	7.38	-	5.52	1.95/2.26	4.74	5.56	143.50	-
G19	7.71	12.73	5.62	2.43/2.49	4.81	-	138.13	-
C20	6.89	-	5.61	1.28/1.99	4.67	4.96	142.04	-
G21	7.66	12.03	5.29	2.56/2.61	4.99	-	138.28	-
G22	7.17	10.82	5.83	3.24/2.66	4.89	-	142.21	-
G23	8.04	11.53	5.85	2.87/2.21	5.02	-	137.74	-
T24	7.74	n.d.	6.11	2.10/2.47	4.80	1.86	139.27	-
T25	6.99	n.d.	5.90	1.91/2.51	4.79	0.86	137.62	-
G26	7.39	11.72	5.96	3.30/2.80	4.82	-	142.19	-
G27	8.04	11.85	6.06	2.71/2.34	4.76	-	137.74	-

[a]At 30 ºC in 20 mM potassium phosphate buffer, 100 mM KCl, pH 7.0.

Table S11. ^{1}H NMR chemical shifts δ (ppm) of PIQ-4m bound to *QD2-l*[a]

H1	H2	H3	H4a	H5a	H6	H7	H8	H9a	10-NH
7.01	6.90	7.38	2.61	4.73	7.76	6.8	7.00	1.96	10.33
H12	H13	H14	H15	16-NH	H17	H18	H19	H20/H22	H21/H23
7.48	7.59	8.03	8.03	8.30	3.38/3.01	1.87	3.06	3.13	1.19

[a]At 30 ºC in 20 mM potassium phosphate buffer, 100 mM KCl, pH 7.0.

Table S12. Glycosidic torsion angles χ (°) at Q-D interfaces without and with an intercalated ligand

	Q-D interface	5'-G-C-3'<br>⋮ ⋮<br>3'-G-G-5'		
Q-D hybrid (PDB ID)	χ 5'-G (G4)	χ C-3'	χ 5'-G	χ G-3' (G4)
free *QD3-sbl* (7PNE)	(G18)	(C19)	(G35)	(*syn*-G36)
	246.1 ± 7.9	248.5 ± 4.6	251.1 ± 5.7	62.6 ± 4.3
QD3-sbl with SYUIQ-5 (7PNG)	(G18)	(C19)	(G35)	(*syn*-G36)
	238.7 ± 11.4	266.5 ± 6.6	254.1 ± 14.8	66.1 ± 5.3
QD3-sbl with Phen-DC3 (8ABD)	(G18)	(C19)	(G35)	(syn-G36)
	251.7 ± 6.5	271.6 ± 6.0	261.3 ± 7.5	78.2 ± 12.4
free *QD2-l* (2M8Z)	(G6)	(C7)	(G21)	(*syn*-G22)
	241.2 ± 3.4	237.1 ± 2.6	241.4 ± 1.6	54.4 ± 1.2
QD2-l with PIQ-4m (8ABN)	(G6)	(C7)	(G21)	(syn-G22)
	254.6 ± 2.8	275.0 ± 1.8	266.9 ± 0.7	68.1 ± 2.0

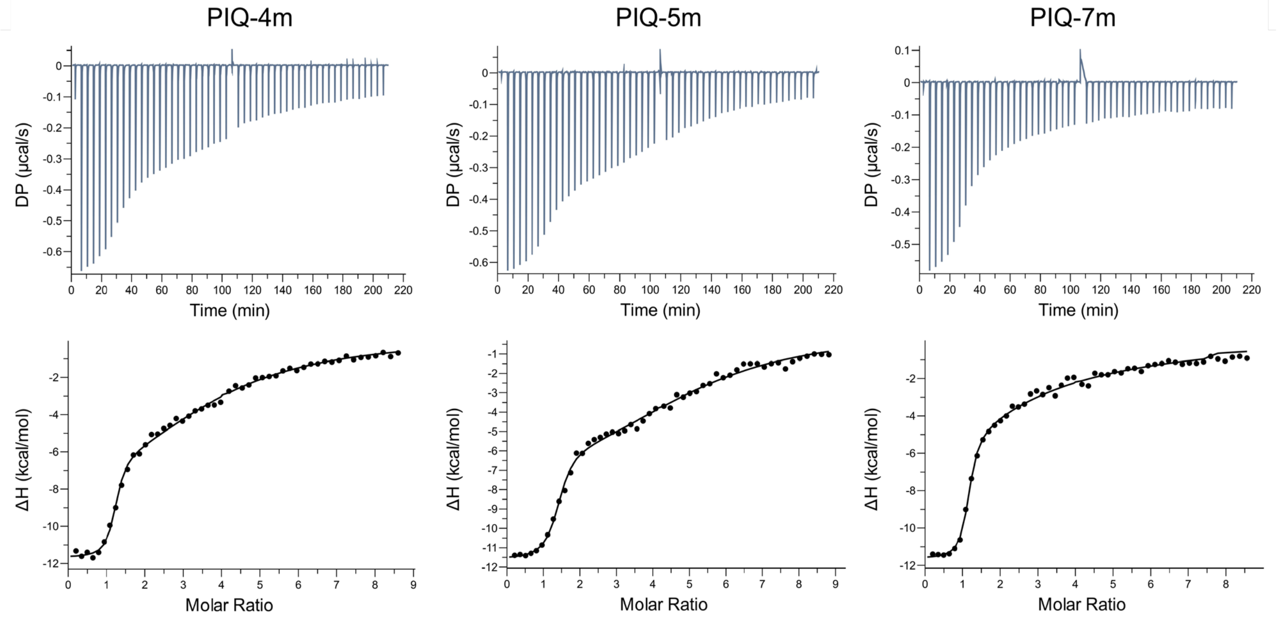

Figure S20. Representative ITC thermograms for the titration of PIQ-derivatives PIQ-4m, PIQ-5m, and PIQ-7m to the *QD2-I* hybrid at 40 °C (20 mM potassium phosphate buffer, pH 7.0, 100 mM KCl, 5 % DMSO). Upper and lower panels show the heat released for each injection step and the blank-corrected normalized heat versus molar ratio. Curves were fitted based on a model with two sets of binding sites.

Table S13. ITC-derived binding parameters of PIQ derivatives with different aliphatic side chains when binding to *QD2-I* at 40 °C[a]

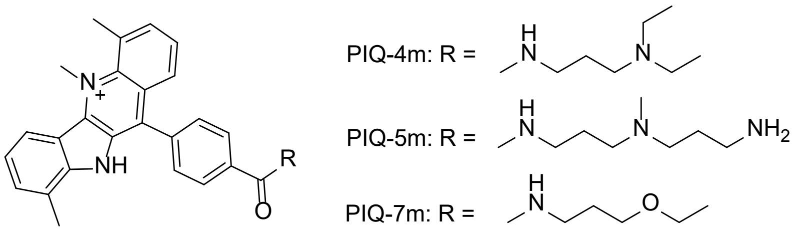

ligand	n	K_a (M^{-1})	ΔH^o (kcal/mol)	$-T\Delta S^o$ (kcal/mol)[b]
PIQ-4m	1.1 ± 0.1	$8.3 \cdot 10^6 \pm 1.4 \cdot 10^6$	-11.5 ± 0.3	1.6 ± 0.3
	5.0 ± 0.6	$2.5 \cdot 10^4 \pm 1.2 \cdot 10^4$	-6.5 ± 0.5	0.2 ± 0.8
PIQ-5m	1.3 ± 0.1	$7.9 \cdot 10^6 \pm 0.9 \cdot 10^6$	-12.0 ± 0.4	2.2 ± 0.4
	5.0 ± 0.4	$5.2 \cdot 10^4 \pm 1.1 \cdot 10^4$	-6.2 ± 0.2	-0.5 ± 0.3
PIQ-7m	1.1 ± 0.1	$2.6 \cdot 10^6 \pm 0.7 \cdot 10^6$	-12.1 ± 0.3	2.9 ± 0.4
	6.0 ± 0.3	$8.7 \cdot 10^3 \pm 0.5 \cdot 10^3$	-5.3 ± 0.2	-0.3 ± 0.2

[a]Average values with root-mean-square deviations obtained from three independent measurements in 20 mM potassium phosphate buffer, pH 7.0, 100 mM KCl, 5% DMSO; data were fitted with two sets of binding sites with fit parameters of the second lower-affinity binding shown on a grey background. [b]$-T\Delta S^o = \Delta G^o - \Delta H^o$ with $\Delta G^o = -RT\ln K_a$.
